# Discovery of an Aldo-Keto reductase 1C3 (AKR1C3) degrader

**DOI:** 10.1038/s42004-024-01177-4

**Published:** 2024-04-29

**Authors:** Angelica V. Carmona, Shirisha Jonnalagadda, Alfie M. Case, Krishnaiah Maddeboina, Sravan K. Jonnalagadda, Louise F. Dow, Ling Duan, Trevor M. Penning, Paul C. Trippier

**Affiliations:** 1 Department of Pharmaceutical Sciences, College of Pharmacy, University of Nebraska Medical Center, Omaha, NE 68106 USA; 2 Center of Excellence in Environmental Toxicology, Department of Systems Pharmacology and Translational Therapeutics, University of Pennsylvania, Philadelphia, PA 19104 USA; 3 Fred & Pamela Buffett Cancer Center, University of Nebraska Medical Center, Omaha, NE 68106 USA; 4 UNMC Center for Drug Design and Innovation, University of Nebraska Medical Center, Omaha, NE 68106 USA

**Keywords:** Chemical tools, Chemical tools, Structure-based drug design

## Abstract

Aldo-keto reductase 1C3 (AKR1C3) is a protein upregulated in prostate cancer, hematological malignancies, and other cancers where it contributes to proliferation and chemotherapeutic resistance. Androgen receptor splice variant 7 (ARv7) is the most common mutation of the AR receptor that confers resistance to clinical androgen receptor signalling inhibitors in castration-resistant prostate cancer. AKR1C3 interacts with ARv7 promoting stabilization. Herein we report the discovery of the first-in-class AKR1C3 Proteolysis-Targeting Chimera (PROTAC) degrader. This first-generation degrader potently reduced AKR1C3 expression in 22Rv1 prostate cancer cells with a half-maximal degradation concentration (DC_50_) of 52 nM. Gratifyingly, concomitant degradation of ARv7 was observed with a DC_50_ = 70 nM, along with degradation of the AKR1C3 isoforms AKR1C1 and AKR1C2 to a lesser extent. This compound represents a highly useful chemical tool and a promising strategy for prostate cancer intervention.

## Abstract (web summary)

Aldo-keto reductase 1C3 (AKR1C3) is known to be a cancer biomarker correlated to androgen synthesis, and causes drug resistance by direct action on chemotherapeutics and by stabilizing AR splice variant 7 (ARv7). While selective inhibitors against AKR1C3 are developed, the ARv7 is thought to be undruggable. Here, the authors develop an AKR1C3-ARv7 dual PROTAC degrader based on a selective inhibitor against AKR1C3, showing the potential for degradation of AKR1C3 and ARv7 simultaneously.

## Introduction

Aldo-keto reductase 1 C3 (AKR1C3), also known as type 5 17β-hydroxysteroid dehydrogenase (17β-HSD), is a member of the aldo-keto reductase superfamily of proteins. It is a soluble monomeric NAD(P)(H)-dependent oxidoreductase, which catalyzes the reduction of carbonyl groups to secondary alcohols^1^. The protein is overexpressed in many hormone-dependent cancers and hematological malignancies^2–5^. AKR1C3 plays a vital role in regulating myeloid and lymphoblast cell differentiation, proliferation, and apoptosis of hematological malignant cells; catalyzes the formation of potent androgens responsible for tumor proliferation and aggression in prostate and other hormone-dependent cancers and through its reductase activity, contributes to drug resistance against a wide variety of chemotherapeutics^6–9^.

The AKR1C family includes the related AKR1C1 and AKR1C2 proteins which share >84% sequence identity with AKR1C3. The requirement for selective inhibition of AKR1C3 for therapeutic effect differs depending on cancer type and stage. While selective inhibition of AKR1C3 in castration-resistant prostate cancer is desirable for anti-proliferative effects^7^ (via suppression of androgen synthesis) pan-AKR1C inhibitors could counter drug resistance. All three enzymes have been reported to play a role in chemotherapy resistance in T-cell acute lymphoblastic leukemia (T-ALL)^10^, and a pan-AKR1C inhibitor outperformed the AKR1C3 selective inhibitor medroxyprogesterone acetate in reducing cell viability in several acute myeloid leukemia (AML) cell lines. Contrary to these studies, we have shown that more potent AKR1C3 inhibitors with greater isoform selectivity reverse drug resistance to etoposide^11^, daunorubicin, and cytarabine in AML cells and patient-derived T-ALL cells^10^.

An emerging role of AKR1C3 to stabilize the full-length androgen receptor (AR-FL) and AR splice variants^12^, particularly AR splice variant 7 (ARv7), the most common splice variant, has been reported^13,14^. Constitutively active AR splice variants such as ARv7 have been shown to be critical mechanisms in promoting resistance to the androgen receptor signaling inhibitors (ARSIs) enzalutamide, darolutamide, and apalutamide^15–17^. AKR1C3 interaction with ARv7 in castration-resistant prostate cancer cells inhibits ARv7 degradation. Ubiquitin binding to ARv7 and AR-FL is known to increase, and the receptors degraded, when AKR1C3 expression is low. Degradation is significantly slowed with overexpression of AKR1C3^17^. These observations led us to theorize that degradation of AKR1C3 using a suitably functionalized small molecule inhibitor would enable dual degradation of AKR1C3 and ARv7. Thus, countering two mechanisms of resistance to the ARSI clinical agent Enzalutamide.

Proteolysis-targeting chimeras (PROTACs) are heterobifunctional molecules consisting of a ligand that binds to the protein of interest connected by a linker to a ligand that binds to and recruits the E3 ubiquitin ligase. Once the target protein and either a component of E3 ubiquitin ligase or the E2 ligase are bound, a ternary complex is formed, followed by polyubiquitination and subsequent degradation of the target protein by the 26S proteasome; the PROTAC is then recycled to carry out successive rounds of polyubiquitination^18–20^. A PROTAC degradation strategy possesses several advantages over small molecule inhibition: the ability to target undruggable proteins e.g. ARv7, induce complete removal of the target protein to overcome drug resistance that might be mediated via mutations or protein overexpression and accumulation, and have catalytic activity at sub-stoichiometric concentrations. The PROTAC may also have increased potency and selectivity, achievable via protein-protein interactions between the protein of interest and E3 ubiquitin ligase and prolonged pharmacodynamic effects without continuous PROTAC exposure^21–24^. An androgen receptor degrader utilizing the PROTAC concept has recently entered clinical trial (NCT03888612). However, no degraders targeting AR splice variants have been progressed to this stage. Herein, we report the discovery and evaluation of the first AKR1C3-ARv7 dual degrader.

**Fig. 1:**
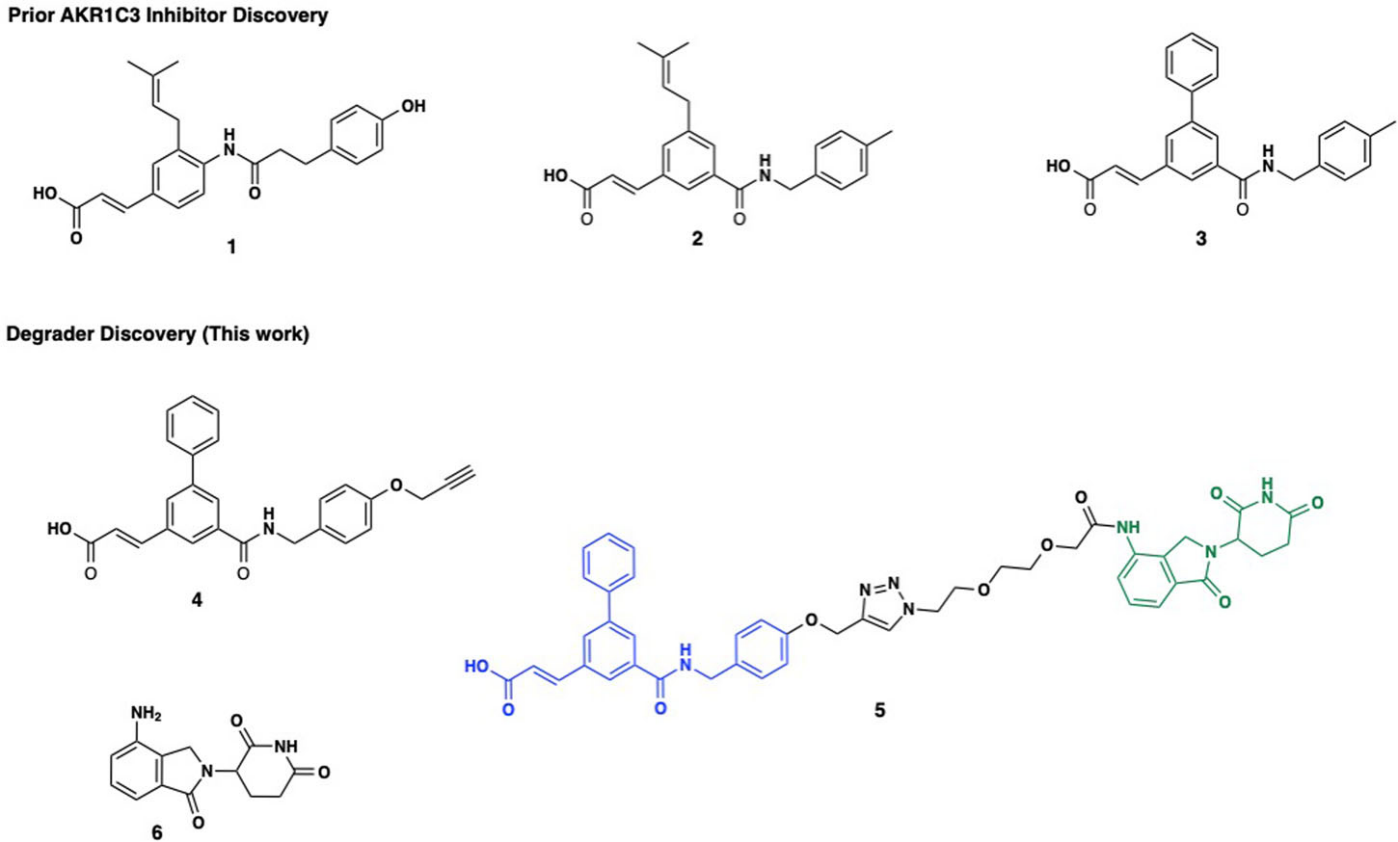
Structures of selected AKR1C3 inhibitors, PROTAC and Lenalidomide. Chemical structures of representative small molecule AKR1C3 inhibitors (**1**–**3**), PROTAC warhead **4**, the assembled PROTAC **5** with warhead (Blue), triazole-PEG2 linker, and E3-ligase lenalidomide (green, **6**).

Prior efforts from our group identified phenolic AKR1C3 inhibitor **1**, which possessed high activity with an IC_50_ = 110 nM with 127-fold selectivity for AKR1C3 over AKR1C2 (Fig. 1). A retro amide isosteric switch and truncation afforded access to inhibitor **2** possessing AKR1C3 IC_50_ = 70 nM with >2800-fold selectivity for AKR1C3 over AKR1C1 and AKR1C2^10^. Further structure-activity relationship studies identified the biphenyl derivative **3** with an AKR1C3 IC_50_ = 43 nM with >2300-fold selectivity for AKR1C3 over AKR1C2, and is one of the most potent and selective inhibitors of AKR1C3 known to date (Fig. 1)^10,25^.

Herein, we report the design, synthesis, and characterization of what is to the best of our knowledge, the first designed PROTAC degrader of AKR1C3 which concomitantly acts to degrade ARv7, a receptor thought to be undruggable. PROTAC **5**, provides significant effect to degrade AKR1C3 above and beyond the effect of small molecule inhibitors which results in loss of cancer cell viability and significantly sensitizes previously resistant prostate cancer cell lines to the action of the clinical gold standard chemotherapeutic Enzalutamide.

## Results

### Small molecule AKR1C3 inhibitors degrade AKR1C3 and ARv7 expression at high concentration

**Fig. 2:**
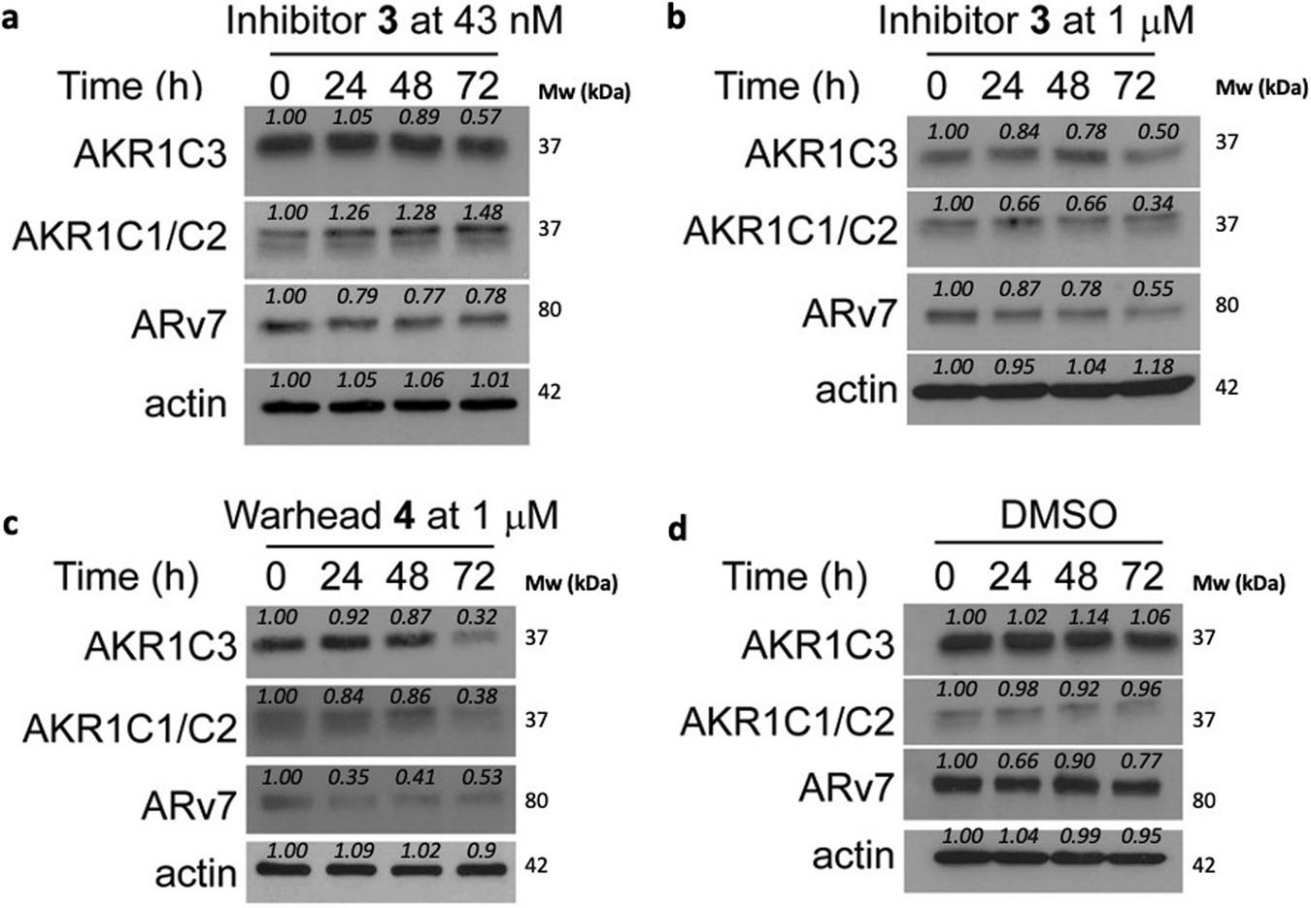
Degradation of AKR1C3 in prostate cancer cells by small molecule inhibitors. Representative Western blots of AKR1C3, AKR1C1/C2 and ARv7 protein expression in 22Rv1 prostate cancer cells treated with (**a**) AKR1C3 inhibitor **3** at 43 nM; (**b**) AKR1C3 inhibitor **3** at 1 µM; (**c**) AKR1C3 inhibitor warhead **4** at 1 µM and (**d**) DMSO; for 0, 24, 48 and 72 h. Images representative of at least two technical replicates performed in duplicate. Quantification in Supplementary Fig. S1.

Previous studies have shown that certain small molecule inhibitors of AKR1C3 e.g., indomethacin and BMT4-158 degrade the target^12,26^. To determine the magnitude of degradation of target proteins resulting from exposure to our small molecule inhibitor scaffold, we investigated the ability of inhibitor **3** and warhead **4** (Fig. 1) to degrade AKR1C3, AKR1C1/2, and ARv7 in 22Rv1 prostate cancer cells (Fig. 2). Inhibitor **3**, when used at its AKR1C3 IC_50_ concentration of 43 nM, provided no evidence of degradation of AKR1C3 or AKR1C1/2 up to 48 h, but did induce degradation of AKR1C3 and ARv7 at 72 h (Fig. 2a, quantification Fig. S1a), approximately 20% ARv7 degradation was observed at 24 h post treatment, which remained constant across all time points. At 1 µM concentration, **3** showed dose-dependent effects; AKR1C3 was reproducibly degraded by approximately 50% at 72 h (Fig. 2b) with a time-dependent increase in degradation from 24–72 h (Fig. S1b). As expected, through the known stabilizing effect of AKR1C3 upon ARv7, inhibition of AKR1C3 led to degradation of ARv7 in a time-dependent manner, with approximately 50% degradation at 72 h (Fig. S1b). Interestingly, AKR1C1/2 was also degraded by **3**, despite this compound having an IC_50_ value for AKR1C1/2 inhibition >100 µM. Warhead **4** at 1 µM proved to be a more efficient degrader at 72 h than **3**. Time-dependent degradation of AKR1C3, AKR1C1/2, and ARv7 was apparent with approximately 70% degradation of all at 72 h (Fig. 2c, quantification Fig. S1c). To ensure the observed degradation resulted from AKR1C3 inhibition and not cellular protein variation over time, degradation was monitored over 72 h following treatment with DMSO vehicle (Fig. 2d, quantification Fig. S1d). Expression levels for the proteins of interest remained constant.

### Design and synthesis of PROTAC degrader 5

**Fig. 3:**
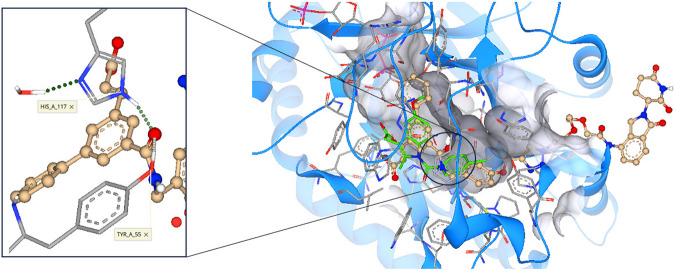
Docking predictions of PROTAC 5 binding to AKR1C3. The PROTAC warhead (gold) is predicted to bind into the same SP1 pocket of AKR1C3 (PDB ID: 3UG8) as the known inhibitor indomethacin (green, overlay). A PEG2 linker with a triazole anchor point predicts sufficient length to engender solvent exposure of the E3 ligase ligand.

Having established that our small molecule inhibitors can degrade AKR1C3 and ARv7, we sought to design a PROTAC functionalized degrader theorizing it would possess enhanced degradation efficiency. Cognizant that PROTACs are, by their nature, considerably larger than prior AKR1C3 small molecule inhibitors, we conducted molecular docking studies to determine the fit of the warhead into the SP1 binding site of AKR1C3, optimal linker attachment point, and linker length requirement to ensure solvent exposure of the E3 ligase ligand. The published crystal structure of AKR1C3 bound with indomethacin (PDB ID: 3UG8) was employed, the ligand removed, and a binding site of 30 amino acid residues was defined. We designed a functionalized degrader based around inhibitor **3**, by exploiting the alcohol moiety of **1** as a linker point, the improved stability of amide **2**, and the high potency of **3** to provide a hybrid AKR1C3 inhibitor warhead (**4**) linked to the cereblon ligand lenalidomide (**6**) to produce PROTAC **5** (Fig. 1)^27^. The PROTAC was prepared and docked using SeeSAR 12.1 software (BioSolveIT). Gratifyingly, the docking revealed three predictions for the proposed PROTAC; (i) retention of hydrogen bond formation between the warhead amide carbonyl and the critical amino acid residues Tyr55 and His117 within the AKR1C3 binding site^10^, predicting retention of AKR1C3 inhibition. (ii) absence of a role for the triazole anchor in binding to the AKR1C3 active site, a concern arising from recently disclosed hydroxy triazole AKR1C3 inhibitors^28^. (iii) the length of the linker was sufficient so that the PROTAC will bind to and stabilize a ternary complex and, as a result, affecting polyubiquitination and subsequent degradation of the target protein by the 26S proteasome^29^. Overall the structure of PROTAC **5** is predicted to bind into the same AKR1C3 site as the small molecule inhibitor and engender solvent exposure of the E3 ligase ligand (Fig. 3).

**Fig. 4:**
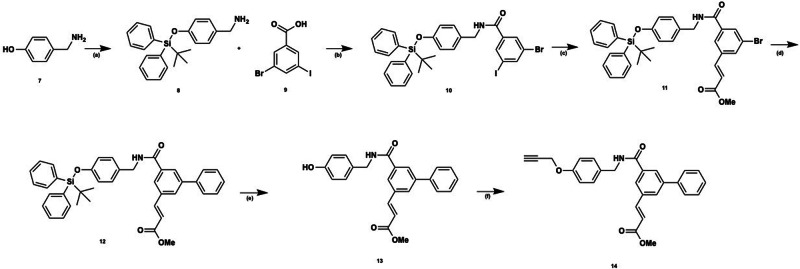
Synthesis of AKR1C3 degrader warhead 14. Reagents and conditions: (**a**) *tert*-butylchlorodiphenylsilane (TBDPSiCl), imidazole, tetrahydrofuran (THF), rt; (**b**) 1-(3-dimethylaminopropyl)-3-ethylcarbodiimide hydrochloride (EDC·HCl), 1-hydroxybenzotriazaole hydrate (HOBt hydrate), *N*,*N*-diisopropylethylamine (DIPEA), dichloromethane (DCM), 0 °C – rt; (**c**) methyl acrylate, Pd(OAc)_2_, P(Ph)_3_, triethylamine (NEt_3_), toluene, 110 °C; (**d**) phenyl boronic acid, Pd(dppf)Cl_2_⋅CH_2_Cl_2_, Cs_2_CO_3_, toluene, 110 °C; (**e**) tetrabutylammonium fluoride (TBAF), THF, 0 °C, 40 min; (**f**) propargyl bromide, Cs_2_CO_3_, dimethylformamide (DMF), 60 °C.

Synthesis of PROTAC **5** involved coupling the methyl ester derivative of the propargyl warhead **14** and azide-diethylene glycol functionalized lenalidomide **18**, (Fig. 4). The warhead was accessed via our previously optimized route, with the *tert*-butyldiphenylsilyl (TBDPS) protecting group essential for obtaining high yield of intermediate **10**^10,30^. This protecting group was removed at a later stage in quantitative yield by exposure to TBAF^31^ to afford phenol **13**.

**Fig. 5:**
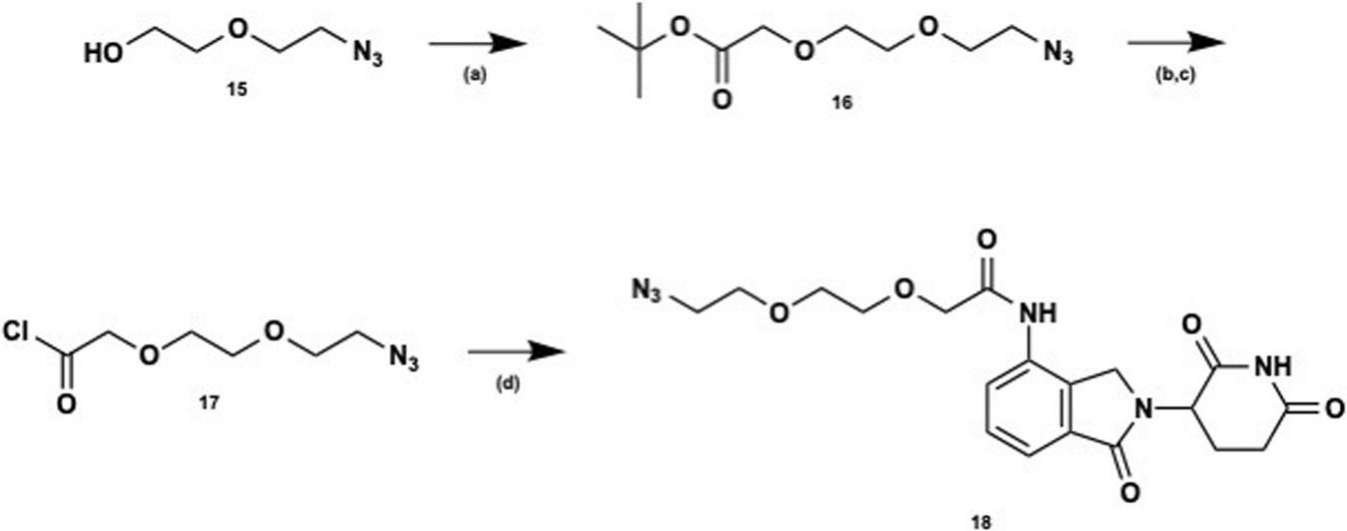
Synthesis of E3 ligase ligand lenalidomide and linker 18. Reagents and conditions: (**a**) *tert*-butyl bromoacetate, sodium hydride (NaH), THF, 0 °C (30 min) to rt; (**b**) trifluoroacetic acid (TFA), DCM, rt; (**c**) SOCl_2_, DCM, rt; (**d**) lenalidomide, *N*-methyl-2-pyrrolidone (NMP), rt.

**Fig. 6:**
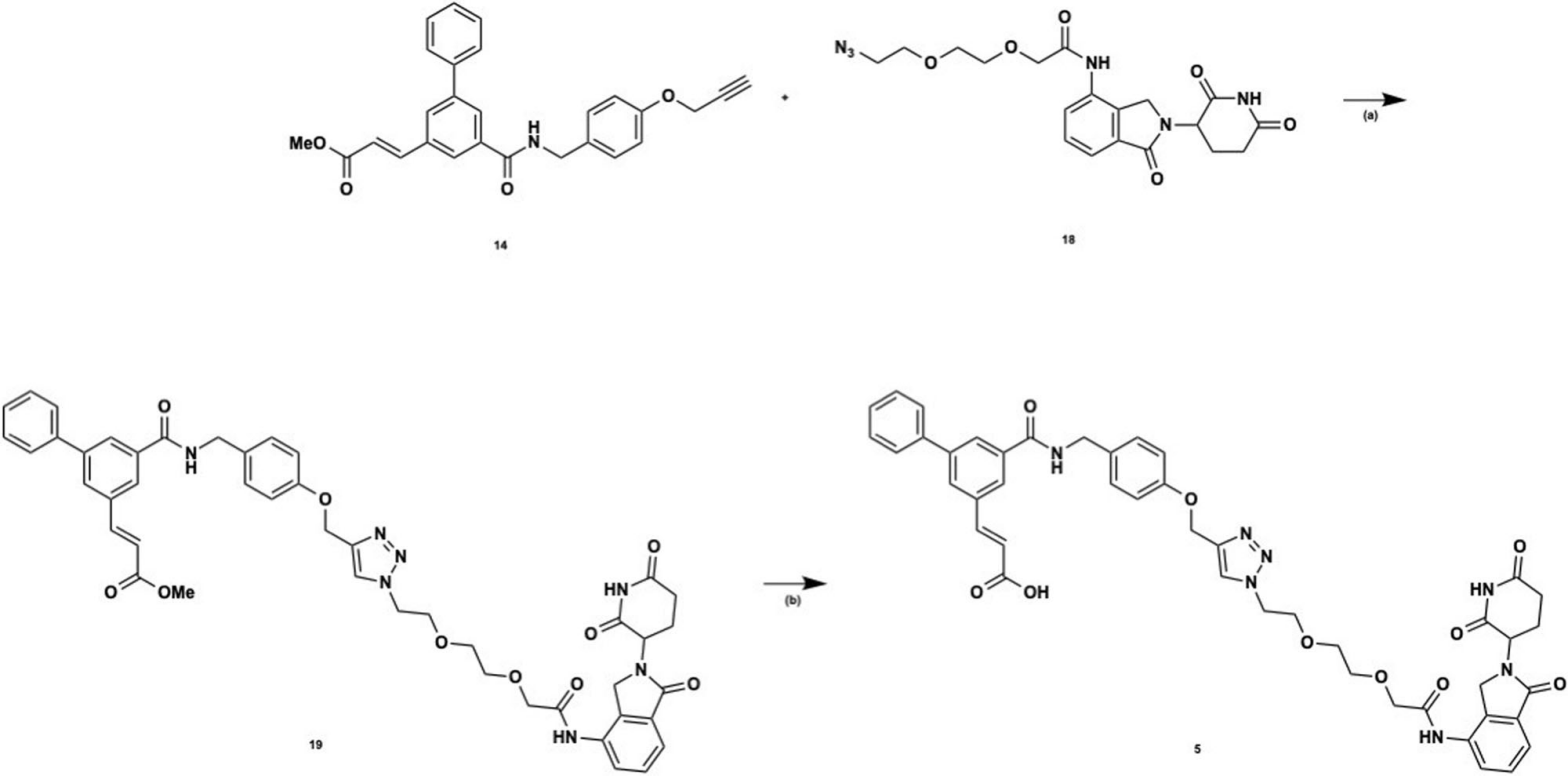
Synthesis of PROTAC degrader 5. Reagents and conditions: (**a**) sodium ascorbate, copper sulfate pentahydrate (CuSO_4_⋅5H_2_O), CH_2_Cl_2_:MeOH:H_2_O, rt; (**b**) 1 N NaOH, MeOH:THF, reflux.

Functionalized lenalidomide was accessed as depicted (Fig. 5) from commercially available azide **15** which underwent nucleophilic substitution with *tert*-butyl bromoacetate to afford PEG2 linker **16**. The *tert*-butyl ester underwent acid-catalyzed hydrolysis and subsequent acid chloride formation. Acyl chloride **17** was then coupled to lenalidomide to afford linker/E3 ligase ligand **18**. Warhead **14** and E3 ligase ligand **18** underwent Huisgen cycloaddition^32^, to afford the desired triazole and provide PROTAC **5** in moderate overall yield (Fig. 6). For full synthetic methods and compound characterization see the Supplementary Methods.

### PROTAC functionalization retains AKR1C3 potency but ameliorates selectivity

With PROTAC **5** in hand, we first determined AKR1C3 inhibition activity and its ability to ameliorate the survival of 22Rv1 prostate cancer cells that express high levels of AKR1C3^7^. Warhead compound **4** possessed AKR1C3 IC_50_ = 62 ± 1 nM and PROTAC **5** possessed an IC_50_ = 77 ± 2 nM (Fig. S2), both equipotent with small molecule inhibitor **2** and slightly less potent than inhibitor **3** (IC_50_ = 43 nM). Selectivity over AKR1C1/2 however, was diminished by 15-fold over **3**, wherein selectivity of 146- and 116-fold, respectively for **4** and **5** was determined. PROTAC **5** was found to have similar activity to ameliorate 22Rv1 cell viability as AKR1C3 inhibitor **3** (IC_50_ = 49.3 ± 1.40 µM and 40.6 ± 10.1 µM respectively) (Fig. S3a, b). The E3 ligase ligand, lenalidomide **6**, a clinical antineoplastic, exhibited no activity in the 22Rv1 prostate cancer cell line (IC_50_ > 100 µM), discounting any contribution from this moiety to the activity of **5** (Fig. S3c). The disconnection between biochemical IC_50_ and cellular inhibition effect can be rationalized by the cell penetration issues presented by a carboxylic acid containing compound, and this has been previously observed with other free acid AKR1C3 inhibitors and rectified with a prodrug strategy ^25^.

### PROTAC 5 is an effective degrader of AKR1C3, AKR1C1/2, and ARv7 in a concentration and time-dependent manner

**Fig. 7:**
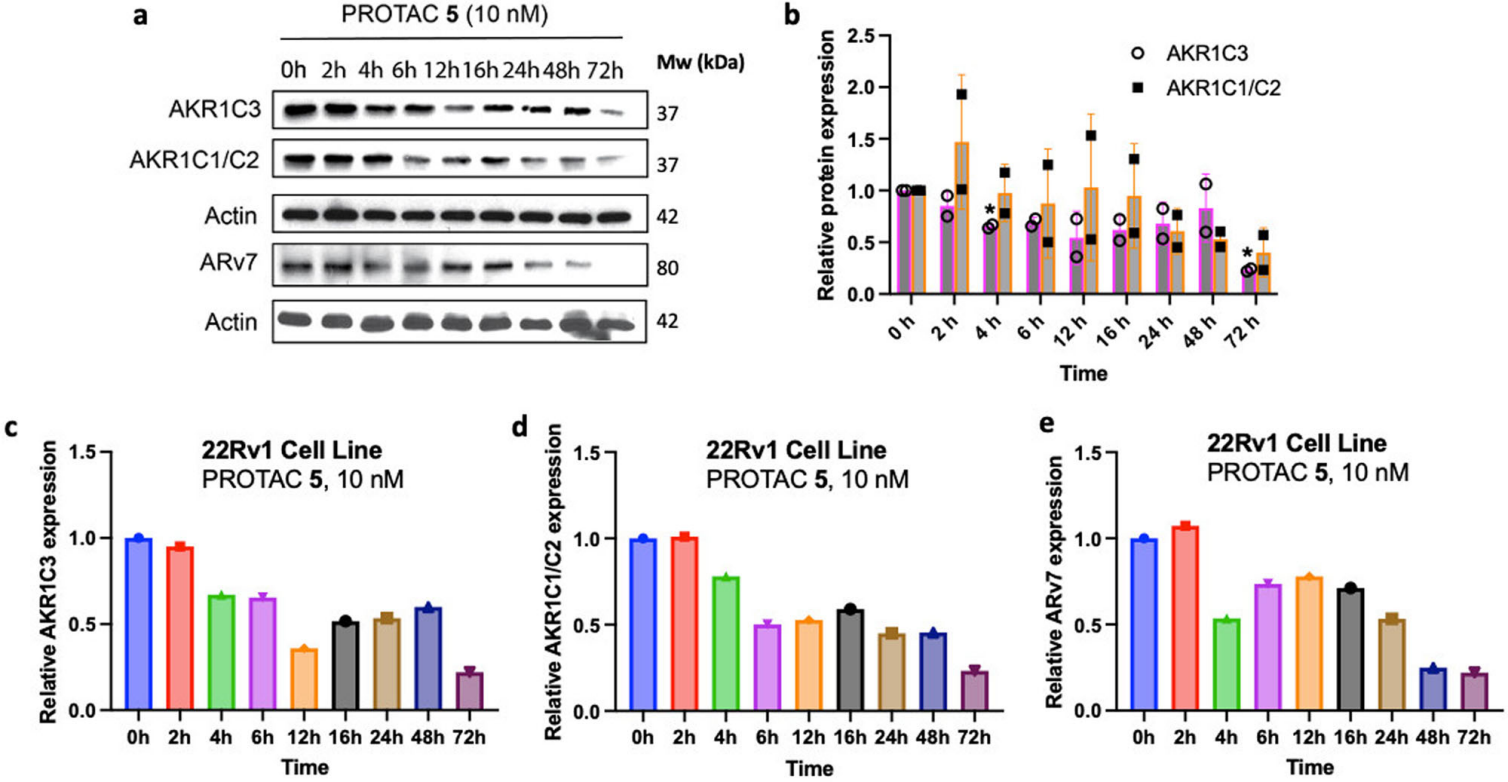
Degradation of AKR1C3, AKR1C1/C2 and ARv7 by PROTAC 5. **a** Time study of degradation of AKR1C3, AKR1C1/C2, and ARv7 upon treatment of 5 (10 nM) at different time points (0, 2, 4, 6, 12, 16, 24, 48, and 72 h). Blots are representative of two separate experiments; (**b**) combined quantification of a and replicates for AKR1C3 and AKR1C1/C2 expression. Data obtained from two technical replicates performed in duplicate is represented as the mean ± standard deviation. *p < 0.05 by two-tailed, unpaired Mann–Whitney test; (**c**) Quantification of a for AKR1C3 expression; (**d**) quantification of a for AKR1C1/C2 expression; (**e**) quantification of a for ARv7 expression. Relative expression is normalized to actin.

We determined the time-dependent degradation effect of PROTAC **5** on target proteins at 0, 2, 4, 6, 12, 16, 24, 48, and 72 h. A time-dependent trend of decreasing AKR1C3 levels starting at 24 h post-treatment, through 72 h post-treatment, was observed with just 1 nM treatment of **5** but was not significant (Fig. S4). Gratifyingly, significant degradation of AKR1C3 was observed when the concentration of **5** was increased to 10 nM (Fig. 7). Degradation of AKR1C3 was observed from 4 h post-treatment onwards, with maximal degradation of approximately 75% was observed at 72 h (Fig. 7a–c), indicating a dose-dependent degradation effect. Treatment of 22Rv1 cells with PROTAC **5** results in amelioration of ARv7 expression at 72 h (Fig. 7a, e). PROTAC **5** provided a trend of degradation of AKR1C1/C2 in a time-dependent manner (Fig. 7d), but to a lesser extent than AKR1C3. This phenomenon is also observed with highly specific AKR1C3 inhibitor **3** ( > 2300-fold selectivity for AKR1C3 versus **5** with 116-fold selectivity for AKR1C3). At concentrations higher than 1 µM the ‘hook effect’ begins (Fig. S5). Wherein PROTAC compounds can saturate binding sites on either the protein of interest or the E3 ligase, without forming the required ternary complex resulting in decreased activity ^33^. Observation of the hook effect provides evidence that compound **5** is indeed acting by the PROTAC mechanism.

### PROTAC 5 degrades AKR1C3 to a greater extent than a small molecule inhibitor and is proteasome dependent

**Fig. 8:**
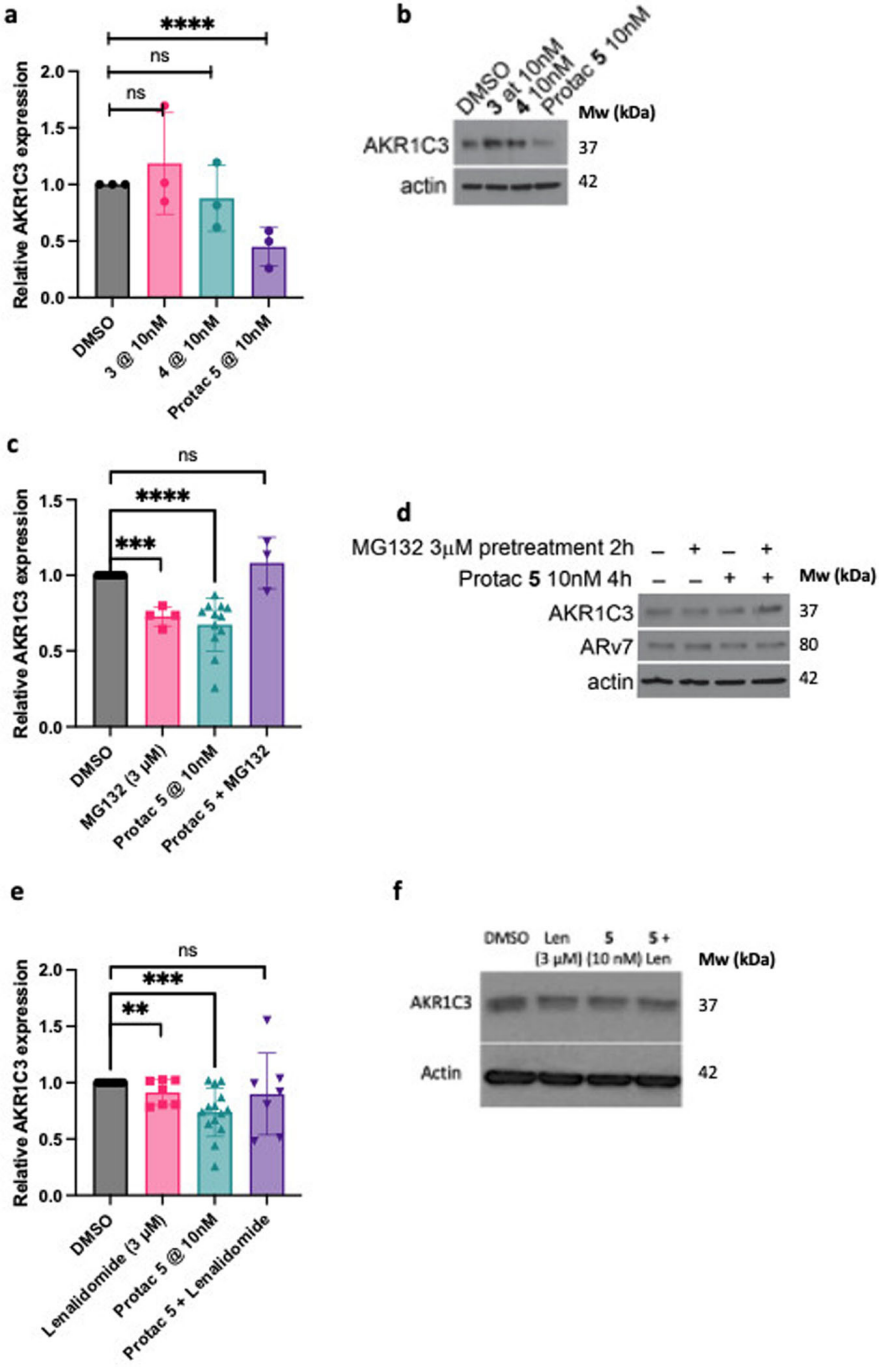
Mechanistic studies of PROTAC 5 degradation effect in 22Rv1 prostate cancer cells. **a** Quantification (*n* = 3) and (**b**) Selected Western blot image of AKR1C3 expression after 72 h treatment of degrader **5** versus small molecule AKR1C3 inhibitors **3** and **4** at 10 nM concentration; (**c**) Quantification (*n* = 12 DMSO, *n* = 4 MG132, *n* = 12 PROTAC, *n* = 3 MG132 & PROTAC) and (**d**) selected Western blot image of effect on protein degradation with 2 h pretreatment of DMSO or the proteasome inhibitor MG132 (3 µM). Cells were then treated with PROTAC **5** at 10 nM for 4; (**e**) Quantification (*n* = 14 DMSO, *n* = 7 Lenalidomide, *n* = 14 PROTAC, *n* = 7 Lenalidomide & PROTAC) and (**f**) selected Western blot image of effect on protein degradation with 2 h pretreatment of DMSO or lenalidomide (3 µM). Cells were then treated with PROTAC **5** at 10 nM for 72 h. Data obtained from at least three replicates is represented as the mean ± SEM. ns, not significant; **p < 0.01; ***p < 0.001; ****p < 0.0001 by unpaired two-tailed *t* test.

PROTAC **5** outperforms small molecule AKR1C3 inhibitors at 10 nM concentrations after 72 h incubation (Fig. 8a). While small molecule AKR1C3 inhibitors **3** and **4** show no significant degradation of AKR1C3 in 22Rv1 cells, PROTAC **5** induced significant degradation of AKR1C3. Calculation of half-maximal degradation concentration (DC_50_) for **5** revealed an AKR1C3 72 h DC_50_ = 52 nM, AKR1C1/C2 DC_50_ = 49 nM, and ARv7 DC_50_ = 70 nM (Fig. S6).

Significant degradation of AKR1C3 protein expression in 22Rv1 cells is observed upon treatment of PROTAC **5** at 10 nM for 4 h (Figs. 7b, 8c). Degradation of AKR1C3 was effectively blocked by pretreatment of the proteasome inhibitor MG132 (Fig. 8c, d) while ARv7 degradation showed a trend of reduced degradation. Longer incubation times with MG132 and higher concentrations were trialed to determine effect at the 72 h time period however, these proved toxic to the cell line.

Pretreatment of 22Rv1 cells with lenalidomide at 3 µM for 2 h results in significant degradation of AKR1C3 but with less significance than PROTAC **5** at 10 nM for 72 h (Fig. 8e, f). Pretreatment of cells with lenalidomide at 3 µM for 2 h and then 10 nM PROTAC **5** for 72 h trends to counter degradation but is not significant. However, the intrinsic degradation of AKR1C3 seen with lenalidomide complicates interpretation of this data, as synergistic or additive degradation effect may be observed. Additionally, lenalidomide has been shown to exert toxic effects in prostate cancer cells^34,35^. Indirect evidence for the involvement of ubiquitination in the degradation of AKR1C3 is provided by the ubiquitin proteasome system dependency required for degradation of AKR1C3 (Fig. 8c, d), the observation of the hook effect at high concentrations of **5** (Fig. S5) and the much greater degradation efficiency of **5** contrasted to small molecule AKR1C3 inhibitors **3** and **4**.

### PROTAC 5 reduces cell viability of in vitro prostate cancer models and potentiates enzalutamide through an AKR1C3-dependant effect

**Fig. 9:**
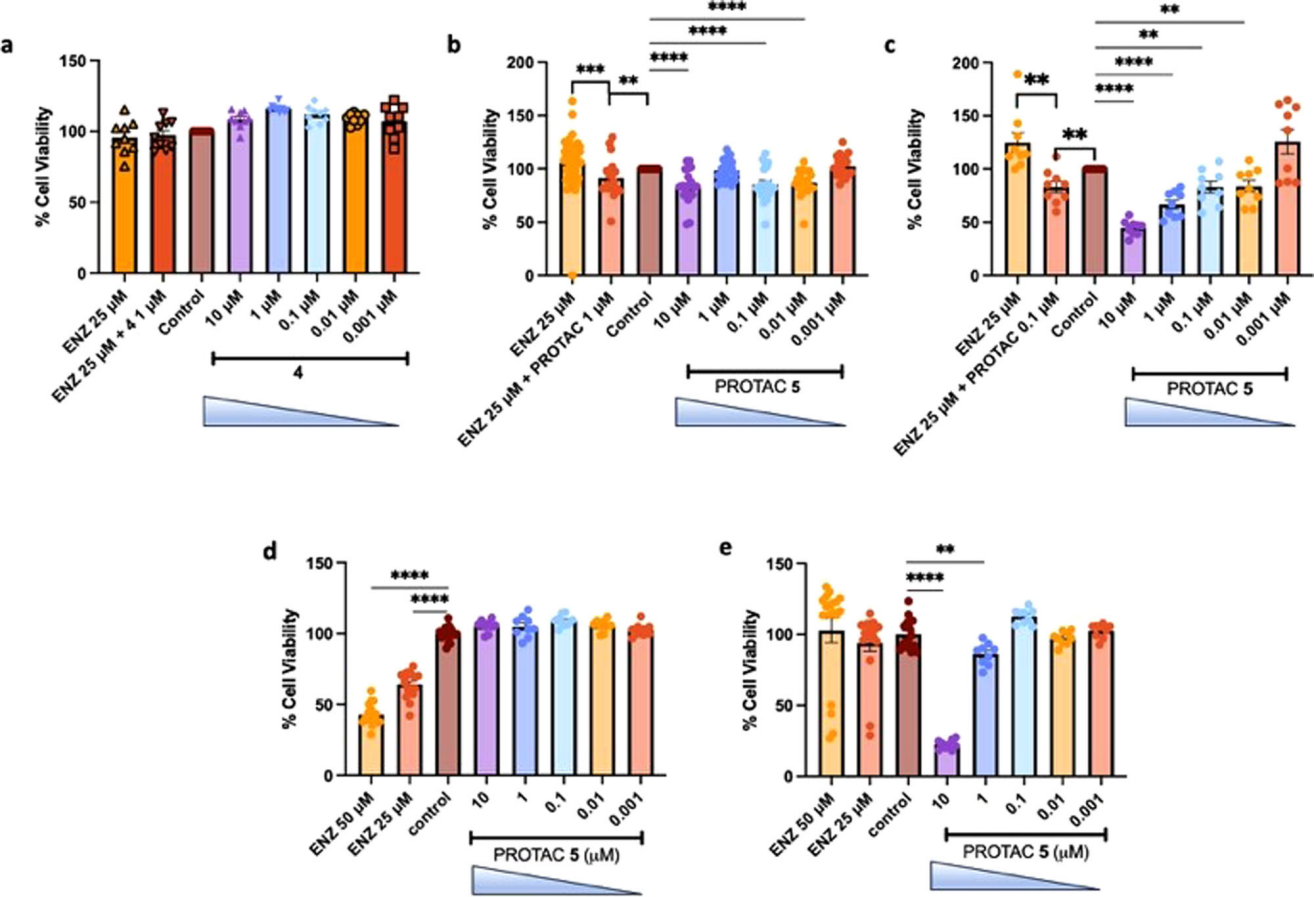
Activity of PROTAC 5 and Warhead 4 on cell viability of prostate cancer cells displaying differential expression of AKR1C3. **a** Effect of warhead compound **4** at indicated concentrations on cell viability of 22Rv1 cells (*n* = 9); (**b**) Effect of PROTAC **5** at indicated concentrations on cell viability of 22Rv1 cells alone and in combination with enzalutamide (ENZ), (*n* = 20 except ENZ 25 µM, *n* = 47); (**c**) Effect of PROTAC **5** at indicated concentrations on cell viability of 22Rv1 cells grown in CSS media (high AKR1C3 expression) alone and in combination with ENZ (*n* = 9); (**d**) Effect of PROTAC **5** and ENZ at indicated concentrations on cell viability of LNCaP cells (AKR1C3 null), (*n* = 18 ENZ 50 µM, 25 µM and control, *n* = 9 each concentration); (**e**) Effect of PROTAC **5** and ENZ at indicated concentrations on cell viability of LNCaP1C3 cells (stably transfected with AKR1C3), (*n* = 18 ENZ 50 µM, 25 µM and control), *n* = 9 each concentration. Data is represented as the mean ± SEM of at least three experiments ran in triplicate. *p < 0.05, **p < 0.01; ***p > 0.001; ****p < 0.0001 by unpaired two-tailed *t* test.

To further determine the effect of PROTAC **5** in in vitro models of prostate cancer and confirm the AKR1C3 target of action we incubated PROTAC **5** and warhead inhibitor **4** in 22Rv1 cells (moderate expression of AKR1C3), 22Rv1 cells grown in charcoal stripped serum (CSS) (high AKR1C3 expression), LNCaP cells (AKR1C3 null) and AKR1C3 stably transfected LNCaP1C3 cells (Fig. 9)^7^, and determined cell viability by the MTT assay. Warhead compound **4**, a small molecule inhibitor of AKR1C3, at concentration from 0.001 to 10 µM affords no reduction of cell viability in 22Rv1 prostate cancer cells after 72 h of treatment (Fig. 9a), despite its AKR1C3 IC_50_ = 62 nM. Enzalutamide (ENZ) at 25 µM provides no effect to reduce cell viability as expected, with the cell line being resistant to the clinical agent by expression of AKR1C3 and ARv7^36^. Combination of 1 µM **4** and ENZ provides no effect. When PROTAC **5** is exposed to 22Rv1 cells for 72 h at concentrations of 0.001–10 µM significant loss of cell viability is observed at 0.01, 0.1 and 10 µM (Fig. 9b). Interestingly, 1 µM treatment does not provide significant loss of cell viability. We postulate this may be due to the hook effect seen with PROTAC **5** (Fig. S5) which begins at 1 µM treatment. While at 10 µM the inherent AKR1C3 inhibition activity of the PROTAC results in some loss of cell viability. ENZ again provides no effect in this cell line. Gratifyingly when PROTAC **5** at 1 µM is combined with ENZ a significant reduction in cell viability is observed, overcoming resistance of the cell line to this clinical chemotherapeutic. Thus, PROTAC **5** is more active than warhead **4** at sensitizing 22Rv1 cells to ENZ, potentially attributable to the AKR1C3 degradation mechanism of action.

When 22RV1 cells are grown in CSS media AKR1C3 expression is significantly enhanced^7,15,17^. Treatment of these cells with PROTAC **5** results in significant and dose-dependent reduction of cell viability from a concentration of just 0.01 µM (Fig. 9c). At 10 µM concentration of PROTAC **5**, 55% reduction in cell viability is observed. Again, these cells prove resistant to 25 µM ENZ but combination with just 0.1 µM PROTAC **5** sensitizes the cells to the action of the clinical chemotherapeutic with significant reduction of cell viability observed.

LNCaP prostate cancer cells do not express AKR1C3 or ARv7^7,11,37^, as expected, treatment of these cells with PROTAC **5** resulted in no observable effects (Fig. 9d). ENZ in this cell line showed significant reduction of cell viability in a dose-dependent manner. Stable transfection of AKR1C3 into LNCaP cells affords LNCaP1C3 cells that highly express AKR1C3^7,38^. Transfection of AKR1C3 affords resistance to the effects of 25 µM ENZ (Fig. 9e). Treatment of these cells with PROTAC **5** results in a dose-dependent reduction of cell viability at 1 and 10 µM (Fig. 9e).

## Discussion

The AKR1C3 protein is known to stabilize the full-length AR (AR-FL) and AR splice variants^12^, preventing degradation. Small molecule inhibition of AKR1C3 has previously been shown to degrade ARv7 via disruption of the AKR1C3/ARv7 co-complex which is stabilized upon overexpression of AKR1C3. When AKR1C3 expression is low, ubiquitin binding to ARv7 and AR-FL increases with concomitant degradation of the AR and/or the AR splice variant^17^. Inhibition of AKR1C3 with a high concentration of small molecule inhibitor (**3** or **4**) in excess of its IC_50_ value leads, over 72 h, to the degradation of AKR1C3, which brings about concomitant degradation of ARv7. Despite these compounds being selective for AKR1C3 inhibition, degradation of AKR1C1/2 is also observed. These small molecule AKR1C3 inhibitors present valuable potential warhead compounds for subsequent design of PROTAC functionalized degraders which would be expected to possess enhanced degradation efficiency.

A PROTAC functionalized AKR1C3 degrader was strategically designed and synthesized guided by molecular modeling to ensure binding to the AKR1C3 SP2 pocket with a suitable exit vector and length of linker to afford solvent exposure of the E3 ubiquitin ligase. Synthesis of an analog of inhibitor **3** with a propargyl ether tether allowed click chemistry attachment of a functionalized lenalidomide-PEG2-azide to afford synthetic access to designed PROTAC **5**. The determined biochemical IC_50_ values of warhead **4** and PROTAC **5** demonstrate that incorporation of the PROTAC functionality had no adverse effect on AKR1C3 inhibition (AKR1C3 IC_50_ = 62 ± 1 nM and 77 ± 2 nM respectively). However, selectivity over AKR1C1/2 for both compounds was ameliorated by approximately 15-fold over **3** (AKR1C3 IC_50_ = 43 nM with >2300-fold selectivity). We postulate that this reduction of selectivity is due to the presence of the oxygen atom on the terminal phenyl ring. Inhibitor **2** possessing a terminal methyl group as in **3** possessed an AKR1C3 IC_50_ = 70 nM with >2800-fold. Introduction of a terminal alcohol (**1**) retains potency (AKR1C3 IC_50_ = 110 nM) but suffers from reduced selectivity of 127-fold for AKR1C3 over AKR1C1/C2. Conversion of the alcohol to the propargyl ether warhead yielded 146-fold selectivity for AKR1C3 over AKR1C1/C2, which was further reduced on PROTAC formation to 116-fold.

Critically we showcase the potential of a PROTAC functionalized AKR1C3 degrader in providing significant degradation of AKR1C3 expression in 22RV1 prostate cancer cells. The PROTAC greatly outperforms small molecule AKR1C3 inhibitors **3** and **4** at equal 10 nM concentrations (Fig. 8a) after 72 h incubation affording significant degradation of AKR1C3 in 22Rv1 cells grown in normal culture media. Gratifyingly, the PROTAC, through its ability to degrade AKR1C3, also brings about concomitant degradation of ARv7 over 72 h (Fig. 7a). A trend in degradation of AKR1C1/C2 is also observed despite the selectivity of the PROTAC to AKR1C3. This phenomenon is also seen with much more selective small molecule AKR1C3 inhibitors. The degradation of AKR1C3 by PROTAC **5** after 4 h is prevented by inhibition of the proteasome with the small molecule inhibitor MG132, providing evidence that **5** induces AKR1C3 degradation by a proteasome-dependent mechanism (Fig. 8c). At 4 h, significant degradation of ARv7 is not observed (Fig. 8d), likely due to temporal effects of AKR1C3 degradation required to disrupt the AKR1C3/ARv7 stabilizing complex. Pre-treatment of 22Rv1 cells with the E3 ligase ligand lenalidomide for 2 h followed by PROTAC for 72 h trends to counter degradation (Fig. 8e, f) but is not significant. While it would be expected that the E3 ligase ligand would compete with the PROTAC for E3 ligase binding and reduce degradation, the intrinsic degradation of AKR1C3 observed with lenalidomide complicates interpretation of this data, as synergistic or additive degradation effect may be obscuring reduced PROTAC effect. Further evidence of the PROTAC mechanism of action is provided by observation of the ‘hook effect’ at high concentrations (Fig. S5).

PROTAC **5** exerts its effects to reduce cell viability of prostate cancer cells in direct correlation to AKR1C3 expression. In 22Rv1 cells grown in normal culture media (moderate AKR1C3 expression) **5** significantly reduces cell viability from 0.01 µM concentration and sensitizes the cells to the cytotoxic action of the clinical agent enzalutamide (Fig. 9b) upon cotreatment. As shown in prior studies, this effect would be expected to be enhanced upon pretreatment of **5** before enzalutamide exposure^7,15^. Warhead compound **4** affords no effect in this cell line despite similar AKR1C3 IC_50_, again suggesting the superior effects of the degrader. 22Rv1 cells when grown in CSS media express AKR1C3 and ARv7 to a much greater extent than when grown in normal media. As expected, PROTAC **5** exhibited much greater activity in these cells, reducing cell viability to a greater extend when AKR1C3 expression is higher. Similarly, cotreatment of ENZ with **5** at just 0.1 µM results in sensitization of this resistant cell line to the actions of the clinical chemotherapeutic (Fig. 9c). AKR1C3 null LNCaP prostate cancer cells show no reduction of cell viability upon treatment of **5** from 0.001 to 10 µM. The LNCaP cells, lacking AKR1C3 and ARv7 expression^7^, are sensitive to the cytotoxicity of ENZ (Fig. 9d). When these cells are stably transfected to overexpress AKR1C3 (Fig. 9e), ENZ resistance is observed. PROTAC **5** affords significant reduction in cell viability at 1 and 10 µM, confirming the target of **5** as AKR1C3.

In conclusion, we report the first PROTAC (**5**) for the targeted degradation of AKR1C3 with a dual effect to degrade ARv7 and AKR1C1/C2. The PROTAC functionalized compound is far more effective than small molecule inhibitors to induce AKR1C3 degradation and does so from 4 h post exposure and this translates to greater effects on cell viability of a variety of AKR1C3 differentially expressing prostate cancer cell lines. PROTAC **5** has been characterized to exert degradation of AKR1C3 in a proteasome dependant manner and possess the ‘hook effect’ intrinsic of PROTACs.

AKR1C3 and ARv7 are highly implicated in the development of chemotherapeutic resistance to clinical ARSIs in advanced prostate cancer. The ability to degrade AKR1C1/1C2 in combination provides a potential strategy for pan-degradation to surmount cancer chemotherapeutic drug resistance, with **5** showing in vitro effect to sensitize AKR1C3 and ARv7 expressing cells to the action of the clinical agent Enzalutamide. Degradation of the AKR1C3/ARv7 axis represents a promising therapeutic strategy to counter drug resistance to ARSIs. With further structure-activity relationship activities defining optimal selection of warhead, linker, and E3 ligase ligand, more potent and selective degraders will likely be identified that can be used as chemical probes and for further development as potential therapeutics, studies currently ongoing in our laboratory.

## Methods

### Chemistry

Full synthetic methods and characterization of intermediates are described in the supplementary methods section of the supplementary material.

*(E)-3-(5-((4-((1-(2-(2-(2-((2-(2,6-dioxopiperidin-3-yl)-1-oxoisoindolin-4-yl)amino)-2-oxoethoxy)ethoxy)ethyl)-1H-1,2,3-triazol-4-yl)methoxy)benzyl)carbamoyl)-[1,1’-biphenyl]-3-yl)acrylic acid (PROTAC* **5**) R_f_ = 0.13 (Hexane/EtOAc = 1:1). ^1^H NMR (400 MHz, CD_3_OD): ***δ***_H_ 2.14–2.48 (4H, m), 3.70 (5H, br. s), 3.95–3.99 (2H, m), 4.10–4.12 (2H, m), 4.54 (2H, s), 4.60–4.63 (2H, m), 4.94–4.98 (2H, m), 5.07 (2H, s), 6.65–6.75 (1H, m), 6.92 (d, *J* = 8.8 Hz, 2H), 7.29 (d, *J* = 7.6 Hz, 2H), 7.41 (t, *J* = 6.8 Hz, 1H), 7.47–7.51 (3H, m), 7.63 (d, *J* = 7.2 Hz, 1H), 7.73 (d, *J* = 7.6 Hz, 3H), 7.79–7.84 (1H, m), 8.01 (1H, s), 8.09 (d, *J* = 5.2 Hz, 1H), 8.12 (d, *J* = 1.6 Hz, 1H), 8.17–8.18 (1H, m).^13^C NMR (150 MHz, CD_3_OD): ***δ***_C_ 15.5, 15.6, 16.7, 24.4, 24.9, 27.9, 28.6, 28.9, 29.5, 29.8, 31.3, 42.3, 45.9, 48.9, 49.4, 53.4, 53.6, 55.4, 55.6, 55.7, 60.4, 68.4, 69.3, 69.5, 70.0, 114.1, 119.5, 119.9, 124.2, 124.5, 126.0, 126.3, 126.3, 126.7, 127.3, 128.1, 128.2, 128.9, 130.8, 131.7, 132.1, 132.2, 134.8, 135.0, 135.1, 139.0, 141.8, 143.1, 143.1, 157.0, 167.3, 168.2, 169.1, 172.6, 174.2, 175.4. ESI-HRMS (m/z): [M + H]^+^ calcd for C_45_H_43_N_7_O_10_, 842.3144; found 842.3143.

### Enzyme inhibition assay

(*S*)-(+)-1,2,3,4-tetrahydro-1-naphthol (*S*-tetralol) was purchased from Sigma-Aldrich (St. Louis, MO). Nicotinamide adenine dinucleotide (NAD^+^) and nicotinamide adenine dinucleotide phosphate (NADP^+^) were purchased from Roche Diagnostics (Indianapolis, IN). Homogeneous recombinant enzymes AKR1C1, AKR1C2, AKR1C3 and AKR1C4 were prepared and purified as previously described^39^. The specific activities of AKR1C1, AKR1C2, and AKR1C3 for the oxidation of *S*-tetralol were 1.4, 2.5 and 3.6 μmol/(min mg), respectively.

### Assay of enzyme activity

The dehydrogenase activities of AKR1C3 and AKR1C2 were determined by measuring the UV absorption of NADH formation at 340 nm using a Beckman DU-640 spectrophotometer. A typical assay solution contained 100 mM potassium phosphate pH 7.0, 2.3 mM NAD^+^, 3.0 mM (*S*)-(+)-1,2,3,4- tetrahydro-1-naphthol (*S*-tetralol), and 4% acetonitrile (v/v). The mixtures were incubated at 37 °C for 3 min followed by adding a serial dilution of AKR1C1, AKR1C2, or AKR1C3 solution to a final volume of 1 mL to initiate the reaction. After continuously monitoring for 5 min, we recorded the increase in UV absorption using different concentrations of an enzyme to calculate the initial velocity and determine the specific activity of the enzyme.

### Enzyme IC_50_ value determination

The inhibitory potency for each compound was represented by IC_50_ values and measured as described before. The IC_50_ values of test compounds were determined by measuring their inhibition of the NADP^ +^ -dependent oxidation of *S*-tetralol catalyzed by AKR1C3 or AKR1C2. The concentrations of *S*-tetralol used in this assay for AKR1C3 and AKR1C2 were 165 μM and 15 μM, respectively, which were equal to the Km value for each enzyme isoform to make a direct comparison of IC50 values. The IC_50_ value of each compound was acquired from a single experiment with each inhibitor concentration run in quadruplicate and directly calculated by fitting the inhibition data to an equation [y = (range)/[1 + (I/IC_50_)S] + background] using Grafit 5.0 software. In this equation, “range” is the fitted uninhibited value minus the “background” and “S” is a slope factor. “I” is the concentration of the inhibitor. The equation assumes that y decreases with the increasing “I”.

### Cell culture and reagents

22Rv1 and LNCaP cells were purchased from the American Type Culture Collection (ATCC in 2016) and cultured in RPMI 1640 supplemented with 10% Fetal Bovine Serum (FBS, Fisher Scientific, MT35011CV) and Penicillin-Streptomycin Solution (1%, Fisher Scientific, MT30001CI) at 37 °C in a humidified incubator with 5% carbon dioxide atmosphere. Where indicated, cells were also cultured in charcoal-stripped (CSS) media prepared by supplementing RPMI 1640 without phenol red with charcoal-stripped FBS. LNCaP1C3 cells overexpressing AKR1C3 were generated by stable transfection of AKR1C3 plasmid as previously described^38^. Stock solutions of E3 ligase ligand, lenalidomide **6** (10 mM), PROTAC **5** (25 mM), Warhead **4** and AKR1C3 inhibitor **3** (100 mM) were prepared in DMSO.

### Cell viability assay

Cells were seeded at a density of 10,000 cells/well in a 96-well plate containing 100 μL growth media per well and were incubated at 37 °C in a humidified incubator with 5% carbon dioxide over ~18–24 h. Cells were treated with **6,** **5,** **3**, ENZ or combination ENZ and **5** serially diluted at the indicated concentrations limiting the final DMSO concentration to less than 0.1% and incubated at 37 °C for 72 h. 10 μL of MTS reagent (CellTiter96®Aqueous One Solution Reagent, Promega, G3580) was added to each well and incubated at the above-mentioned conditions for 4 h. Absorbance was recorded at OD 490 nm on a Synergy LX multi-mode reader and the viability of cells were plotted as percentage of DMSO control.

### Western blot

22Rv1 cells were seeded in a 6-well plate and incubated overnight. Next day, the cells were treated with varying concentrations of **6** (1 and 10 μM) and **5** (0.5, 1, 10, 50, 100, 250, and 500 nM; 1, 10, and 50 μM) for 24 h and **5** (1 and 10 nM) at different time points (0, 2, 4, 6, 12, 16, 24, 48, and 72 h). Whole cell lysates were extracted in RIPA lysis buffer containing protease inhibitor and EDTA (Fisher Scientific, PI78440). Protein concentrations in each sample was estimated following BCA assay (Pierce™ BCA protein assay kit, Fisher Scientific, PI23227) as per the manufacturer’s protocol. Proteins were standardized using RIPA lysis buffer with Laemelli SDS sample buffer (Thermo Scientific, AAJ61337AC) and heated at 100 °C for 15 min. The proteins were resolved on a 4–12% premade gel (Invitrogen, NW04120BOX) at 60–90 V in 20X Bolt™ MES SDS running buffer (Fisher Scientific, B000202) and transferred onto a nitrocellulose membrane (Invitrogen, IB23001) using iBlot 2. The membranes were blocked with 2.5% non-fat dry milk (Bio-Rad, 1706404) in TBST (1X Tris-buffered saline, Bio-Rad 1706435, and 0.1% Tween 20) for 1 h, and the membrane was incubated overnight on a rocking platform at 4 °C with the desired primary antibodies against AKR1C3 (mouse mAb, 1:200, Sigma, A6229), AKR1C1/C2 (rabbit mAb, 1:500, Abcam, ab179448), ARv7 (rabbit mAb, 1:500, Abcam, ab198394) and actin (mouse mAb, 1:1,000, ThermoFisher, MA5-11869). The membranes were washed three times, 10 min each, with TBST on a rocking platform wherein they were incubated with rabbit anti-mouse IgG (ThermoFisher, 31450) or goat anti-rabbit IgG (Jackson ImmunoResearch, 111-035-003) secondary antibody (1:1,000) horseradish peroxidase conjugate for 1 h. The membranes were again washed three times with 1X TBST, upon which they were exposed to LI-COR WesternSure PREMIUM chemiluminescent substrate (Fisher Scientific, 50-489-552) and developed in a dark room with Konica Minolta equipment. Bands were quantified by densitometry using ImageJ software and fold change in AKR1C3, AKR1C1/C2, and ARv7 protein expression was determined based on actin controls.

### Docking studies

Docking experiments were performed with SeeSAR 12.1 software (BioSolveIT, Sankt Augustin, Germany). The crystal structure of AKR1C3 bound with the reference ligand indomethacin (PDB ID: 3UG8) was imported into the binding site tool as a PDB file. The reference ligand was removed, and the binding site defined as a 30 amino acid residue pocket directly surrounding the template ligand. The default parameter settings of SeeSAR were employed. Compounds were prepared and docked with FlexX, wherein fragments are placed into multiple places in the defined pocket and scored with a pre-scoring system^40^. FlexS^41^, was used to generate compound/reference ligand superimpositioning to determine similarity between the test compound and the reference ligand, providing a ranked list for prioritizing compounds. Binding poses were scored by hydrogen dehydration (HYDE)^42^ and the top 20 scoring binding poses of each compound were imported and analyzed in SeeSAR. The top scoring pose was selected based on estimated affinity, ligand efficiency, and torsion energy. Docking figure is generated from a perspective to illustrate binding interactions in the most accessible way in a 2D figure.

### Reporting summary

Further information on research design is available in the Nature Portfolio Reporting Summary linked to this article.

### Supplementary information

Supplementary Information

Description of Additional Supplementary Files

Supplementary Data 1

Reporting Summary

## Data Availability

Supporting material includes experimental details, methods, characterization, Supplementary Figs., ^1^H, and ^13^C NMR spectra. Source data for all graphs is included in Supplementary Data 1. Raw fid files are available on request to the corresponding author.
