# Supplementary material for: Discovery of an Aldo-Keto reductase 1C3 (AKR1C3) degrader
Source: Commun Chem. 2024 Apr 29;7:95. doi: 10.1038/s42004-024-01177-4 (PMC11059152; doi:10.1038/s42004-024-01177-4)
Supplement: Supplementary file 1 — Supplementary Information [file 42004_2024_1177_MOESM1_ESM.pdf]

Discovery of an Aldo-Keto Reductase 1C3 (AKR1C3) Degrader

Angelica V. Carmona,[a,#] Shirisha Jonnalagadda,[a,#] Alfie M. Case,[a] Krishnaiah Maddeboina,[a] Sravan K. Jonnalagadda,[a] Louise F. Dow,[a] Ling Duan,[b] Trevor M. Penning,[b] Paul C. Trippier[a,c,d]

Supplementary Material

[a]Department of Pharmaceutical Sciences, College of Pharmacy, University of Nebraska Medical Center, Omaha, Nebraska 68106, United States

[b]Center of Excellence in Environmental Toxicology, Department of Systems Pharmacology and Translational Therapeutics, University of Pennsylvania, Philadelphia, Pennsylvania 19104, United States

[c]Fred & Pamela Buffett Cancer Center, University of Nebraska Medical Center, Omaha, Nebraska 68106, United States

[d]UNMC Center for Drug Discovery, University of Nebraska Medical Center, Omaha, Nebraska 68106, United States

[#]These authors contributed equally to this work

Contents

Figure S1 3

Figure S2 4

Figure S3 5

Figure S4 6

Figure S5 7

Figure S6 8

Supplementary Methods 9

Experimental Methods 9

Supplementary References 13

^{1}H, ^{13}C NMR Spectra and raw Western blots 14

Figure S1. Western blots and quantification of AKR1C3, AKR1C1/C2 and ARv7 protein expression in 22Rv1 prostate cancer cells treated with A) AKR1C3 inhibitor **3** at 43 nM; B) AKR1C3 inhibitor **3** at 1 μM; C) AKR1C3 inhibitor warhead **4** at 1 μM and D) DMSO; for 0, 24, 48 and 72 hours. Images are representative of at least two technical replicates. Graphs represent mean values ± SEM.

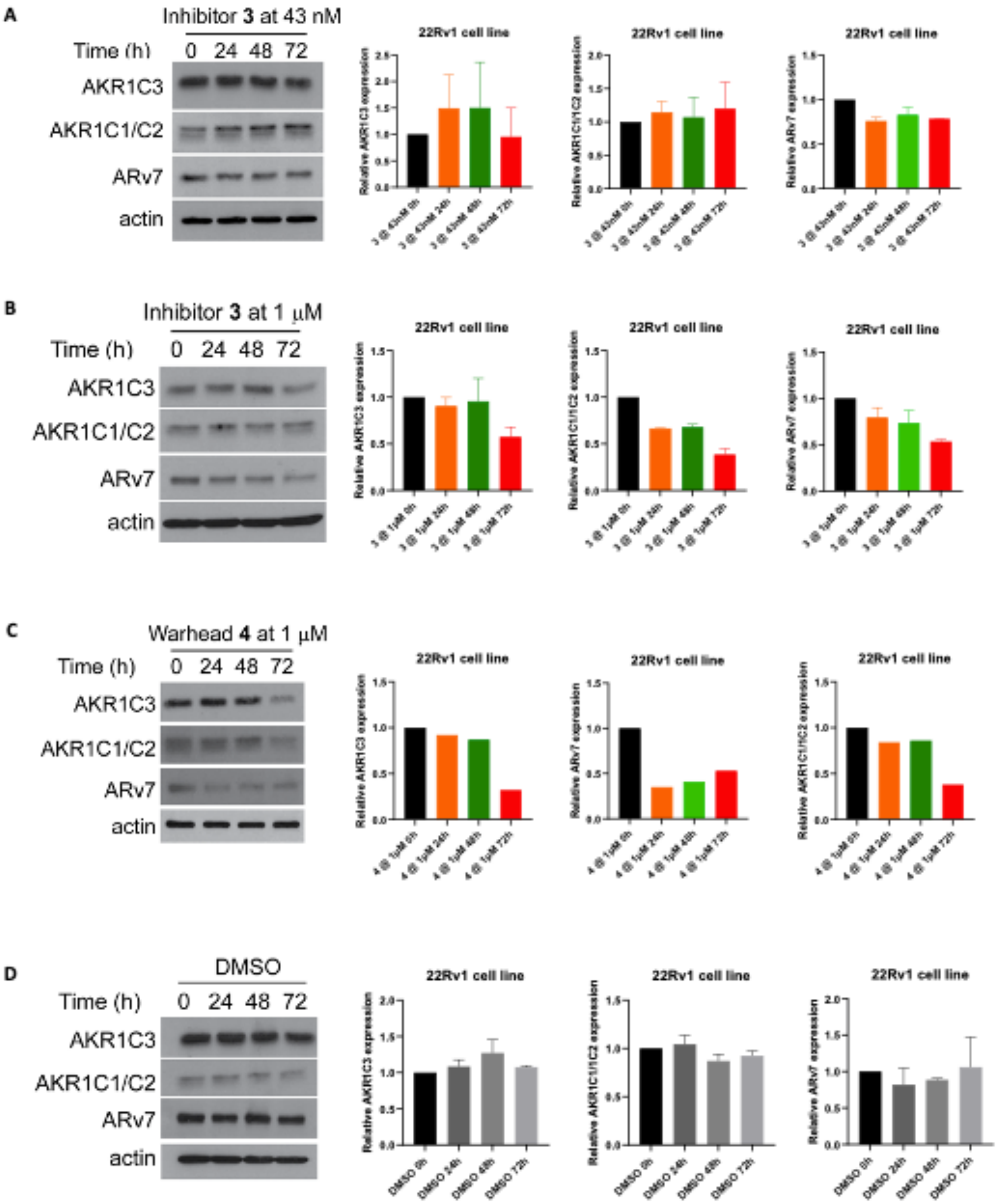

Figure S2. Half-maximal inhibition (IC_{50}) curves versus AKR1C3 for Warhead **4** and PROTAC **5**. Isolated enzyme assay.

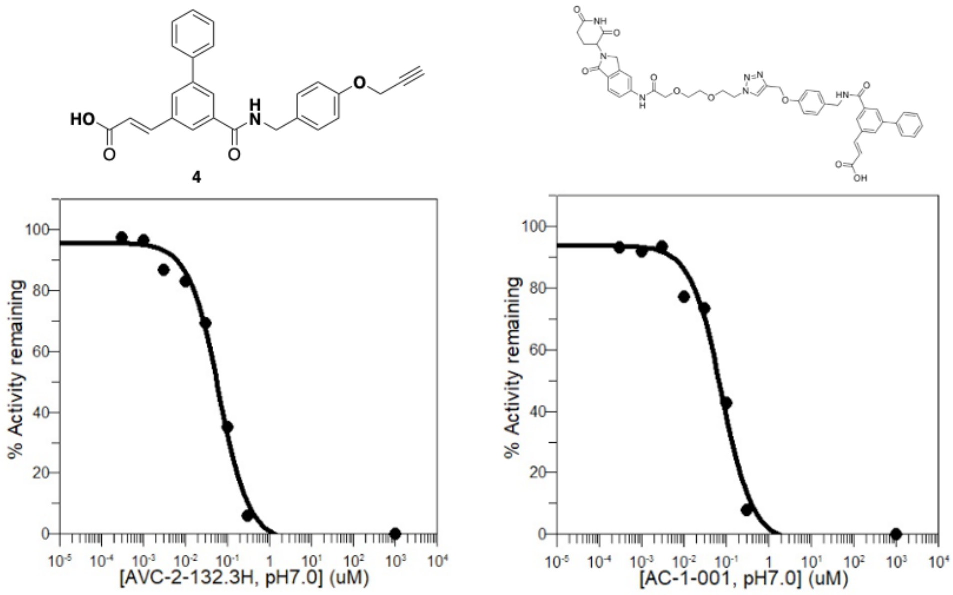

Parameter	Value	Std. Error
Y Range	98.2290	5.3675
IC 50	0.0621	0.0102
Slope factor	1.2018	0.1845
Background	-2.4587	4.4543

Parameter	Value	Std. Error
Y Range	96.3495	7.0962
IC 50	0.0777	0.0173
Slope factor	1.1848	0.2409
Background	-2.5604	5.9762

Figure S3. Activity of selected compounds to ameliorate 22Rv1 prostate cancer cell viability. A) PROTAC **5**, B) AKR1C3 inhibitor **3**, C) E3 ligase ligand lenalidomide **6**, were treated with specified concentrations for 72 h. The IC_{50} was obtained by MTS assay. Data is representative of the mean ± standard deviation of two independent experiments performed in duplicate.

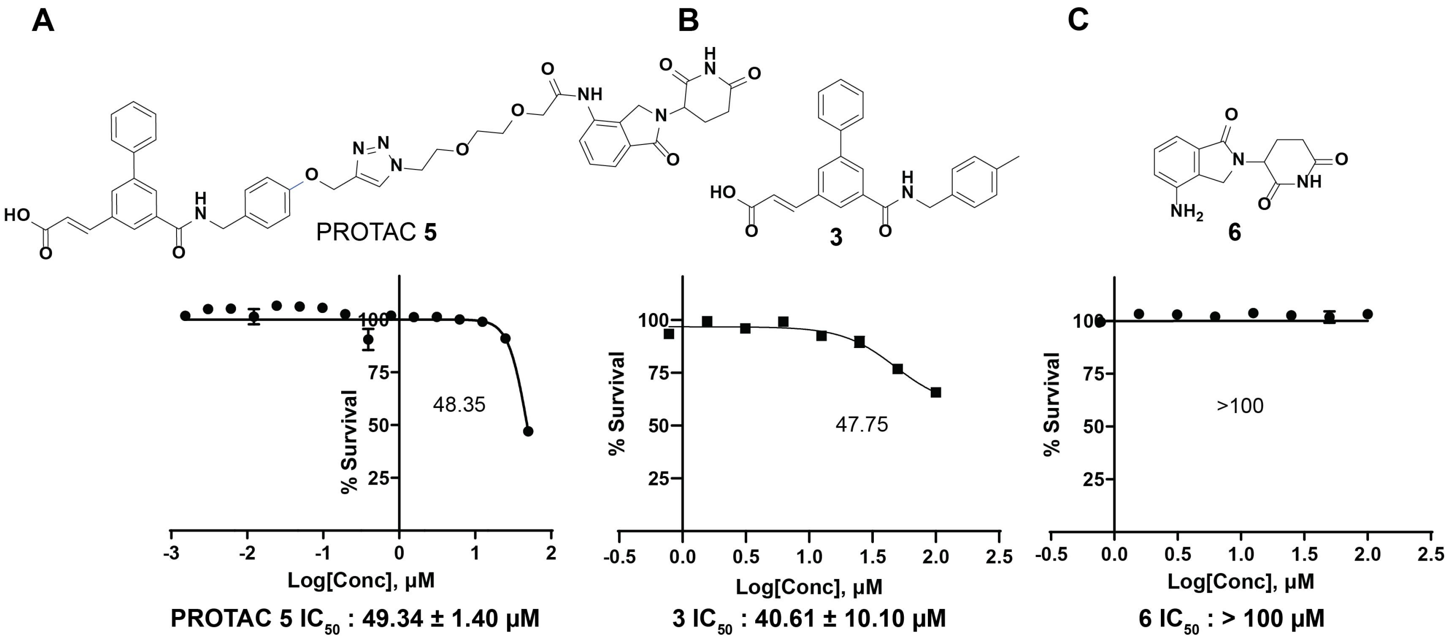

Figure S4. (A) Time study of degradation effect of AKR1C3 upon treatment of PROTAC **5** (1 nM) at different time points (0, 2, 4, 6, 12, 16, 24, 48, and 72 h). Blots are representative of two separate experiments; (B) Quantitative of A for AKR1C3 expression; (C) Combined quantification of AKR1C3 expression from n=2 experiments; Data obtained from at least two biological independent experiments are depicted as mean ± SEM.

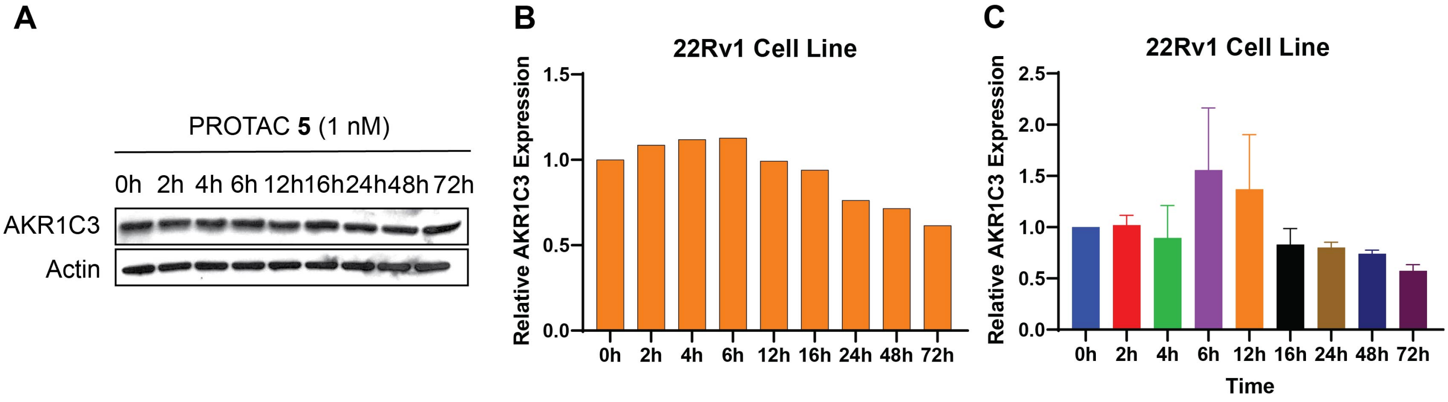

Figure S5 Western blot analyses of AKR1C3 degradation upon the treatment of lenalidomide **6** (10 μM) and degrader **5** at 1, 10 and 50 μM concentrations for 24 h (A); (B) Quantitative analyses of the relative AKR1C3 protein levels at 1, 10, and 50 μM of A; (C) Quantification of the relative AKR1C3 protein levels at 1, 10, and 50 μM, data was obtained from two independent experiments and shown as mean ± standard deviation.

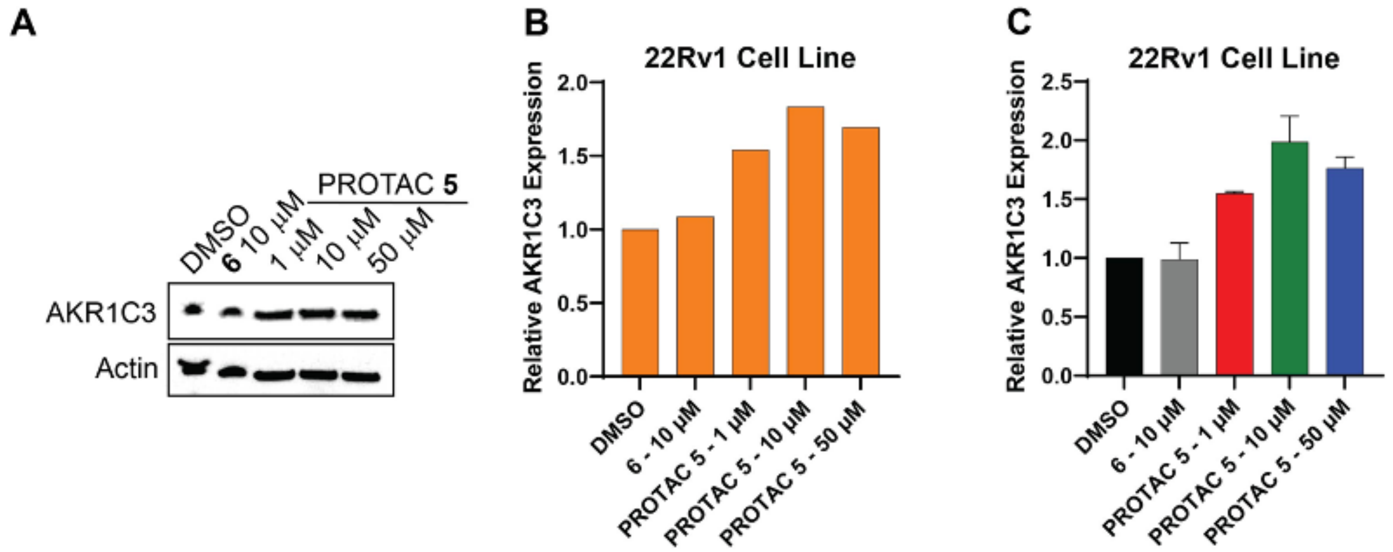

Figure S6. Half-maximal degradation (DC_{50}) curves versus AKR1C3, AKR1C2/3 and ARv7 for PROTAC **5** in 22Rv1 cells after 72 hour treatment.

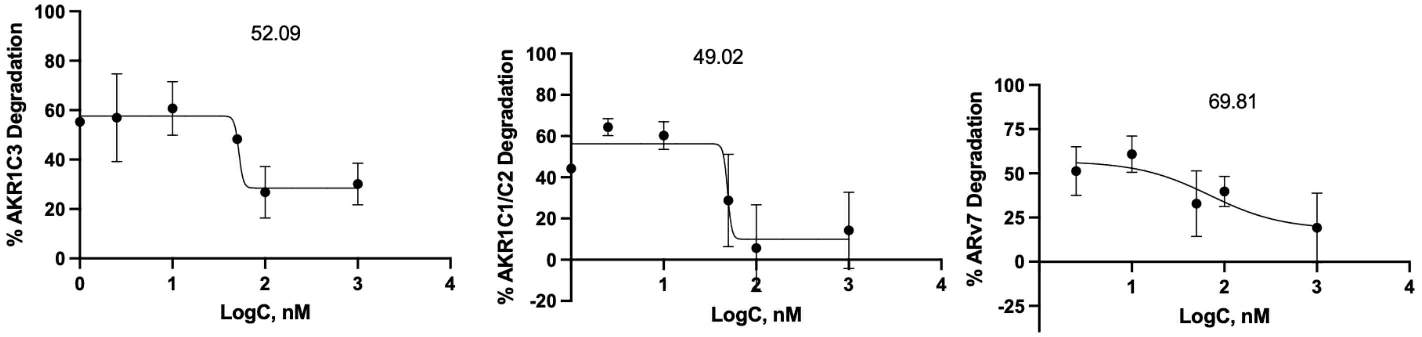

Supplementary Methods

Experimental Methods

General chemistry procedures

All reactions were carried out in oven-dried glassware under positive nitrogen pressure unless otherwise noted. Reaction progress was monitored by thin-layer chromatography carried out on silica gel plates (2.5 cm × 7.5 cm, 200 μm thick, 60 F254) and visualized by using UV (254 nm) or by dragendorff solution as indicator. Flash column chromatography was performed with silica gel (40−63 μm, 60 Å) using the mobile phase indicated. Commercial grade solvents and reagents were purchased from Fisher Scientific (Houston, TX) or Sigma-Aldrich (Milwaukee, WI) and were used without further purification. Anhydrous solvents were purchased from Across Organics and stored under an atmosphere of dry nitrogen over molecular sieves.

^{1}H and ^{13}C NMR spectra were recorded in the indicated solvent on a Bruker 400 MHz Advance III HD spectrometer at 400 and 100 MHz for ^{1}H and ^{13}C, respectively. Multiplicities are indicated by s (single), d (doublet), t (triplet), m (multiplet), and br (broad). Chemical shifts (δ) are reported in parts per million (ppm) and coupling constants (J), in hertz.

High-resolution mass spectra (HRMS) were recorded with an Agilent 6230 LC/TOF spectrometer using an ESI source coupled to an Agilent Infinity 1260 system running in reverse phase with a ZORBAX RRHT Extend-C18 (80 Å, 2.1 x 50 mm, 1.8 μm) column using solvent A (water with 0.1 % Formic acid), solvent B (acetonitrile with 0.1 % Formic acid), and a flow rate of 0.6 mL/min starting a mixture of 95% A and 5% B. Solvent B is gradually increased to 95% at 5 min, held at 95% until 6 min, then gradually ramped back down to 5% at 8.0 min. The purity analysis of final compounds was determined ≥95% pure using a Waters ACQUITY ultra-performance liquid chromatography (UPLC) H-Class System with TUV (254 nm) detector and Empower 2 software (Milford, MA, USA) using an Agilent Eclipse plus C18 5μ column (4.6 X 150 mm). Chromatography was performed using solvent A (water with 0.1 % Trifluoroacetic acid), solvent B (methanol with 0.1 % Trifluoroacetic acid), and a flow rate of 1.0 mL/min for 20 min. with an isocratic system (20:80, A:B)

(4-((*tert*-butyldiphenylsilyl)oxy)phenyl)methanamine (**8**). To a stirred solution of 4-(aminomethyl)phenol (**7**) (1.85 g, 15.0 mmol, 1 equiv) in anhydrous tetrahydrofuran (75 mL) imidazole (2.04 g, 30.0 mmol, 2 equiv) was added followed by the addition of *tert*-butylchlorodiphenylsilane (TBDPSiCl) (5.8 mL, 22.5 mmol, 1.5 equiv). The mixture stirred overnight at room temperature under a N_2 atmosphere. Water was added and the solution was extracted with DCM. The organic layer was collected, dried over anhydrous Na_2SO_4, filtered, and concentrated in vacuo. The crude product was purified by flash column chromatography using hexane/EtOAc as the eluents to afford the titled compound as a yellow solid (3.09 g, 57%). R_f = 0.07 (hexane/EtOAc = 9:1). ^{1}H NMR (400 MHz, $CDCl_3$): δ_H 1.15 (9H, s), 1.86 (2H, s), 3.75 (2H, s), 6.77 (d, J = 8.0 Hz, 2H), 7.05 (d, J = 8.0 Hz, 2H) 7.38-7.46 (6 H, m), 7.76 (d, J = 8.0 Hz, 4H). ^{13}C NMR (100 MHz, $CDCl_3$): δ_C 19.5, 26.6, 45.8, 119.7, 127.8, 128.1, 129.9, 133.0, 135.5, 135.5, 154.5.

3-bromo-*N*-(4-((*tert*-butyldiphenylsilyl)oxy)benzyl)-5-iodobenzamide (**10**). To a stirred solution of 3-bromo-5-iodobenzoic acid (**9**) (0.72 g, 2.2 mmol, 1 equiv) in anhydrous DCM (12 mL) was added EDC·HCl (0.51 g, 2.7 mmol, 1.2 equiv) and HOBt hydrate (0.44 g, 2.7 mmol, 1.2 equiv) at 0 ºC under a N_2 atmosphere. At room temperature DIPEA (0.86 g, 6.6 mmol, 3 equiv) and **8** (0.80 g, 2.2 mmol, 1 equiv) was added, the mixture stirred overnight at room temperature under a N_2 atmosphere. The reaction mixture was washed with a saturated solution of NH_4Cl, water, and extracted with DCM. The organic layer was collected, dried over anhydrous Na_2SO_4, filtered, and concentrated in vacuo to afford the titled compound as a tan solid (1.29 g, 87%). R_f = 0.43 (hexane/EtOAc = 6:1). ^{1}H NMR (400 MHz, $CDCl_3$): δ_H 1.13 (9H, s), 4.46 (d, J = 4.0 Hz, 2H), 6.41 (t, J = 6.0 Hz, 1H), 6.75 (d, J = 12.0 Hz, 2H), 7.05 (d, J = 12.0 Hz, 2H), 7.37-7.47 (7H, m), 7.73 (d, J = 8.0 Hz, 4H), 7.85 (1H, s), 7.96 (d,

J= 16.0 Hz, 2H). ^{13}C NMR (100 MHz, $CDCl_3$): δ_C 19.5, 26.5, 43.9, 94.5, 120.0, 123.2, 127.8, 129.1, 129.9, 130.1, 132.6, 134.7, 135.5, 137.8, 142.4, 155.3, 164.3.

Methyl (*E*)-3-(3-bromo-5-((4-((*tert*- butyldiphenylsilyl)oxy)benzyl)carbamoyl)phenyl)acrylate (**11**) To a stirred solution of (**10**) (2.1 g, 3.1 mmol, 1 equiv) in anhydrous toluene (42 mL) was added methyl acrylate (0.42 mL, 4.7 mmol, 1.5 equiv), $P(Ph)_3$ (0.08 g, 0.3 mmol, 0.1 equiv), NEt_3 (1.31 mL, 9.4 mmol, 3 equiv) and $Pd(OAc)_2$ (0.07 g, 0.3 mmol, 0.1 equiv) and the reaction refluxed overnight under a N_2 atmosphere. While the reaction mixture was still hot, the contents were filtered. The filtrate was then allowed to cool and was filtered through a celite® pad with DCM. The reaction mixture was washed with a saturated solution of NH_4Cl, water, and extracted with DCM. The organic layer was collected, dried over anhydrous Na_2SO_4, filtered, and concentrated in vacuo. The crude product was purified by flash column chromatography using DCM/MeOH as the eluents to afford the titled compound as a brown semi-solid (0.60 g, 30%). R_f = 0.1 (DCM/MeOH = 1:0). ^1H NMR (400 MHz, $CDCl_3$): δ_H 1.10 (9H, s), 3.80 (3H, s), 4.47 (d, *J* = 4.0 Hz, 2H), 6.36 (1H, br. s), 6.43 (d, *J* = 16.0 Hz, 1H), 6.73 (d, *J* = 8.0 Hz, 2H), 7.05 (d, *J* = 8.0 Hz, 2H), 7.35-7.44 (6H, m), 7.56 (d, *J* = 16.0 Hz, 1H), 7.70 (d, *J* = 8.0 Hz, 5H), 7.80 (d, *J* = 12.0 Hz, 1H). ^{13}C NMR (100 MHz, $CDCl_3$): δ_C 19.5, 26.5, 43.9, 52.0, 120.0, 120.5, 123.2, 125.3, 127.8, 129.1, 129.9, 130.0, 131.3, 132.7, 133.3, 135.5, 136.8, 137.0, 142.1, 155.3, 165.2, 166.7.

methyl (*E*)-3-(5-((4-((*tert*-butyldiphenylsilyl)oxy)benzyl)carbamoyl)-[1,1'-biphenyl]-3-yl)acrylate (**12**) To a stirred solution of (**11**) (0.8 g, 1.3 mmol, 1 equiv) in anhydrous toluene (45 mL) was added phenyl boronic acid (0.24 g, 2.0 mmol, 1.5 equiv), $Pd(dppf)Cl_2{\cdot}CH_2Cl_2$ (0.11 g, 0.13 mmol, 0.1 equiv), and Cs_2CO_3 (0.85 g, 2.6 mmol, 2 equiv) and the reaction refluxed overnight under a N_2 atmosphere. While the reaction mixture was still hot, the contents were filtered, and the filtrate was concentrated in vacuo. The concentrated filtrate was washed with hexane (5x), dissolved in diethyl ether, concentrated in vacuo, washed again with hexane (2x), and concentrated in vacuo. The crude product was purified by flash column chromatography using DCM/MeOH as the eluents to afford the titled compound as a white solid (0.16 g, 18%). R_f = 0.30 (DCM/MeOH = 1:0). ^1H NMR (400 MHz, $CDCl_3$): δ_H 1.12 (9H, s), 3.84 (3H, s), 4.54 (d, *J* = 8.0 Hz, 2H), 6.40 (t, *J* = 6.0 Hz, 1H), 6.54 (d, *J* = 16.0 Hz, 1H), 6.76 (d, *J* = 8.0 Hz, 2H), 7.10 (d, *J* = 8.0 Hz, 2H), 7.36-7.50 (9H, m), 7.59 (d, *J* = 8.0 Hz, 2H), 7.73 (d, *J* = 4.0 Hz, 5H), 7.78 (d, *J* = 20.0 Hz, 2H), 7.88 (1H, s), 7.96 (1H, s). ^{13}C NMR (100 MHz, $CDCl_3$): δ_C 19.5, 26.5, 43.8, 51.9, 119.4, 120.0, 125.1, 127.2, 127.3, 127.8, 128.2, 129.0, 129.1, 129.5, 130.0, 130.2, 132.8, 135.4, 135.5, 135.9, 139.5, 142.5, 143.7, 155.3, 166.6, 167.1.

methyl (*E*)-3-(5-((4-hydroxybenzyl)carbamoyl)-[1,1'-biphenyl]-3-yl)acrylate (**13**) To a stirred solution of (**12**) (0.23 g, 0.37 mmol, 1 equiv) in anhydrous THF (5 mL) was added TBAF (1M solution in THF, 0.16 mL, 0.55 mmol, 1.50 equiv) at 0 ºC. The reaction was stirred for 40 minutes at 0 ºC. Water was added and the solution was extracted with EtOAc and washed with brine. The organic layer was collected, dried over anhydrous Na_2SO_4, filtered, and concentrated in vacuo. The residue was washed with hexane/EtoAc (50:1, 40:1) resulting in solid precipitation. The solid was left in hexane (15 mL) overnight. The solid was then washed with hexane/EtOAc (2x 1:0, 3x 1:1). The overall yield of the titled compound as a white solid was 60% (0.1 g). R_f = 0.46 (hexane/EtOAc = 1:1). ^1H NMR (400 MHz, $(CD_3)_2SO$): δ_H 3.75 (s, 3H), 4.43 (d, *J* = 4.0 Hz, 2H), 6.73 (d, *J* = 8.0 Hz, 2H), 6.83 (d, *J* = 16.0 Hz, 1H), 7.17 (d, *J* = 8.0 Hz, 2H), 7.41 (t, *J* = 6.0 Hz, 1H), 7.49 (t, *J* = 8.0 Hz, 2H), 7.78 (t, *J* = 8.0 Hz, 3H), 8.17 (s, 1H), 8.21 (s,2H), 9.10 (t, *J* = 6.0 Hz, 1H), 9.34 (s, 1H). ^{13}C NMR (100 MHz, $(CD_3)_2SO$): δ_C 42.8, 52.1, 115.5, 119.7, 126.0, 127.4, 127.7, 128.6, 129.2, 129.5, 129.8, 130.0, 135.4, 136.1, 139.3, 141.4, 144.3, 156.8, 165.8, 167.1.

methyl(*E*)-3-(5-((4-(prop-2-yn-1-yloxy)benzyl)carbamoyl)-[1,1'-biphenyl]-3-yl)acrylate (**14**)
To a stirred solution of **13** (0.23 g, 0.59 mmol, 1 equiv), in anhydrous DMF was added Cs_2CO_3 (0.25 g, 0.78 mmol, 1.31 equiv) and the mixture refluxed under a N_2 atmosphere. After 10 min, propargyl bromide (0.04 mL, 0.41 mmol, 0.69 equiv) was added. The reaction mixture refluxed for 6 h. The

reaction mixture was filtered, water was added, and the reaction mixture was extracted with EtOAc. The organic layer was collected, dried over anhydrous Na_2SO_4, filtered, and concentrated in vacuo. The residue was then extracted with diethyl ether and washed with water. The organic layer was collected, dried over anhydrous Na_2SO_4, filtered, and concentrated in vacuo and left to dry at room temperature overnight. The residual DMF was removed by washing with copious amounts of water in toluene, dried over anhydrous Na_2SO_4, filtered, and concentrated in vacuo to afford the titled compound as a white solid (0.19 g, 75%). R_f = 0.75 (hexane/EtOAc = 1:1)
^{1}H NMR (400 MHz, $CDCl_3$): δ_H 2.53 (t, J = 2.4 Hz, 1H), 3.82 (3H, s), 4.61 (d, J = 8.0 Hz, 2H), 4.69 (d, J = 4.0 Hz, 2H), 6.53 (d, J = 16.0 Hz, 1H), 6.65 (t, J = 4.0 Hz, 1H), 6.97 (d, J = 8.0 Hz, 2H), 7.32 (d, J = 8.0 Hz, 2H), 7.39-7.43 (m, 1H), 7.45-7.49 (m, 2H), 7.59 (d, J = 4.0 Hz, 2H), 7.73 (d, J = 16.0 Hz, 1H), 7.82 (t, J = 1.4 Hz, 1H), 7.90 (t, J = 1.4 Hz, 1H), 7.99 (t, J = 1.6 Hz, 1H). ^{13}C NMR (100 MHz, $CDCl_3$): δ_C 43.8, 51.9, 55.9, 75.6, 78.5, 115.2, 119.4, 125.1, 127.2, 127.4, 128.2, 129.0, 129.4, 129.6, 131.0, 135.4, 135.8, 139.5, 142.5, 143.7, 157.1, 166.7, 167.1.

(*E*)-3-(5-((4-Methylbenzyl)carbamoyl)-[1,1'-biphenyl]-3-yl)acrylic acid **(3)** The titled compound was afforded as a white solid (68 mg, 71%). ^{1}H NMR (400 MHz, $(CD_3)_2SO$): δ_H 2.29 (3H, s), 4.49 (d, J = 5.6 Hz, 2H), 6.74 (d, J = 16.0 Hz, 1H), 7.15 (d, J = 8.0 Hz, 2H), 7.25 (d, J = 8.0 Hz, 2H), 7.43 (t, J = 7.2 Hz, 1H), 7.52 (t, J = 7.6 Hz, 2H), 7.72 (d, J = 16.0 Hz, 1H), 7.81 (d, J = 7.2 Hz, 2H), 8.15 (1H, s), 8.20 (2H, s), 9.18 (t, J = 5.6 Hz, 1H). ^{13}C NMR (100 MHz, $(CD_3)_2SO$): δ_C 21.14, 42.99, 121.45, 125.79, 127.46, 127.81, 128.53, 129.34, 129.46, 129.76, 135.74, 135.95, 136.35, 136.88, 137.03, 139.40, 141.43, 143.45, 165.90, 168.01. ESI-HRMS (*m/z*): $[M+H]^+$ calcd for $C_{24}H_{22}NO_3$, 372.1594; found, 372.1595.

tert-butyl 2-(2-(2-azidoethoxy)ethoxy)acetate (**16**) Following a modified literature procedure[1]: Under a N_2 atmosphere, to a stirred solution of NaH (60% dispersion in mineral oil, 0.59 g, 14.87 mmol, 1.51 equiv) in anhydrous THF (35 mL) was added a stirred solution of 2-(2-azidoethoxy)ethanol (**15**) (1.29 g, 9.85 mmol, 1 equiv) in anhydrous THF (69 mL) at 0 ºC. After the reaction mixture stirred for 30 min at 0 ºC, *tert*-butyl bromoacetate (2.88 mL, 19.69 mmol, 2 equiv) was added. The mixture stirred for 24 hr at room temperature under a N_2 atmosphere. The reaction mixture was quenched with water (350 mL) and when the effervescence ceased the solution was extracted with EtOAc. The organic layer was collected, dried over anhydrous Na_2SO_4, filtered, and concentrated in vacuo. The crude product was purified by flash column chromatography using Toluene/EtOAc as the eluents to afford the titled compound as a yellow oil (0.72 g, 30%). R_f = 0.29 (Toluene/EtOAc = 1:0). ^{1}H NMR (400 MHz, $CDCl_3$): δ_H 1.48 (9H, s), 3.39 (t, J = 6.0 Hz, 2H), 3.68-3.75 (6H, m), 4.04 (2H, s). ^{13}C NMR (100 MHz, $CDCl_3$): δ_C 28.1, 50.7, 69.1, 70.0, 70.7, 70.8, 81.6, 169.6.

Following a modified literature procedure[2]: To a stirred solution of **16** (0.26 g, 1.06 mmol, 1 equiv) in DCM (0.51 mL) was added TFA (0.25 mL) and the reaction stirred at room temperature for 2 h under a N_2 atmosphere. The reaction mixture was then concentrated in vacuo. At 0º C, the reaction mixture was diluted with DCM (1.30 mL) followed by dropwise addition of $SOCl_2$. The reaction stirred at room temperature for 2 h under a N_2 atmosphere. The solvent was concentrated in vacuo to afford **17** (dark brown oil) which was used in the following steps without purification.

2-(2-(2-azidoethoxy)ethoxy)-*N*-(2-(2,6-dioxopiperidin-3-yl)-1-oxoisoindolin-4-yl)acetamide (**18**) To solution **17** was added NMP (2.57 mL) and the cereblon ligand (lenalidomide) (0.16 g, 0.63 mmol, 0.5 equiv). The reaction mixture stirred at room temperature overnight under a N_2 atmosphere. Water was added and the solution was extracted with EtOAc and washed with brine. The organic layer was collected, dried over anhydrous Na_2SO_4, filtered, and concentrated in vacuo. The crude product was purified by flash column chromatography using DCM/MeOH as the eluents to afford the titled compound as an off white solid (0.18 g, 37%). R_f = 0.47 (DCM/MeOH = 14:1). ^{1}H NMR (400 MHz, $CDCl_3$): δ_H 2.18-2.22 (1H, m), 2.35-2.40 (1H, m), 2.81-2.86 (2H, m), 3.38 (t, J = 4.0 Hz, 2H), 3.71-3.76 (4H, m), 3.81-3.83 (2H, m), 4.19 (2H, s), 4.46 (2H, s), 5.20-5.25 (1H, m), 7.49 (t, J = 16.0 Hz, 1H), 7.67 (d, J = 8.0

Hz, 1H), 7.76 (d, *J* = 8.0 Hz, 1H), 8.52 (1H, s), 8.71 (1H, s). ^{13}C NMR (100 MHz, $CDCl_3$): δ_C 23.3, 31.5, 46.5, 50.5, 51.9, 70.1, 70.1, 70.5, 71.1, 121.6, 126.3, 129.2, 131.8, 132.9, 134.7, 168.1, 168.9, 169.7, 171.4.

methyl (*E*)-3-(5-((4-((1-(2-(2-(2-((2-(2,6-dioxopiperidin-3-yl)-1-oxoisoindolin-4-yl)amino)-2-oxoethoxy)ethoxy)ethyl)-1*H*-1,2,3-triazol-4-yl)methoxy)benzyl)carbamoyl)-[1,1'-biphenyl]-3-yl)acrylate (**19**) **14** (0.150 g, 0.35 mmol, 1 equiv) and **18** (0.150 g, 0.35 mmol, 1 equiv) were dissolved in a solution of DCM (2.61 mL), MeOH (2.61 mL), and water (1.31 mL). To which $CuSO_4{\cdot}5H_2O$ (8.8 mg, 0.03 mmol, 0.1 equiv) dissolved in water (6.53 mL) was added and the reaction mixture stirred under a N_2 atmosphere for 5 min at room temperature. Sodium ascorbate (28.5 mg, 0.14 mmol, 0.41 equiv) was dissolved in water (6.53 mL) and added to the reaction mixture. The reaction stirred overnight at room temperature under a N_2 atmosphere. Water was added (13 mL) and the solution was extracted with EtOAc. The organic layer was collected, dried over anhydrous Na_2SO_4, filtered, and concentrated in vacuo. The crude product was purified by flash column chromatography using DCM/MeOH as the eluents to afford the titled compound as a white solid (47.9 mgs, 16%). R_f = 0.25 (DCM/MeOH = 20:1). ^{1}H NMR (400 MHz, CD_3OD): δ_H 1.31 (1H, s), 2.08-2.14 (1H, m), 2.33-2.44 (1H, m), 2.71-2.89 (2H, m), 3.69 (4H, s), 3.81 (d, *J* = 4.0 Hz, 3H), 3.94 (t, *J* = 8.0 Hz, 2H), 4.10 (d, *J* = 4.0 Hz, 2H), 4.44 (d, *J* = 4.0 Hz, 2H), 4.50 (d, *J* = 8.0 Hz, 1H), 4.54 (1H, s), 4.59-4.61 (4H, m), 5.06 (2H, s), 5.09-5.18 (2H, m), 6.69 (d, *J* = 16.0 Hz, 1H), 6.90 (d, *J* = 8.0 Hz, 2H), 7.27 (d, *J* = 8.0 Hz, 2H), 7.39 (t, *J* = 6.0 Hz, 1H), 7.47 (t, *J* = 16.0 Hz, 2H), 7.62-7.73 (4H, m), 7.80 (d, *J* = 16.0 Hz, 1H), 8.00 (1H, s), 8.06 (1H, s), 8.11 (1H, s), 8.17 (1H, s). ^{13}C NMR (100 MHz, CD_3OD): δ_C 22.7, 30.9, 42.7, 49.8, 50.9, 52.2, 60.9, 68.9, 69.7, 69.9, 70.5, 114.5, 118.9, 120.5, 124.7, 125.0, 126.7, 126.8, 127.3, 127.8, 128.7, 128.7, 129.4, 131.3, 132.2, 132.6, 135.3, 135.4, 135.5, 139.4, 142.3, 143.5, 143.8, 157.4, 167.4, 167.7, 169.5, 169.5, 170.6, 173.2.

(*E*)-3-(5-((4-((1-(2-(2-(2-((2-(2,6-dioxopiperidin-3-yl)-1-oxoisoindolin-4-yl)amino)-2-oxoethoxy)ethoxy)ethyl)-1*H*-1,2,3-triazol-4-yl)methoxy)benzyl)carbamoyl)-[1,1'-biphenyl]-3-yl)acrylic acid (**5**) To a stirred solution of **19** (41 mg, 0.05 mmol, 1 equiv) in a mixture of THF/MeOH (1:1) (2 mL) was added aqueous 1N NaOH (5 mg, 0.14 mmol, 3 equiv) solution. The mixture was stirred at 56 ºC for 2 hours. The solvent was concentrated in vacuo, and pH was adjusted to 2-5 with 1 N HCl solution. The solution was extracted with EtOAc. The organic layer was collected, dried over anhydrous Na_2SO_4, filtered, and concentrated in vacuo. The precipitate was air-dried to afford the titled compound as a colorless semi-solid (19.3 mgs, 48%). R_f = 0.13 (Hexane/EtOAc = 1:1). ^{1}H NMR (400 MHz, CD_3OD): $\boldsymbol{\delta}_H$ 2.14-2.48 (4H, m), 3.70 (5H, br. s), 3.95-3.99 (2H, m), 4.10-4.12 (2H, m), 4.54 (2H, s), 4.60-4.63 (2H, m), 4.94-4.98 (2H, m), 5.07 (2H, s), 6.65-6.75 (1H, m), 6.92 (d, *J* = 8.8 Hz, 2H), 7.29 (d, *J* = 7.6 Hz, 2H), 7.41 (t, *J* = 6.8 Hz, 1H), 7.47-7.51 (3H, m), 7.63 (d, *J* = 7.2 Hz, 1H), 7.73 (d, *J* = 7.6 Hz, 3H), 7.79-7.84 (1H, m), 8.01 (1H, s), 8.09 (d, *J* = 5.2 Hz, 1H), 8.12 (d, *J* = 1.6 Hz, 1H), 8.17-8.18 (1H, m).^{13}C NMR (150 MHz, CD_3OD): $\boldsymbol{\delta}_C$ 15.5,15.6,16.7, 24.4, 24.9, 27.9, 28.6, 28.9, 29.5, 29.8, 31.3, 42.3, 45.9, 48.9, 49.4, 53.4, 53.6, 55.4, 55.6, 55.7, 60.4, 68.4, 69.3, 69.5, 70.0, 114.1, 119.5, 119.9, 124.2, 124.5, 126.0, 126.3, 126.3, 126.7, 127.3, 128.1, 128.2, 128.9, 130.8, 131.7, 132.1, 132.2, 134.8, 135.0, 135.1, 139.0, 141.8, 143.1, 143.1, 157.0, 167.3, 168.2, 169.1, 172.6, 174.2, 175.4. ESI-HRMS (m/z): $[M + H]^+$ calcd for $C_{45}H_{43}N_7O_{10}$, 842.3144; found 842.3143.

Supplementary References:

1. Wan, Z.; Li, Y.; Bo, S.; Gao, M.; Wang, X.; Zeng, K.; Tao, X.; Li, X.; Yang, Z.; Jiang, Z.-X. Amide bond-containing monodisperse polyethylene glycols beyond 10 000 Da. *Org. Biomol. Chem.* **2016,** *14*, 7912.

2. Zhang, F.; Wu, Z.; Chen, P.; Zhang, J.; Wang, T.; Zhou, J.; Zhang, H. Discovery of a new class of PROTAC BRD4 degraders based on a dihydroquinazolinone derivative and lenalidomide/pomalidomide. *Biorg. Med. Chem.* **2020,** *28*, 115228.

^{1}H NMR (400 MHz, $CDCl_3$), (4-((*tert*-butyldiphenylsilyl)oxy)phenyl)methanamine **(8)**

AVC-74
CDCl3

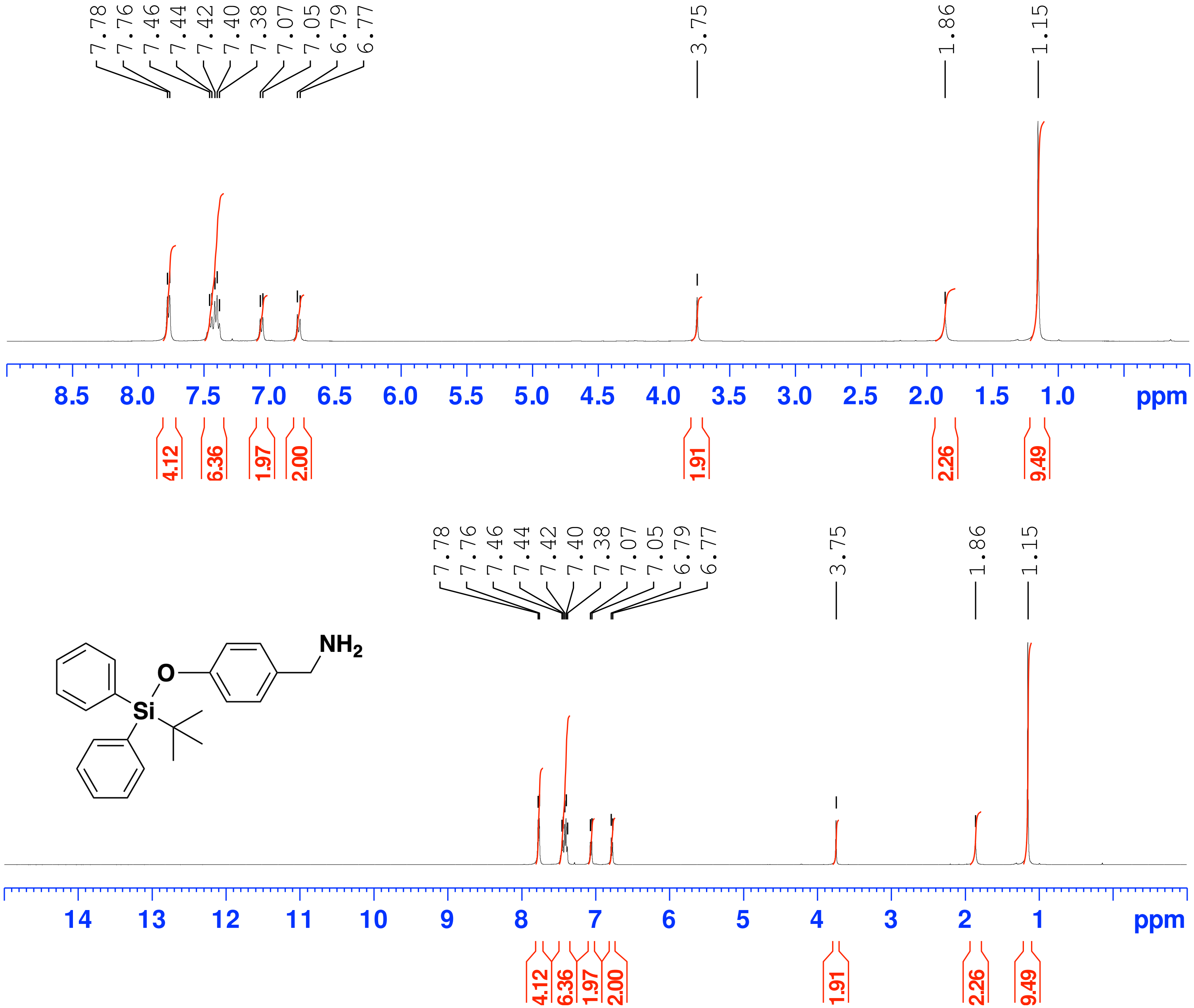

BRUKER

Current Data Parameters
NAME Oct04-2021-PTlab
EXPNO 10
PROCNO 1

F2 - Acquisition Parameters
Date_ 20211004
Time 14.14
INSTRUM spect
PROBHD 5 mm PABBO BB/
PULPROG zg30
TD 30046
SOLVENT CDCl3
NS 16
DS 1
SWH 8012.820 Hz
FIDRES 0.266685 Hz
AQ 1.8748704 sec
RG 57
DW 62.400 usec
DE 6.50 usec
TE 300.1 K
D1 6.00000000 sec
TD0 1

======== CHANNEL f1 ========
SFO1 400.2821952 MHz
NUC1 1H
P1 10.86 usec
PLW1 17.00000000 W

F2 - Processing parameters
SI 131072
SF 400.2800000 MHz
WDW EM
SSB 0
LB 0.20 Hz
GB 0
PC 1.00

^{13}C NMR (100 MHz, $CDCl_3$), (4-((*tert*-butyldiphenylsilyl)oxy)phenyl)methanamine **(8)**

AVC-74
CDCl3

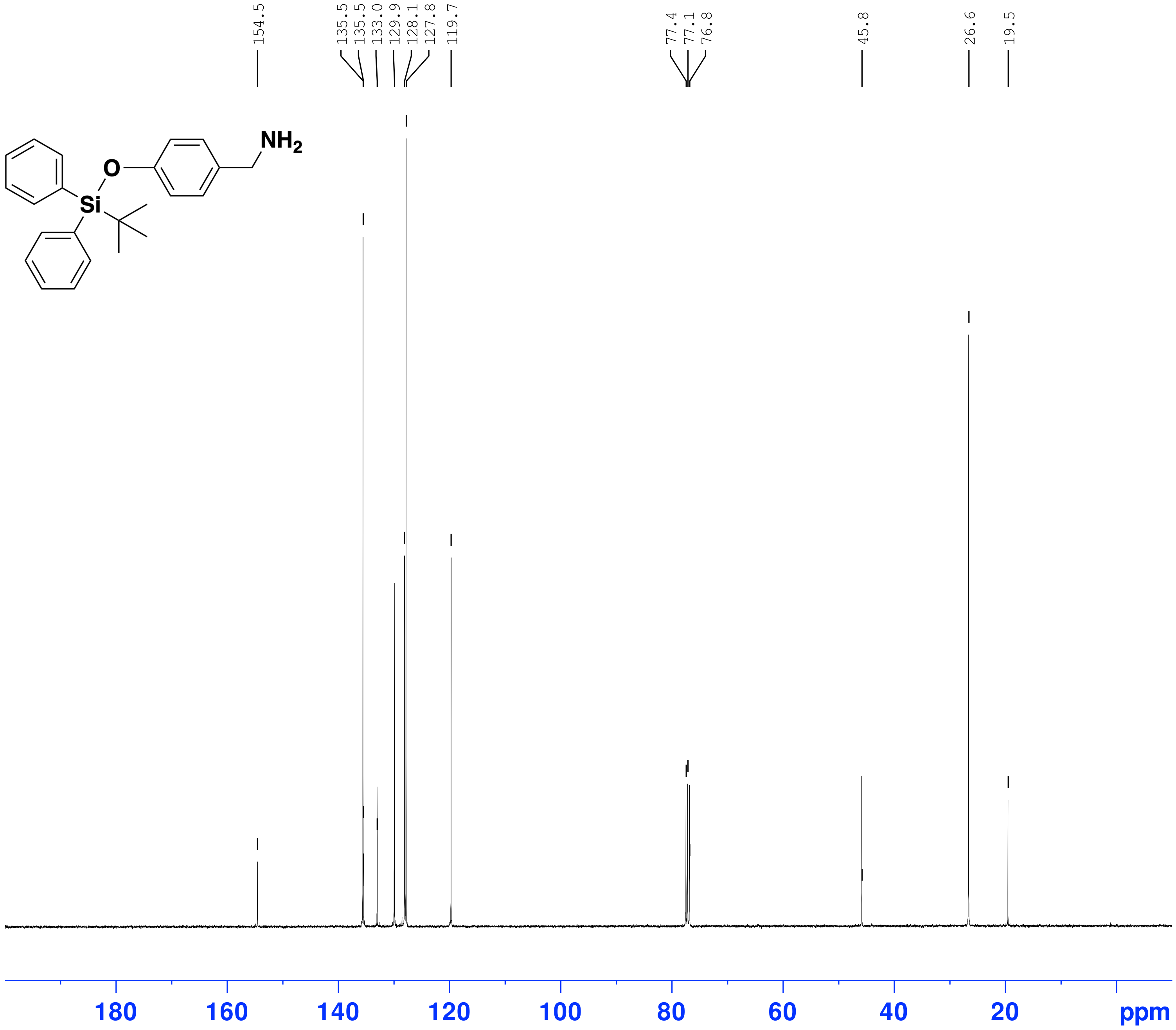

Current Data Parameters
NAME Oct04-2021-PTlab
EXPNO 11
PROCNO 1

F2 - Acquisition Parameters
Date_ 20211004
Time 14.58
INSTRUM spect
PROBHD 5 mm PABBO BB/
PULPROG zgpg30
TD 65536
SOLVENT CDCl3
NS 512
DS 4
SWH 24038.461 Hz
FIDRES 0.366798 Hz
AQ 1.3631488 sec
RG 2050
DW 20.800 usec
DE 6.50 usec
TE 300.0 K
D1 2.00000000 sec
D11 0.03000000 sec
TD0 1

======== CHANNEL f1 ========
SFO1 100.6605506 MHz
NUC1 13C
P1 11.00 usec
PLW1 48.00000000 W

======== CHANNEL f2 ========
SFO2 400.2816011 MHz
NUC2 1H
CPDPRG[2 waltz16
PCPD2 90.00 usec
PLW2 17.00000000 W
PLW12 0.24753000 W
PLW13 0.20050000 W

F2 - Processing parameters
SI 32768
SF 100.6504861 MHz
WDW EM
SSB 0
LB 1.00 Hz
GB 0
PC 1.40

^{1}H NMR (400 MHz, $CDCl_3$), 3-bromo-*N*-(4-((*tert*-butyldiphenylsilyl)oxy)benzyl)-5-iodobenzamide **(10)**

AVC-76
CDCl3

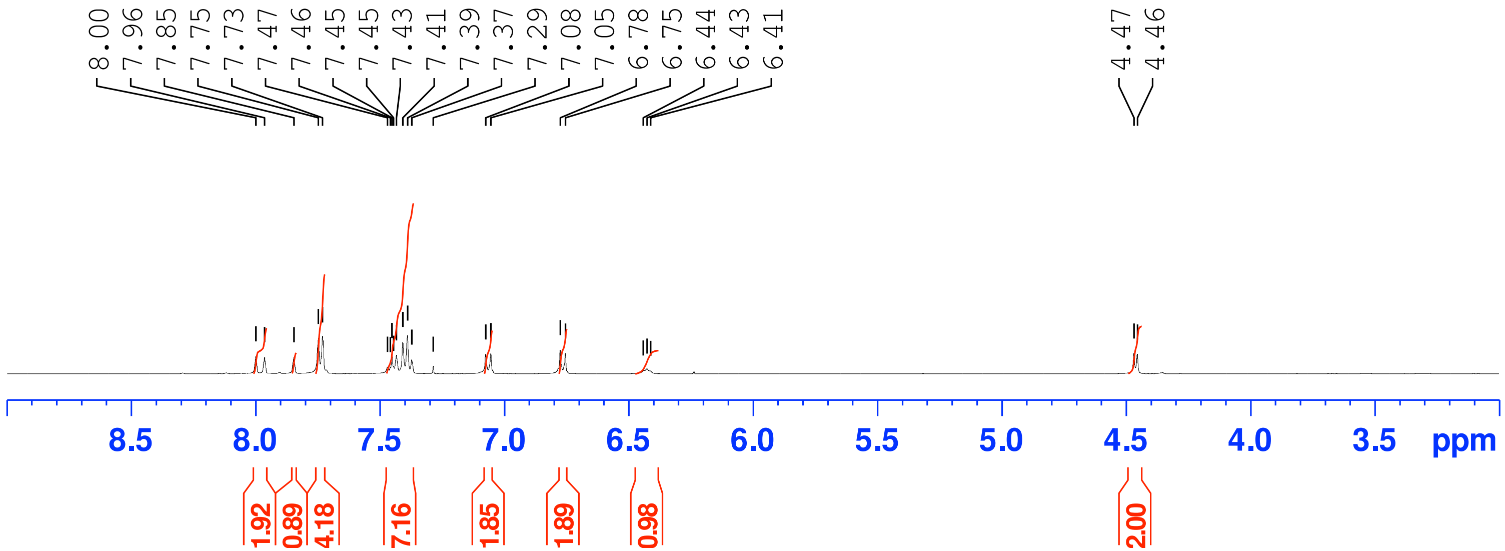

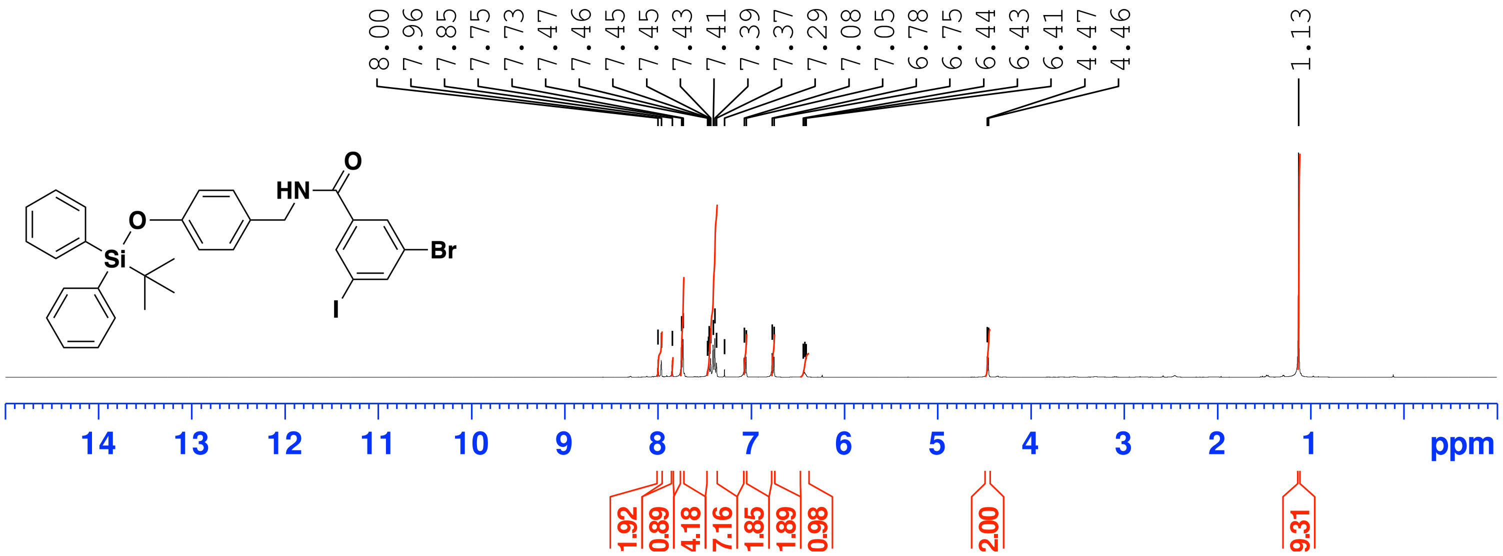

Current Data Parameters
NAME Oct11-2021-PTlab
EXPNO 10
PROCNO 1

F2 - Acquisition Parameters
Date_ 20211011
Time 9.52
INSTRUM spect
PROBHD 5 mm PABBO BB/
PULPROG zg30
TD 30046
SOLVENT CDCl3
NS 16
DS 1
SWH 8012.820 Hz
FIDRES 0.266685 Hz
AQ 1.8748704 sec
RG 101
DW 62.400 usec
DE 6.50 usec
TE 300.1 K
D1 6.00000000 sec
TD0 1

======== CHANNEL f1 ========
SFO1 400.2821952 MHz
NUC1 1H
P1 10.86 usec
PLW1 17.00000000 W

F2 - Processing parameters
SI 131072
SF 400.2800000 MHz
WDW EM
SSB 0
LB 0.20 Hz
GB 0
PC 1.00

^{13}C NMR (100 MHz, $CDCl_3$), 3-bromo-N-(4-((*tert*-butyldiphenylsilyl)oxy)benzyl)-5-iodobenzamide **(10)**

AVC-76
CDCl3

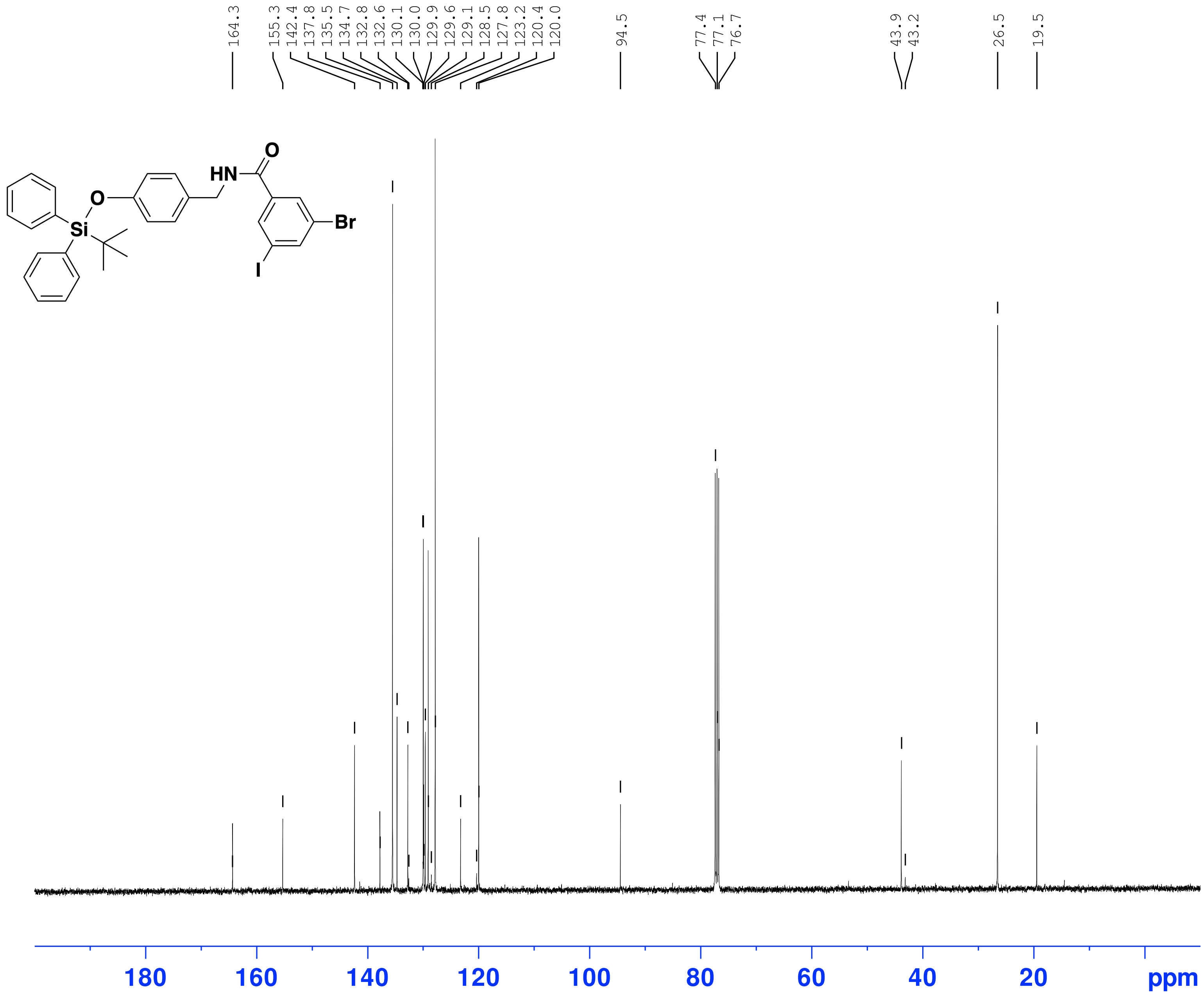

Current Data Parameters
NAME Oct11-2021-PTlab
EXPNO 11
PROCNO 1

F2 - Acquisition Parameters
Date_ 20211011
Time 10.30
INSTRUM spect
PROBHD 5 mm PABBO BB/
PULPROG zgpg30
TD 65536
SOLVENT CDCl3
NS 512
DS 4
SWH 24038.461 Hz
FIDRES 0.366798 Hz
AQ 1.3631488 sec
RG 2050
DW 20.800 usec
DE 6.50 usec
TE 300.1 K
D1 2.00000000 sec
D11 0.03000000 sec
TD0 1

======== CHANNEL f1 ========
SFO1 100.6605506 MHz
NUC1 13C
P1 11.00 usec
PLW1 48.00000000 W

======== CHANNEL f2 ========
SFO2 400.2816011 MHz
NUC2 1H
CPDPRG[2 waltz16
PCPD2 90.00 usec
PLW2 17.00000000 W
PLW12 0.24753000 W
PLW13 0.20050000 W

F2 - Processing parameters
SI 32768
SF 100.6504861 MHz
WDW EM
SSB 0
LB 1.00 Hz
GB 0
PC 1.40

^{1}H NMR (400 MHz, $CDCl_3$), Methyl (*E*)-3-(3-bromo-5-((4-((*tert*- butyldiphenylsilyl)oxy)benzyl)carbamoyl)phenyl)acrylate **(11)**

AVC-98 (F1)
CDCl3

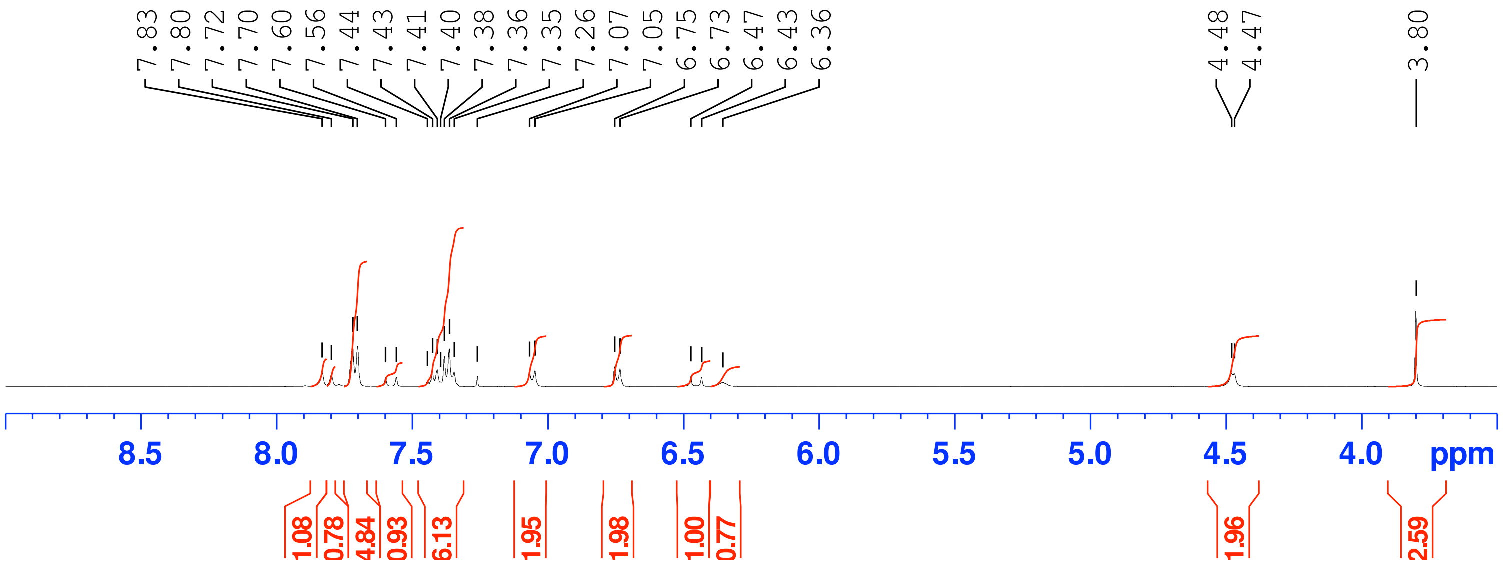

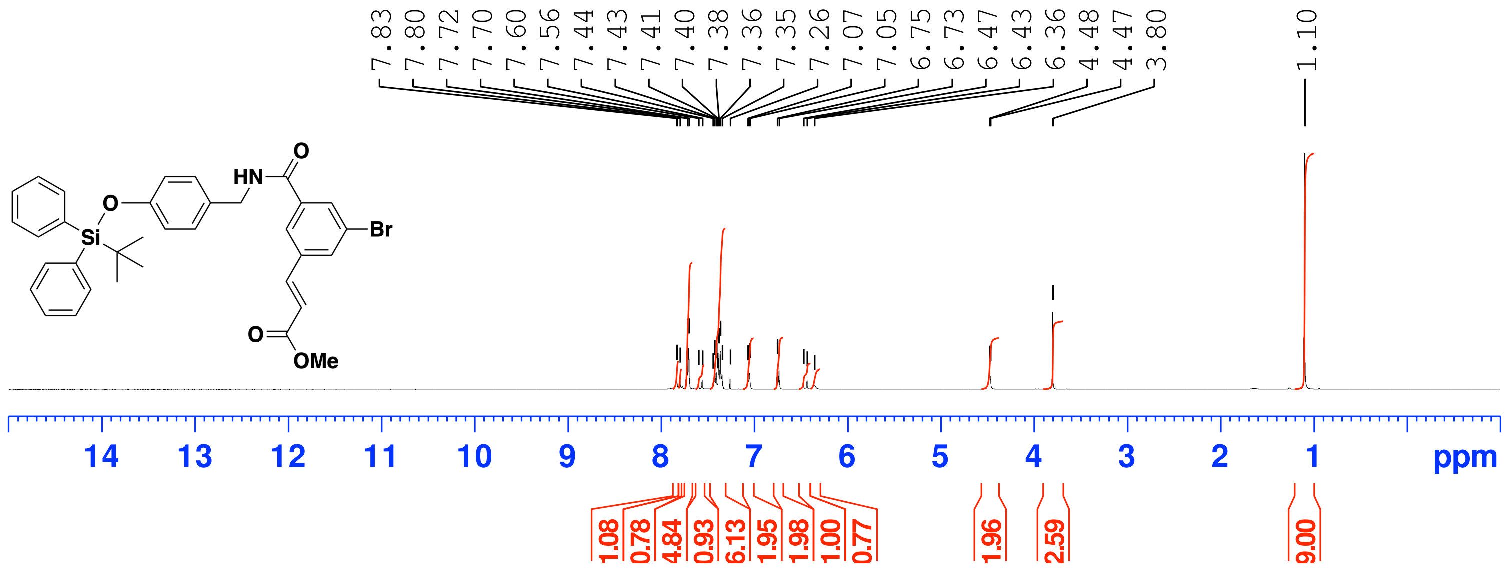

Current Data Parameters
NAME Dec13-2021-PTlab
EXPNO 10
PROCNO 1

F2 - Acquisition Parameters
Date_ 20211213
Time 10.29
INSTRUM spect
PROBHD 5 mm PABBO BB/
PULPROG zg30
TD 30046
SOLVENT CDCl3
NS 16
DS 1
SWH 8012.820 Hz
FIDRES 0.266685 Hz
AQ 1.8748704 sec
RG 114
DW 62.400 usec
DE 6.50 usec
TE 300.0 K
D1 6.00000000 sec
TD0 1

======== CHANNEL f1 ========
SFO1 400.2821952 MHz
NUC1 1H
P1 10.86 usec
PLW1 17.00000000 W

F2 - Processing parameters
SI 131072
SF 400.2800103 MHz
WDW EM
SSB 0
LB 0.20 Hz
GB 0
PC 1.00

^{13}C NMR (100 MHz, $CDCl_3$), Methyl (*E*)-3-(3-bromo-5-((4-((*tert*-butyldiphenylsilyl)oxy)benzyl)carbamoyl)phenyl)acrylate **(11)**

AVC-98 (F1)
CDCl3

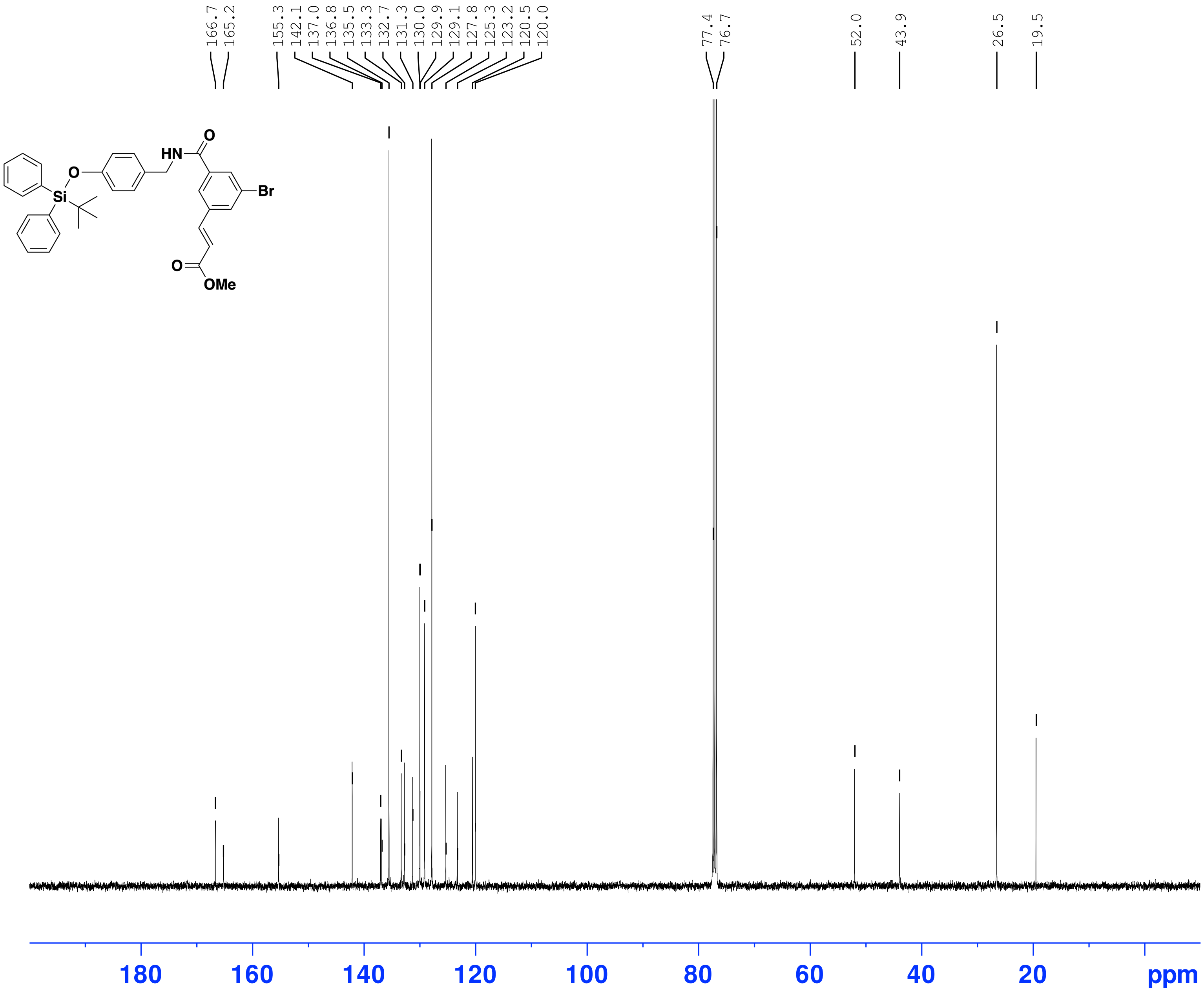

Current Data Parameters
NAME Dec13-2021-PTlab
EXPNO 30
PROCNO 1

F2 - Acquisition Parameters
Date_ 20211213
Time 13.50
INSTRUM spect
PROBHD 5 mm PABBO BB/
PULPROG zgpg30
TD 65536
SOLVENT CDCl3
NS 512
DS 4
SWH 24038.461 Hz
FIDRES 0.366798 Hz
AQ 1.3631488 sec
RG 2050
DW 20.800 usec
DE 6.50 usec
TE 300.0 K
D1 2.00000000 sec
D11 0.03000000 sec
TD0 1

======== CHANNEL f1 ========
SFO1 100.6605506 MHz
NUC1 13C
P1 11.00 usec
PLW1 48.00000000 W

======== CHANNEL f2 ========
SFO2 400.2816011 MHz
NUC2 1H
CPDPRG[2 waltz16
PCPD2 90.00 usec
PLW2 17.00000000 W
PLW12 0.24753000 W
PLW13 0.20050000 W

F2 - Processing parameters
SI 32768
SF 100.6504861 MHz
WDW EM
SSB 0
LB 1.00 Hz
GB 0
PC 1.40

^{1}H NMR (400 MHz, $CDCl_3$), Methyl (*E*)-3-(5-((4-((*tert*-butyldiphenylsilyl)oxy)benzyl)carbamoyl)-[1,1'-biphenyl]-3-yl)acrylate **(12)**

AVC-122(F2)
CDCl3

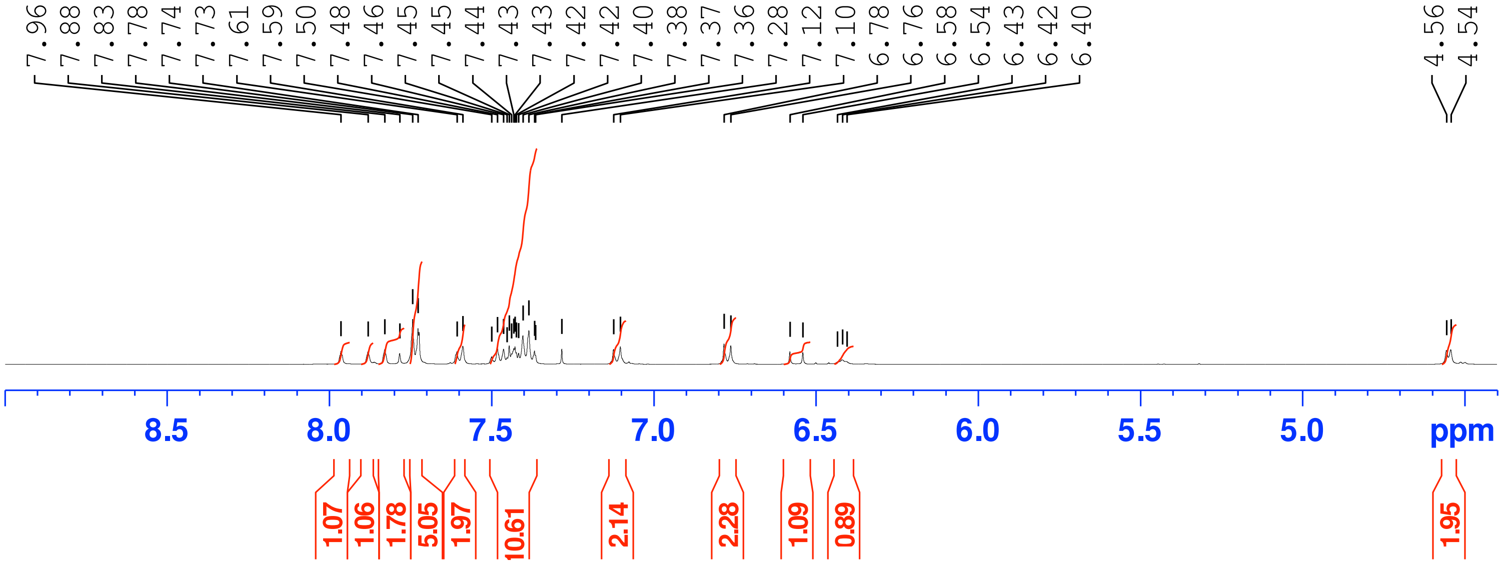

BRUKER

Current Data Parameters
NAME Mar11-2022-PTlab
EXPNO 40
PROCNO 1

F2 - Acquisition Parameters
Date_ 20220311
Time 15.35
INSTRUM spect
PROBHD 5 mm PABBO BB/
PULPROG zg30
TD 30046
SOLVENT CDCl3
NS 16
DS 1
SWH 8012.820 Hz
FIDRES 0.266685 Hz
AQ 1.8748704 sec
RG 256
DW 62.400 usec
DE 6.50 usec
TE 300.1 K
D1 6.00000000 sec
TD0 1

======== CHANNEL f1 ========
SFO1 400.2821952 MHz
NUC1 1H
P1 10.86 usec
PLW1 17.00000000 W

F2 - Processing parameters
SI 131072
SF 400.2800000 MHz
WDW EM
SSB 0
LB 0.20 Hz
GB 0
PC 1.00

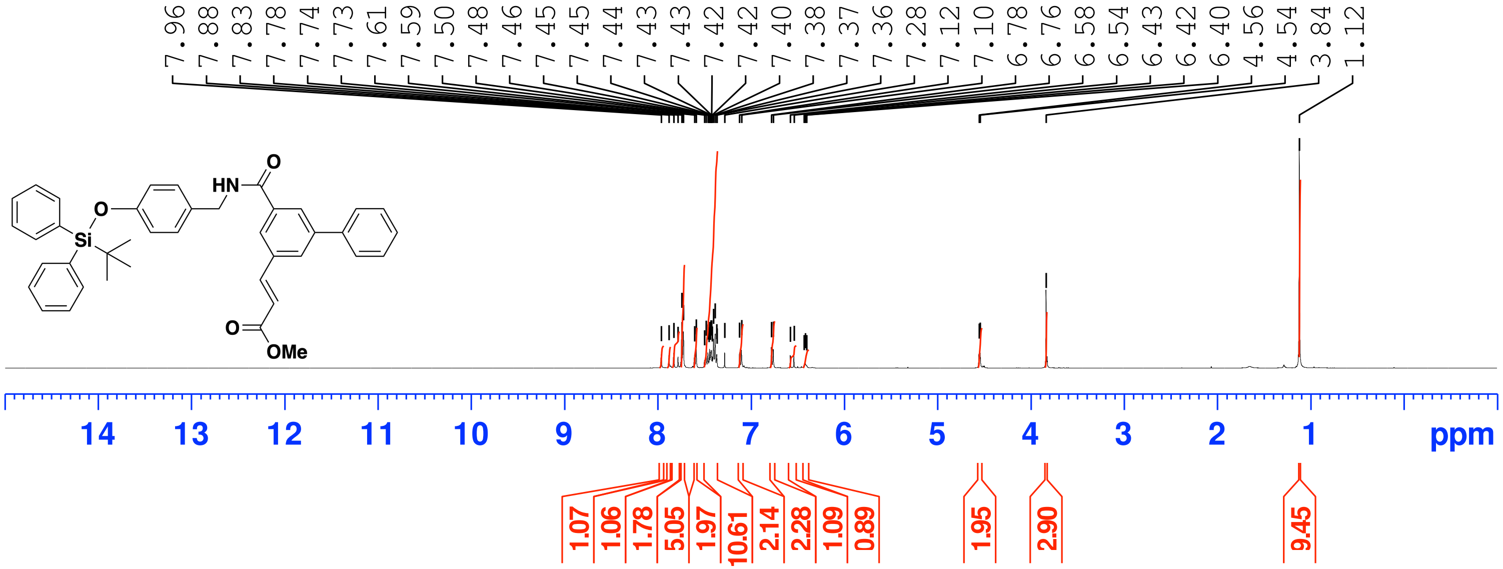

^{13}C NMR (100 MHz, $CDCl_3$), Methyl (*E*)-3-(5-((4-((*tert*-butyldiphenylsilyl)oxy)benzyl)carbamoyl)-[1,1'-biphenyl]-3-yl)acrylate **(12)**

AVC-122(F2)
CDCl3

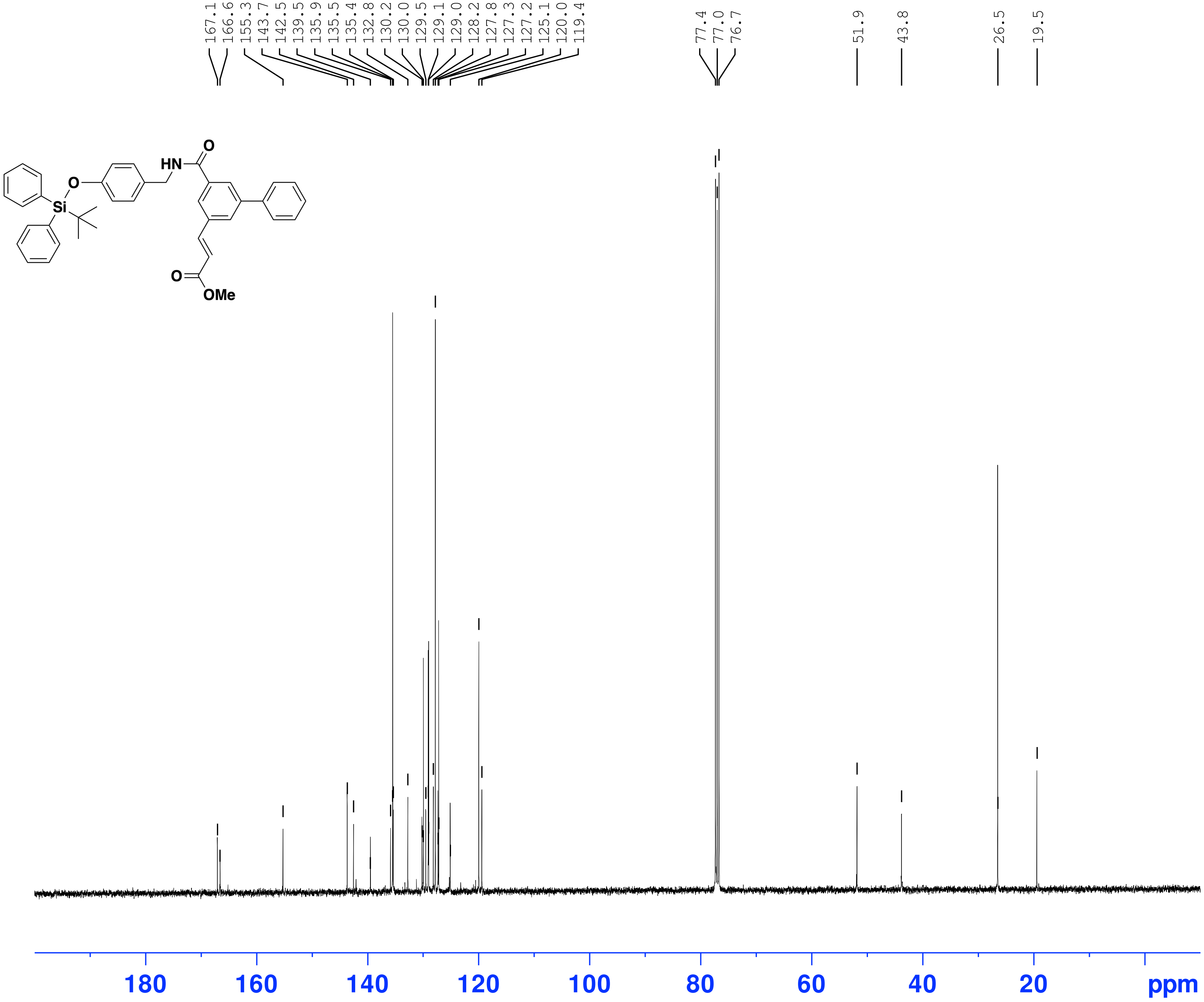

BRUKER

Current Data Parameters
NAME Mar11-2022-PTlab
EXPNO 41
PROCNO 1

F2 - Acquisition Parameters
Date_ 20220314
Time 19.06
INSTRUM spect
PROBHD 5 mm PABBO BB/
PULPROG zgpg30
TD 65536
SOLVENT CDCl3
NS 1024
DS 4
SWH 24038.461 Hz
FIDRES 0.366798 Hz
AQ 1.3631488 sec
RG 2050
DW 20.800 usec
DE 6.50 usec
TE 300.0 K
D1 2.00000000 sec
D11 0.03000000 sec
TD0 1

======== CHANNEL f1 ========
SFO1 100.6605506 MHz
NUC1 13C
P1 11.00 usec
PLW1 48.00000000 W

======== CHANNEL f2 ========
SFO2 400.2816011 MHz
NUC2 1H
CPDPRG[2 waltz16
PCPD2 90.00 usec
PLW2 17.00000000 W
PLW12 0.24753000 W
PLW13 0.20050000 W

F2 - Processing parameters
SI 32768
SF 100.6504861 MHz
WDW EM
SSB 0
LB 1.00 Hz
GB 0
PC 1.40

^{1}H NMR (400 MHz, $(CD_3)_2SO$), Methyl (*E*)-3-(5-((4-hydroxybenzyl)carbamoyl)-[1,1'-biphenyl]-3-yl)acrylate **(13)**

AVC-2-44
DMSO

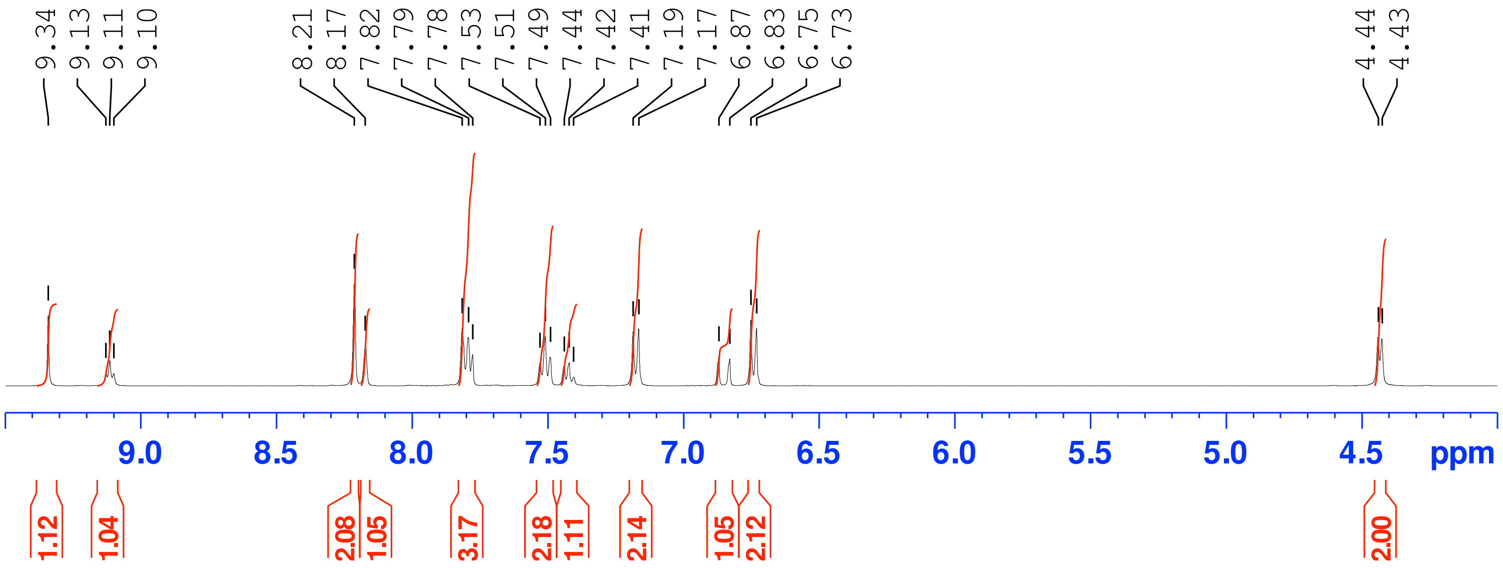

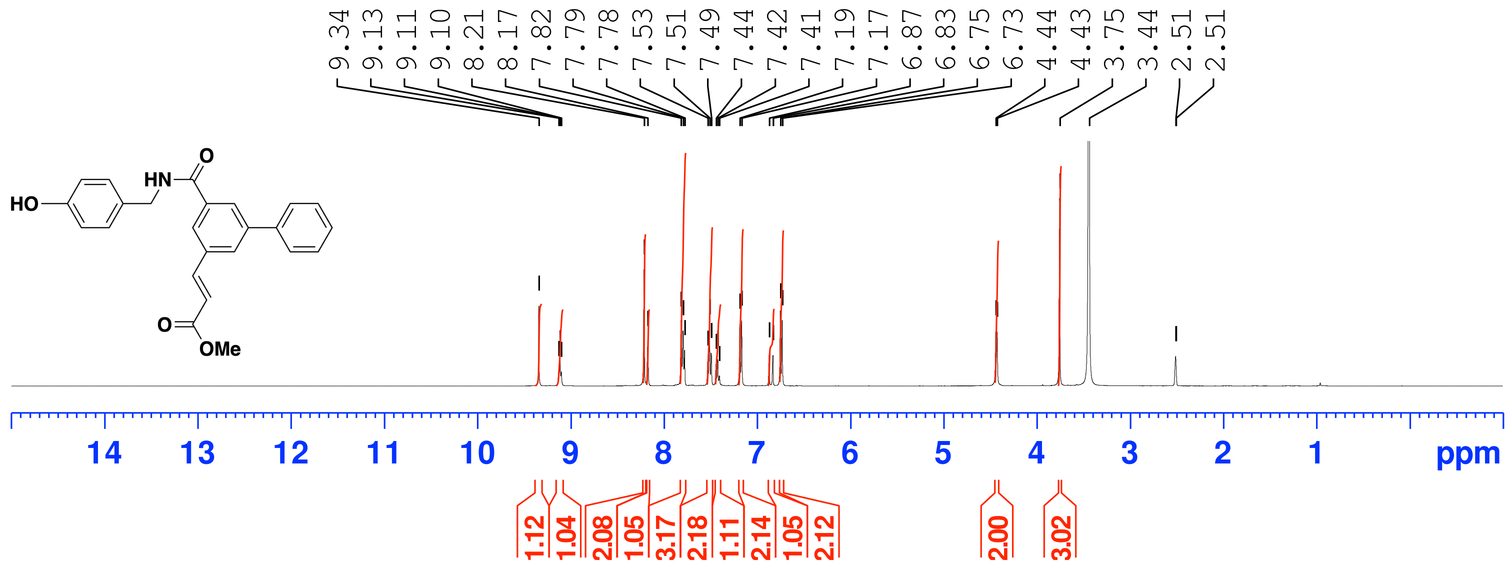

Current Data Parameters
NAME May31-2022-PTlab
EXPNO 60
PROCNO 1

F2 - Acquisition Parameters
Date_ 20220531
Time 16.31
INSTRUM spect
PROBHD 5 mm PABBO BB/
PULPROG zg30
TD 30046
SOLVENT DMSO
NS 16
DS 1
SWH 8012.820 Hz
FIDRES 0.266685 Hz
AQ 1.8748704 sec
RG 80.6
DW 62.400 usec
DE 6.50 usec
TE 300.1 K
D1 6.00000000 sec
TD0 1

======== CHANNEL f1 ========
SFO1 400.2821952 MHz
NUC1 1H
P1 10.86 usec
PLW1 17.00000000 W

F2 - Processing parameters
SI 131072
SF 400.2800000 MHz
WDW EM
SSB 0
LB 0.20 Hz
GB 0
PC 1.00

^{13}C NMR (100 MHz, $(CD_3)_2SO$), Methyl (*E*)-3-(5-((4-hydroxybenzyl)carbamoyl)-[1,1'-biphenyl]-3-yl)acrylate **(13)**

AVC-2-44
DMSO

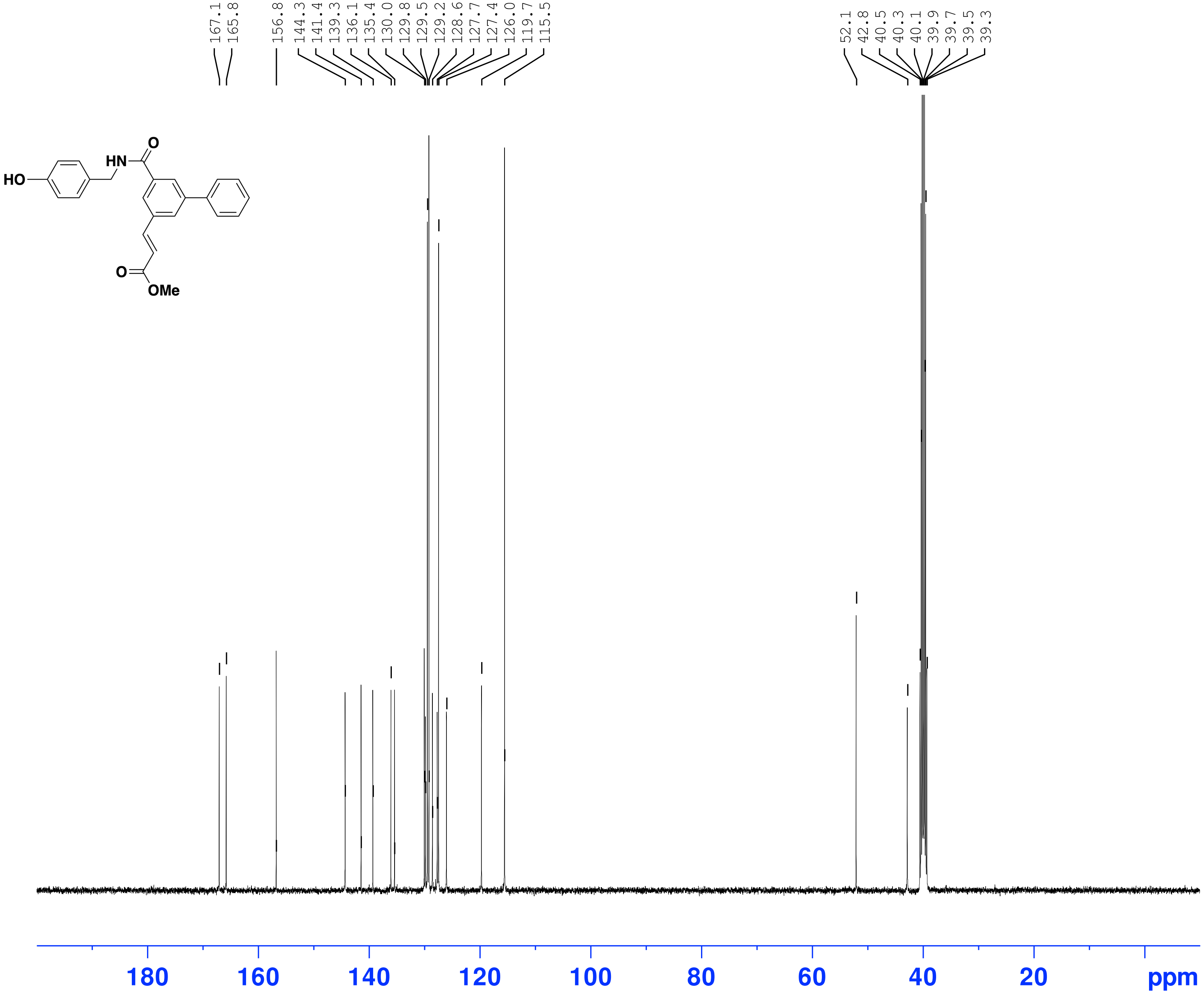

Current Data Parameters
NAME May31-2022-PTlab
EXPNO 61
PROCNO 1

F2 - Acquisition Parameters
Date_ 20220531
Time 18.06
INSTRUM spect
PROBHD 5 mm PABBO BB/
PULPROG zgpg30
TD 65536
SOLVENT DMSO
NS 1536
DS 4
SWH 24038.461 Hz
FIDRES 0.366798 Hz
AQ 1.3631488 sec
RG 2050
DW 20.800 usec
DE 6.50 usec
TE 300.1 K
D1 2.00000000 sec
D11 0.03000000 sec
TD0 1

======== CHANNEL f1 ========
SFO1 100.6605506 MHz
NUC1 13C
P1 11.00 usec
PLW1 48.00000000 W

======== CHANNEL f2 ========
SFO2 400.2816011 MHz
NUC2 1H
CPDPRG[2 waltz16
PCPD2 90.00 usec
PLW2 17.00000000 W
PLW12 0.24753000 W
PLW13 0.20050000 W

F2 - Processing parameters
SI 32768
SF 100.6504861 MHz
WDW EM
SSB 0
LB 1.00 Hz
GB 0
PC 1.40

^{1}H NMR (400 MHz, $CDCl_3$), Methyl(*E*)-3-(5-((4-(prop-2-yn-1-yloxy)benzyl)carbamoyl)-[1,1'-biphenyl]-3-yl)acrylate **(14)**

AVC-2-49
CDCl3

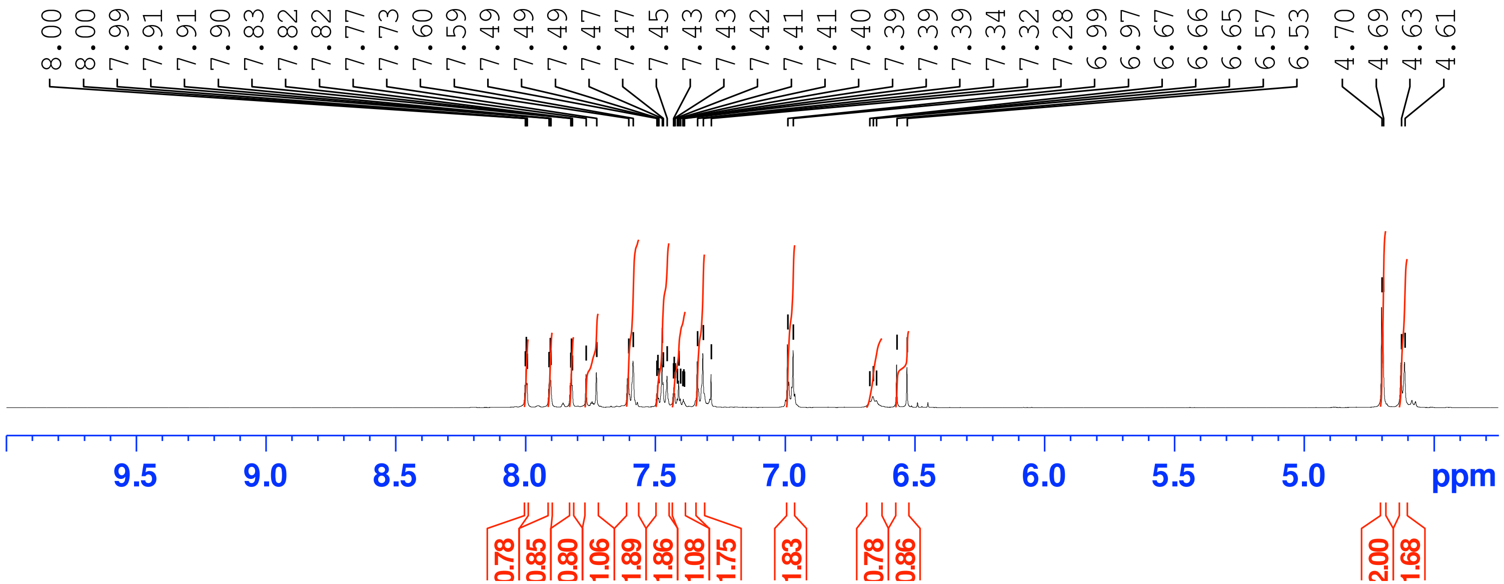

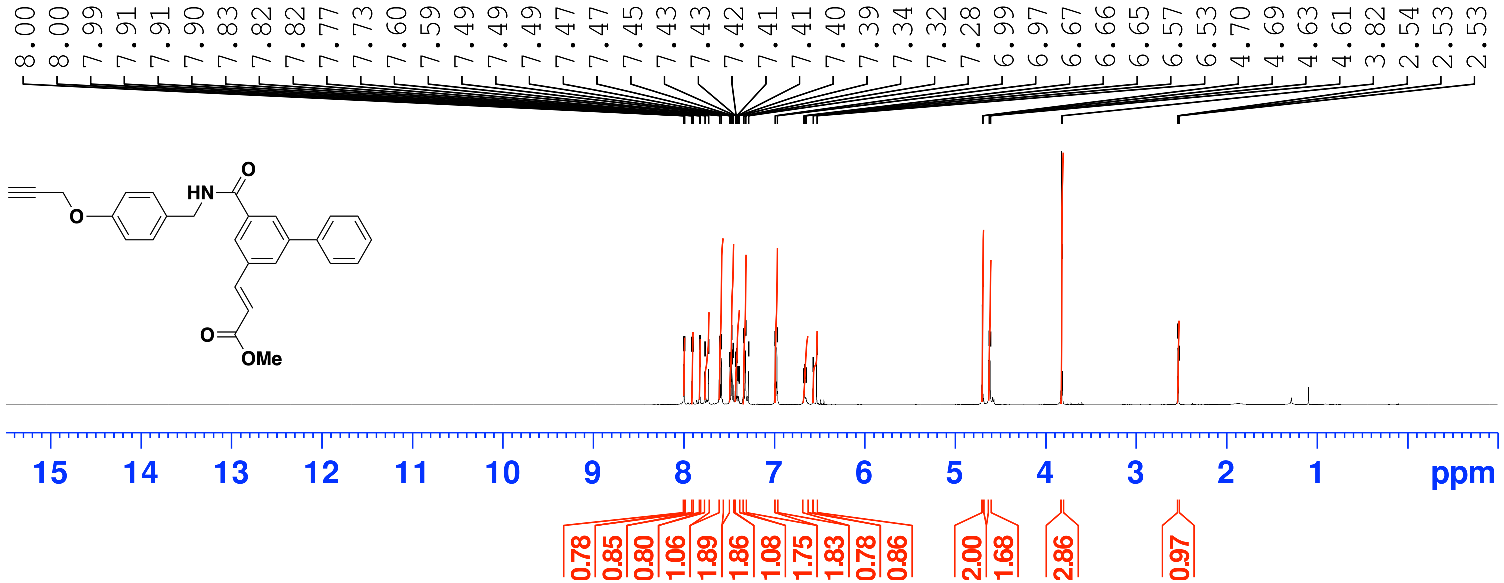

Current Data Parameters
NAME Jul27-2022-PTlab
EXPNO 20
PROCNO 1

F2 - Acquisition Parameters
Date_ 20220727
Time 12.24
INSTRUM spect
PROBHD 5 mm PABBO BB/
PULPROG zg30
TD 30046
SOLVENT CDCl3
NS 16
DS 1
SWH 8012.820 Hz
FIDRES 0.266685 Hz
AQ 1.8748704 sec
RG 256
DW 62.400 usec
DE 6.50 usec
TE 300.0 K
D1 6.00000000 sec
TD0 1

======== CHANNEL f1 ========
SFO1 400.2821952 MHz
NUC1 1H
P1 10.86 usec
PLW1 17.00000000 W

F2 - Processing parameters
SI 131072
SF 400.2800000 MHz
WDW EM
SSB 0
LB 0.20 Hz
GB 0
PC 1.00

^{13}C NMR (100 MHz, $CDCl_3$), Methyl(*E*)-3-(5-((4-(prop-2-yn-1-yloxy)benzyl)carbamoyl)-[1,1'-biphenyl]-3-yl)acrylate **(14)**

AVC-2-49
CDCl3

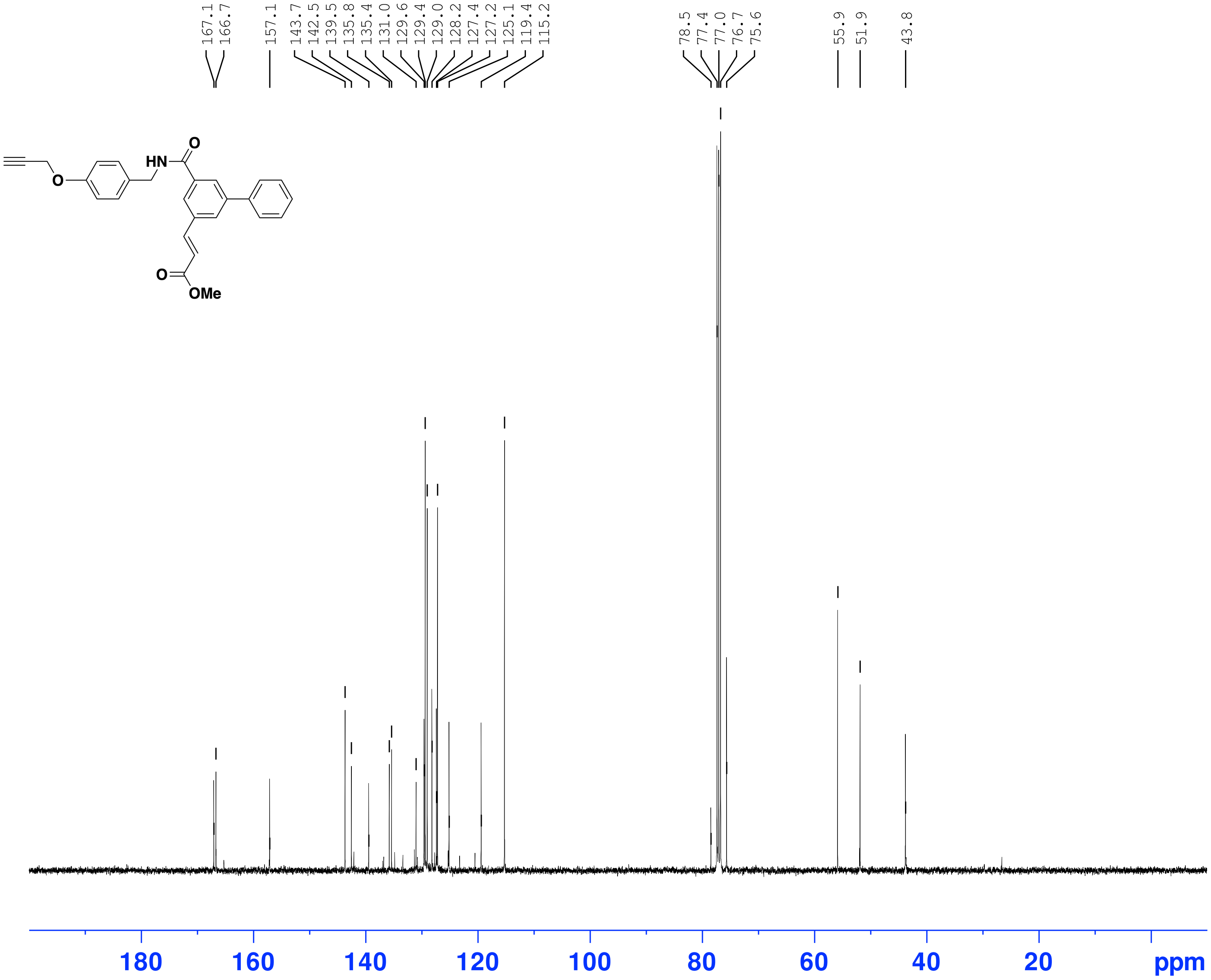

BRUKER

```
Current Data Parameters
NAME       Jul27-2022-PTlab
EXPNO                    21
PROCNO                    1

F2 - Acquisition Parameters
Date_              20220727
Time                  15.14
INSTRUM               spect
PROBHD   5 mm PABBO BB/
PULPROG             zgpg30
TD                    65536
SOLVENT               CDCl3
NS                     1024
DS                        4
SWH           24038.461 Hz
FIDRES         0.366798 Hz
AQ            1.3631488 sec
RG                     2050
DW               20.800 usec
DE                 6.50 usec
TE                300.0 K
D1           2.00000000 sec
D11          0.03000000 sec
TD0                       1

======== CHANNEL f1 ========
SFO1        100.6605506 MHz
NUC1                    13C
P1                11.00 usec
PLW1        48.00000000 W

======== CHANNEL f2 ========
SFO2        400.2816011 MHz
NUC2                     1H
CPDPRG[2            waltz16
PCPD2             90.00 usec
PLW2        17.00000000 W
PLW12        0.24753000 W
PLW13        0.20050000 W

F2 - Processing parameters
SI                    32768
SF          100.6504861 MHz
WDW                      EM
SSB                       0
LB                   1.00 Hz
GB                        0
PC                     1.40
```

^{1}H NMR (400 MHz, $(CD_3)_2SO$), (*E*)-3-(5-((4-Methylbenzyl)carbamoyl)-[1,1'-biphenyl]-3-yl)acrylic acid **(3)**

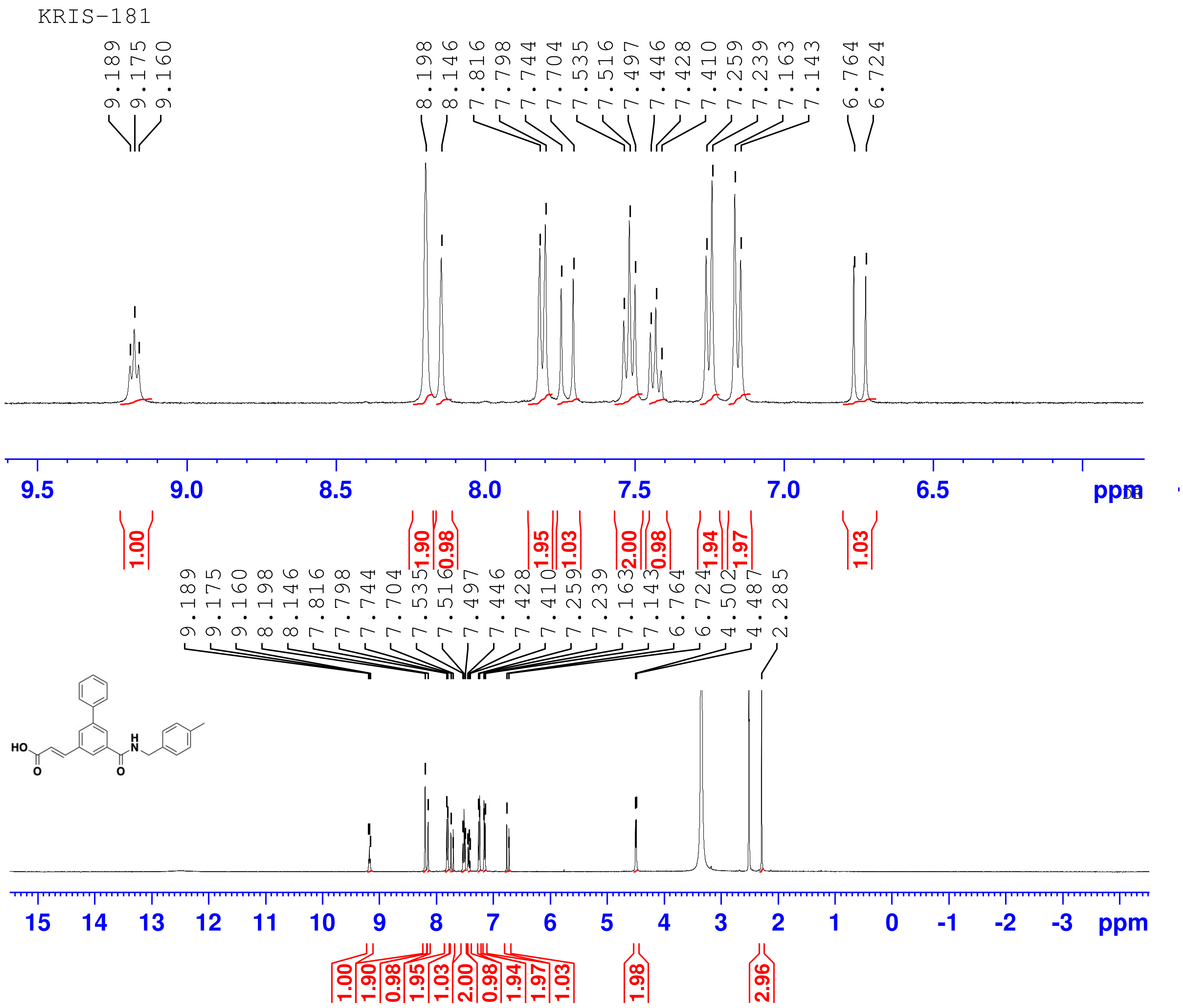


```
Current Data Parameters
NAME       Nov04-2020-PTlab
EXPNO                    20
PROCNO                    1

F2 - Acquisition Parameters
Date_              20201104
Time                  15.16
INSTRUM               spect
PROBHD    5 mm PABBO BB/
PULPROG                zg30
TD                    30046
SOLVENT                DMSO
NS                       16
DS                        1
SWH            8012.820 Hz
FIDRES         0.266685 Hz
AQ            1.8748704 sec
RG                      256
DW               62.400 usec
DE                6.50 usec
TE                300.1 K
D1           6.00000000 sec
TD0                       1

======== CHANNEL f1 ========
SFO1        400.2821952 MHz
NUC1                     1H
P1               10.86 usec
PLW1        17.00000000 W

F2 - Processing parameters
SI                   131072
SF          400.2800000 MHz
WDW                      EM
SSB       0
LB                  0.20 Hz
GB        0
PC                     1.00
```

^{13}C NMR (100 MHz, $(CD_3)_2SO$), (*E*)-3-(5-((4-Methylbenzyl)carbamoyl)-[1,1'-biphenyl]-3-yl)acrylic acid **(3)**

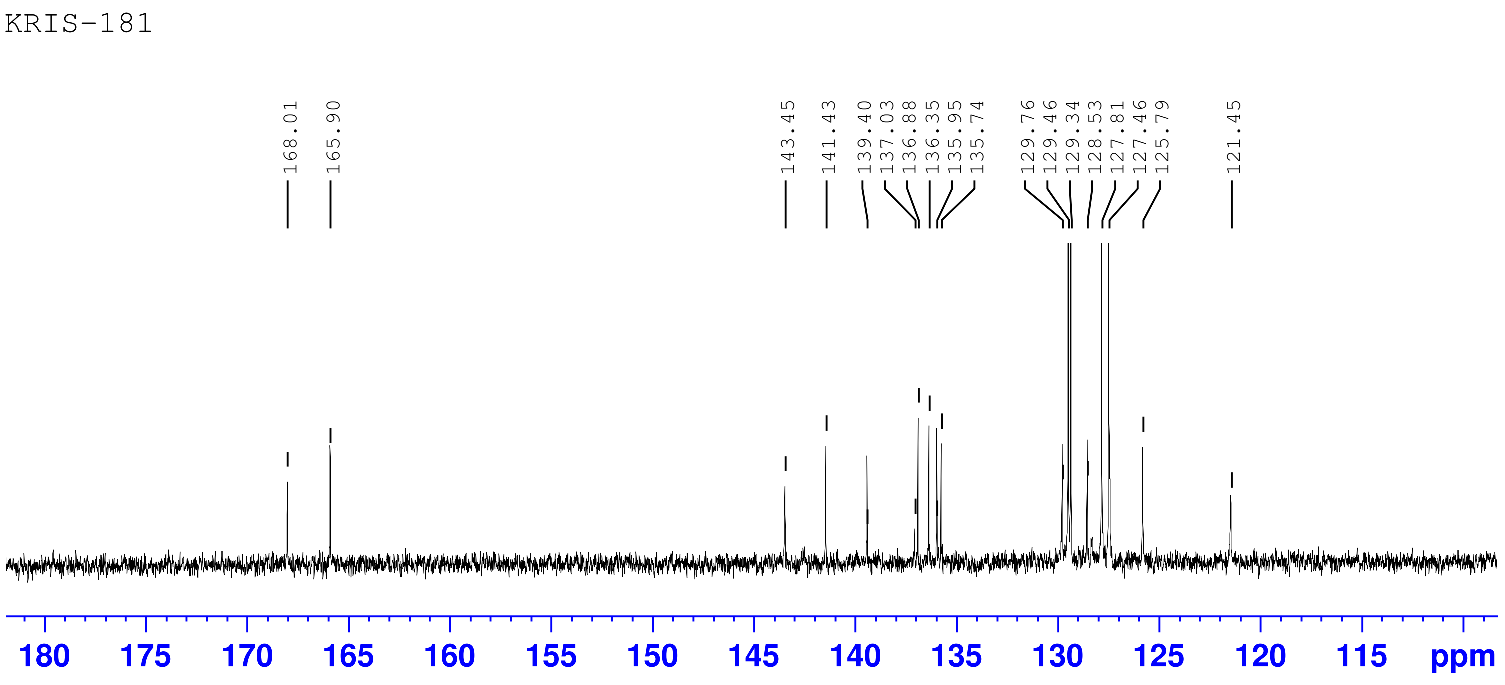

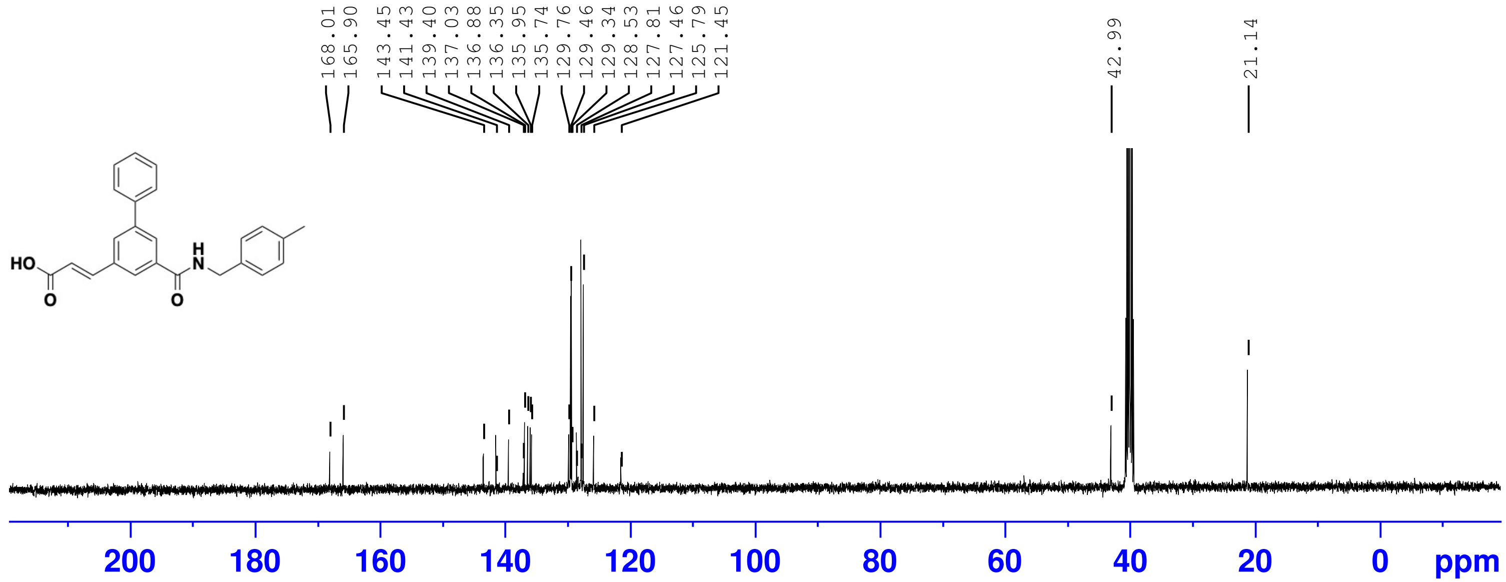

Current Data Parameters
NAME Nov03-2020-PTlab
EXPNO 50
PROCNO 1

F2 - Acquisition Parameters
Date_ 20201103
Time 14.55
INSTRUM spect
PROBHD 5 mm PABBO BB/
PULPROG zgpg30
TD 65536
SOLVENT DMSO
NS 350
DS 4
SWH 24038.461 Hz
FIDRES 0.366798 Hz
AQ 1.3631488 sec
RG 2050
DW 20.800 usec
DE 6.50 usec
TE 300.1 K
D1 2.00000000 sec
D11 0.03000000 sec
TD0 1

======== CHANNEL f1 ========
SFO1 100.6605506 MHz
NUC1 13C
P1 11.00 usec
PLW1 48.00000000 W

======== CHANNEL f2 ========
SFO2 400.2816011 MHz
NUC2 1H
CPDPRG[2 waltz16
PCPD2 90.00 usec
PLW2 17.00000000 W
PLW12 0.24753000 W
PLW13 0.20050000 W

F2 - Processing parameters
SI 32768
SF 100.6504861 MHz
WDW EM
SSB 0
LB 1.00 Hz
GB 0
PC 1.40

^{1}H NMR (400 MHz, $CDCl_3$), *tert*-butyl 2-(2-(2-azidoethoxy)ethoxy)acetate **(16)**

AVC-118-3
CDCl3

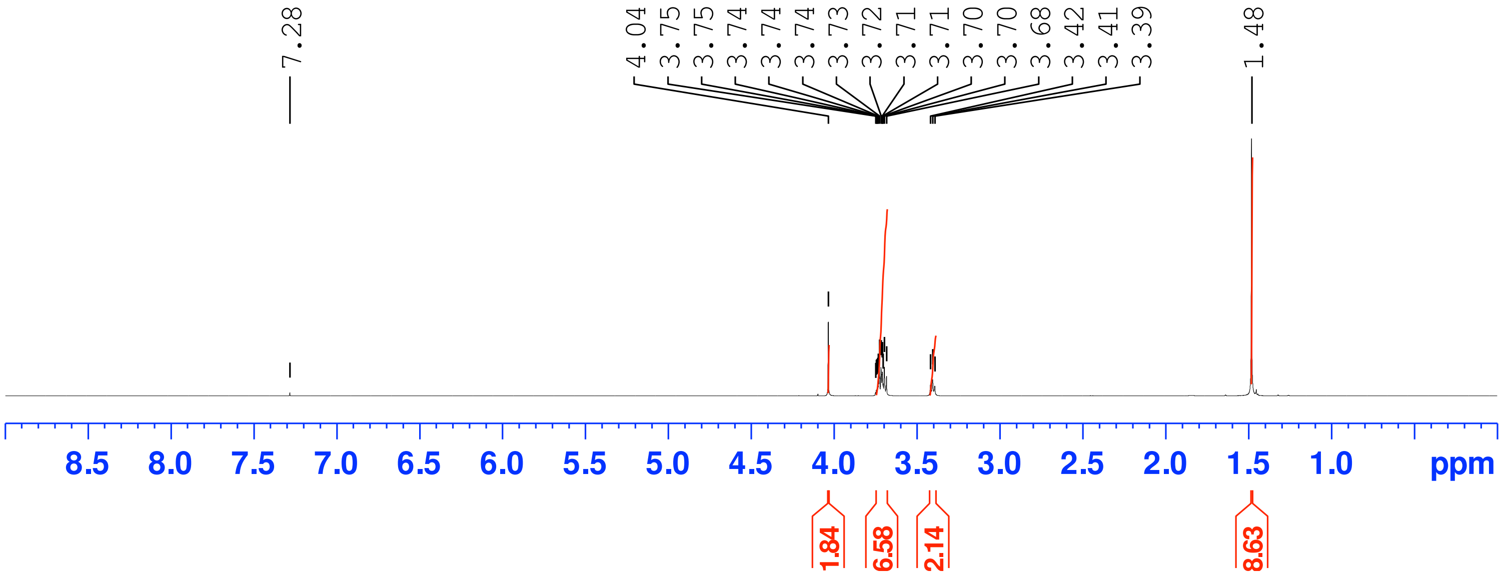

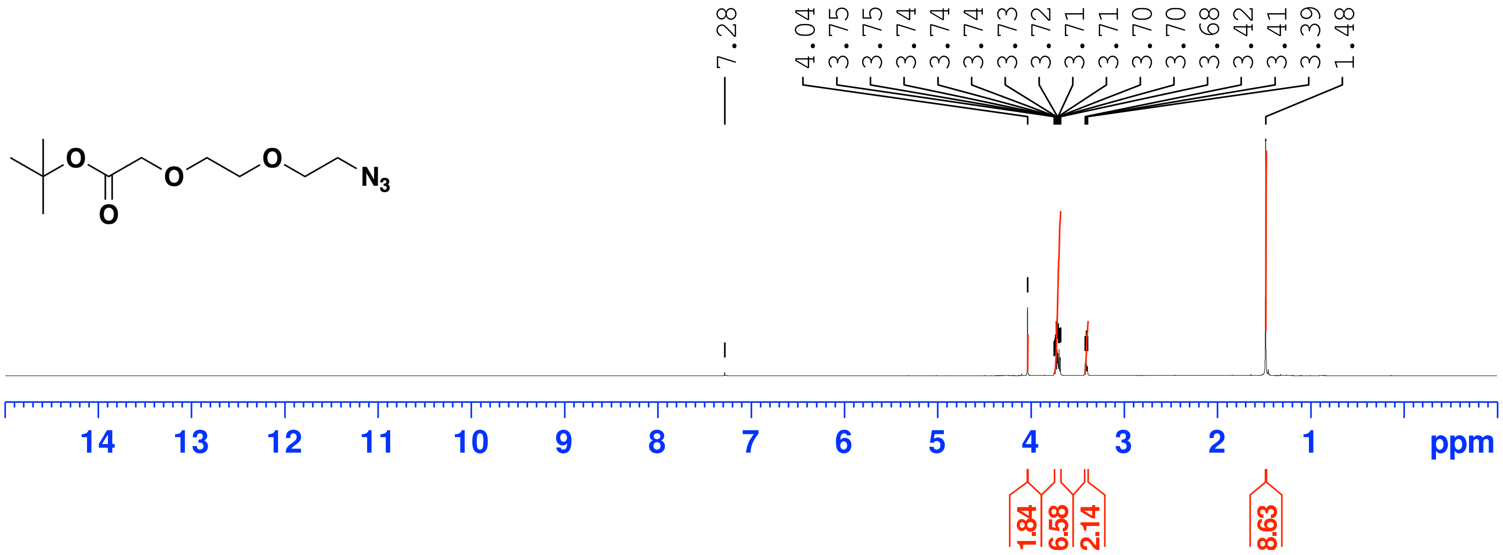

Current Data Parameters
NAME Feb25-2022-PTlab
EXPNO 50
PROCNO 1

F2 - Acquisition Parameters
Date_ 20220225
Time 15.58
INSTRUM spect
PROBHD 5 mm PABBO BB/
PULPROG zg30
TD 30046
SOLVENT CDCl3
NS 16
DS 1
SWH 8012.820 Hz
FIDRES 0.266685 Hz
AQ 1.8748704 sec
RG 80.6
DW 62.400 usec
DE 6.50 usec
TE 300.1 K
D1 6.00000000 sec
TD0 1

======== CHANNEL f1 ========
SFO1 400.2821952 MHz
NUC1 1H
P1 10.86 usec
PLW1 17.00000000 W

F2 - Processing parameters
SI 131072
SF 400.2800000 MHz
WDW EM
SSB 0
LB 0.20 Hz
GB 0
PC 1.00

^{13}C NMR (100 MHz, $CDCl_3$), *tert*-butyl 2-(2-(2-azidoethoxy)ethoxy)acetate **(16)**

AVC-118-3
CDCl3

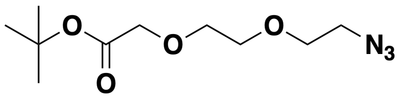

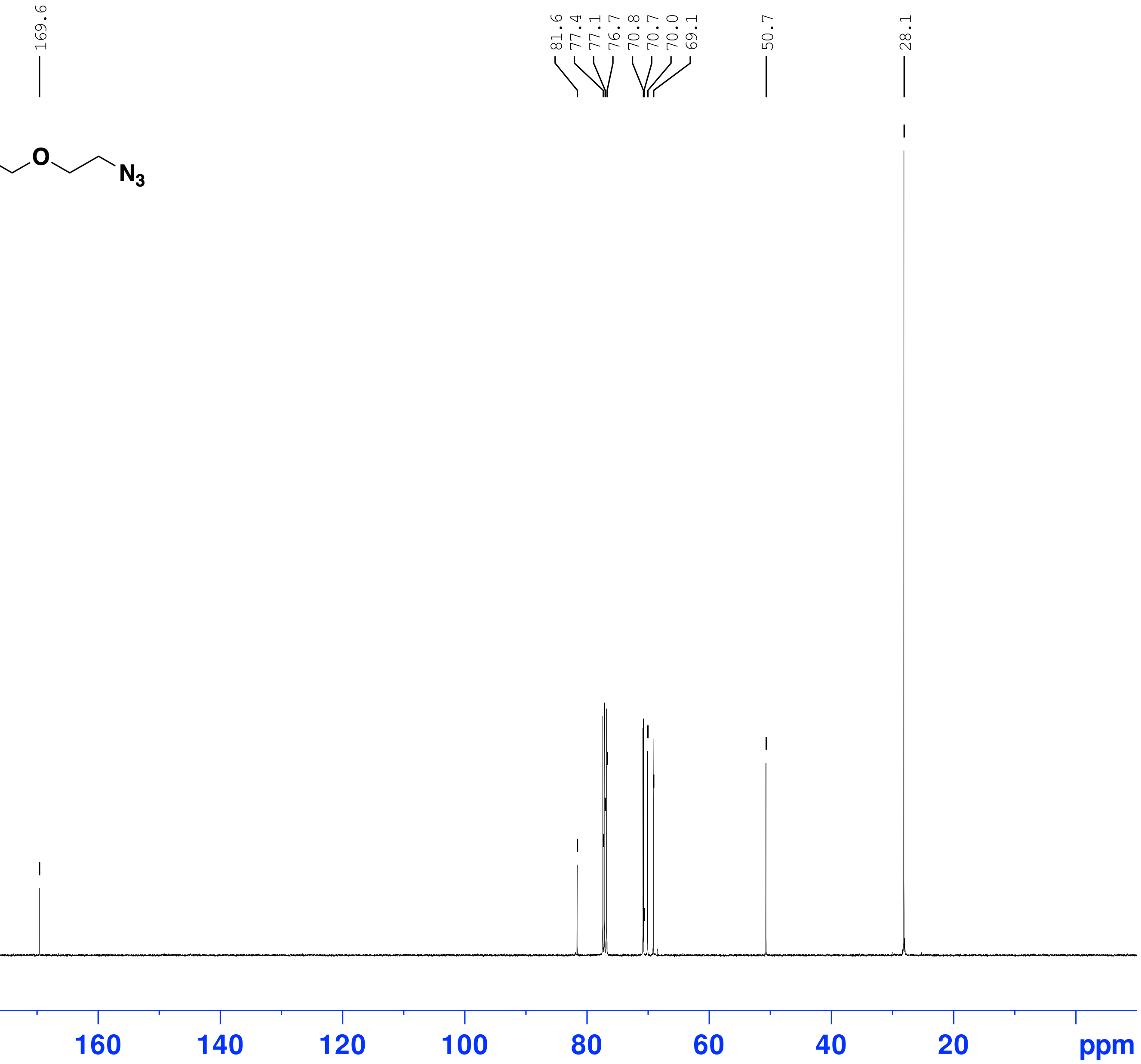

BRUKER

Current Data Parameters
NAME Feb25-2022-PTlab
EXPNO 51
PROCNO 1

F2 - Acquisition Parameters
Date_ 20220225
Time 19.08
INSTRUM spect
PROBHD 5 mm PABBO BB/
PULPROG zgpg30
TD 65536
SOLVENT CDCl3
NS 1024
DS 4
SWH 24038.461 Hz
FIDRES 0.366798 Hz
AQ 1.3631488 sec
RG 2050
DW 20.800 usec
DE 6.50 usec
TE 300.0 K
D1 2.00000000 sec
D11 0.03000000 sec
TD0 1

======== CHANNEL f1 ========
SFO1 100.6605506 MHz
NUC1 13C
P1 11.00 usec
PLW1 48.00000000 W

======== CHANNEL f2 ========
SFO2 400.2816011 MHz
NUC2 1H
CPDPRG[2 waltz16
PCPD2 90.00 usec
PLW2 17.00000000 W
PLW12 0.24753000 W
PLW13 0.20050000 W

F2 - Processing parameters
SI 32768
SF 100.6504861 MHz
WDW EM
SSB 0
LB 1.00 Hz
GB 0
PC 1.40

^{1}H NMR (400 MHz, $CDCl_3$), 2-(2-(2-azidoethoxy)ethoxy)-*N*-(2-(2,6-dioxopiperidin-3-yl)-1-oxoisoindolin-4-yl)acetamide **(18)**

AVC-2-1
CDCl3

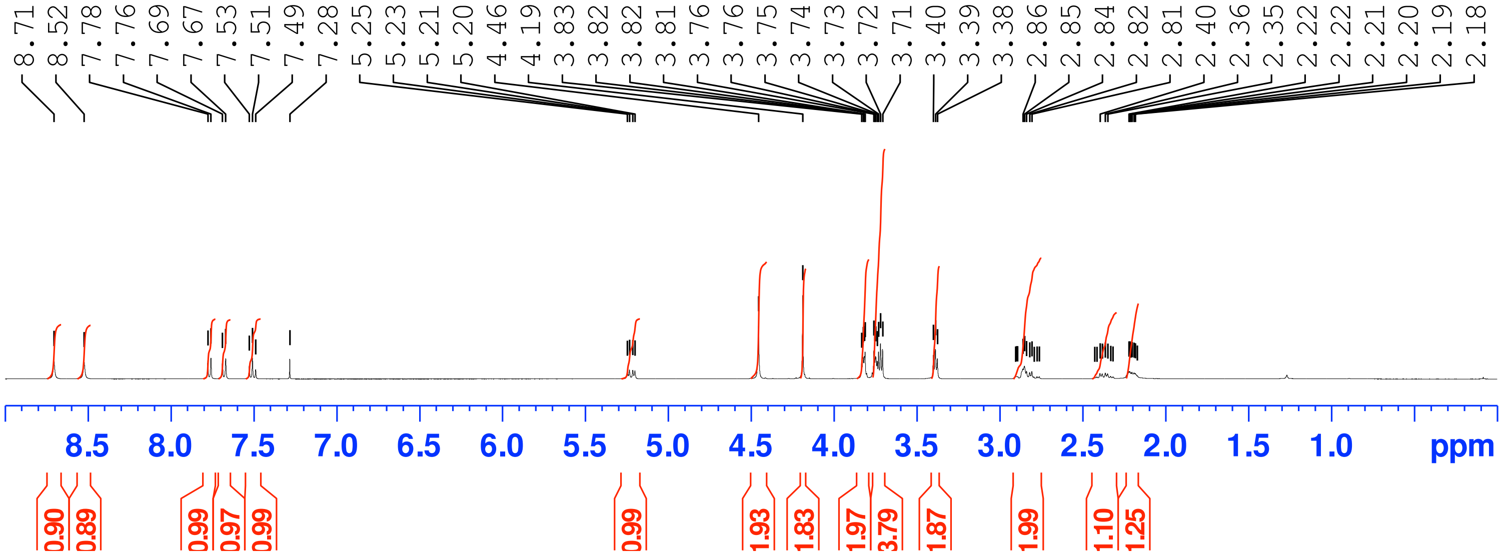

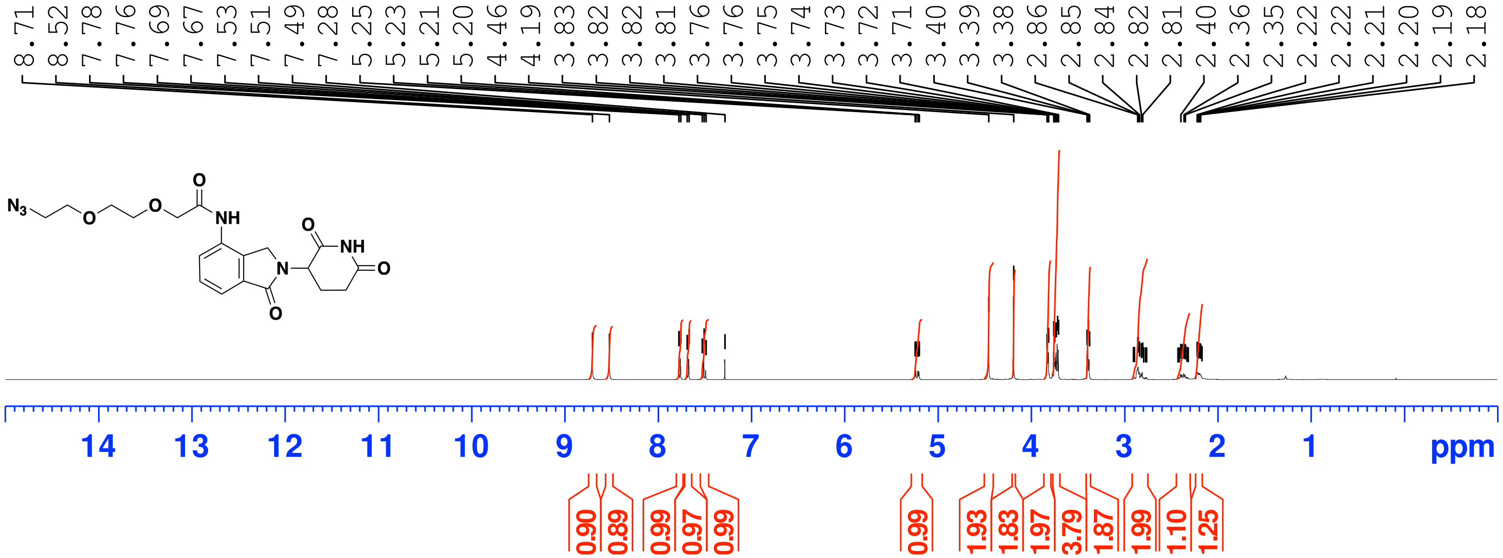

BRUKER

Current Data Parameters
NAME Apr12-2022-PTlab
EXPNO 10
PROCNO 1

F2 - Acquisition Parameters
Date_ 20220412
Time 15.41
INSTRUM spect
PROBHD 5 mm PABBO BB/
PULPROG zg30
TD 30046
SOLVENT CDCl3
NS 16
DS 1
SWH 8012.820 Hz
FIDRES 0.266685 Hz
AQ 1.8748704 sec
RG 256
DW 62.400 usec
DE 6.50 usec
TE 300.1 K
D1 6.00000000 sec
TD0 1

======== CHANNEL f1 ========
SFO1 400.2821952 MHz
NUC1 1H
P1 10.86 usec
PLW1 17.00000000 W

F2 - Processing parameters
SI 131072
SF 400.2800000 MHz
WDW EM
SSB 0
LB 0.20 Hz
GB 0
PC 1.00

^{13}C NMR (100 MHz, $CDCl_3$), 2-(2-(2-azidoethoxy)ethoxy)-*N*-(2-(2,6-dioxopiperidin-3-yl)-1-oxoisoindolin-4-yl)acetamide **(18)**

AVC-2-1
CDCl3

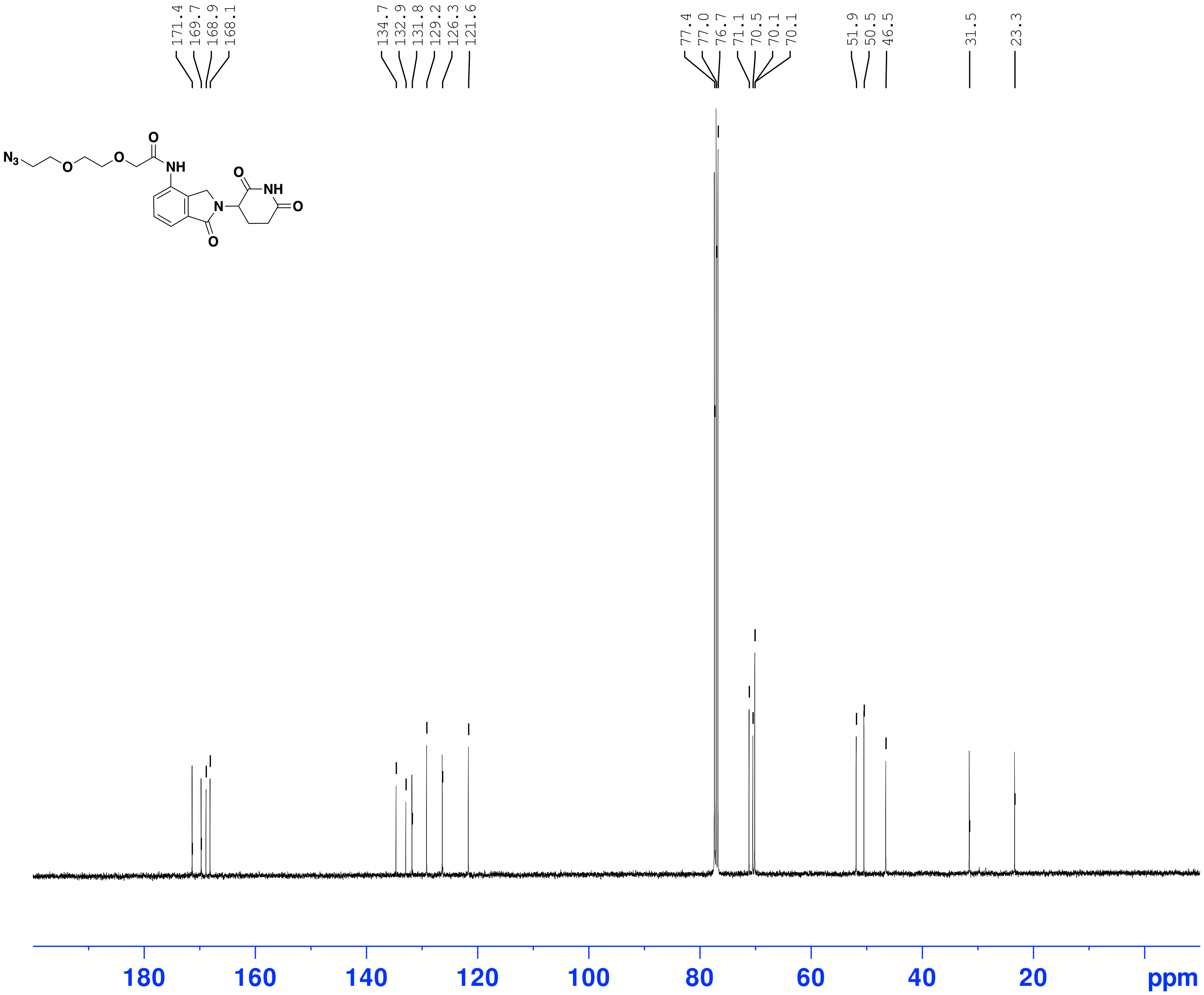

BRUKER

Current Data Parameters
NAME Apr12-2022-PTlab
EXPNO 11
PROCNO 1

F2 - Acquisition Parameters
Date_ 20220412
Time 19.06
INSTRUM spect
PROBHD 5 mm PABBO BB/
PULPROG zgpg30
TD 65536
SOLVENT CDCl3
NS 1024
DS 4
SWH 24038.461 Hz
FIDRES 0.366798 Hz
AQ 1.3631488 sec
RG 2050
DW 20.800 usec
DE 6.50 usec
TE 300.1 K
D1 2.00000000 sec
D11 0.03000000 sec
TD0 1

======== CHANNEL f1 ========
SFO1 100.6605506 MHz
NUC1 13C
P1 11.00 usec
PLW1 48.00000000 W

======== CHANNEL f2 ========
SFO2 400.2816011 MHz
NUC2 1H
CPDPRG[2 waltz16
PCPD2 90.00 usec
PLW2 17.00000000 W
PLW12 0.24753000 W
PLW13 0.20050000 W

F2 - Processing parameters
SI 32768
SF 100.6504861 MHz
WDW EM
SSB 0
LB 1.00 Hz
GB 0
PC 1.40

^{1}H NMR (400 MHz, CD_3OD), Methyl (*E*)-3-(5-((4-((1-(2-(2-(2-((2-(2,6-dioxopiperidin-3-yl)-1-oxoisoindolin-4-yl)amino)-2-oxoethoxy)ethoxy)ethyl)-1H-1,2,3-triazol-4-yl)methoxy)benzyl)carbamoyl)-[1,1'-biphenyl]-3-yl)acrylate **(19)**

AVC-2-54.2.1
MeOD

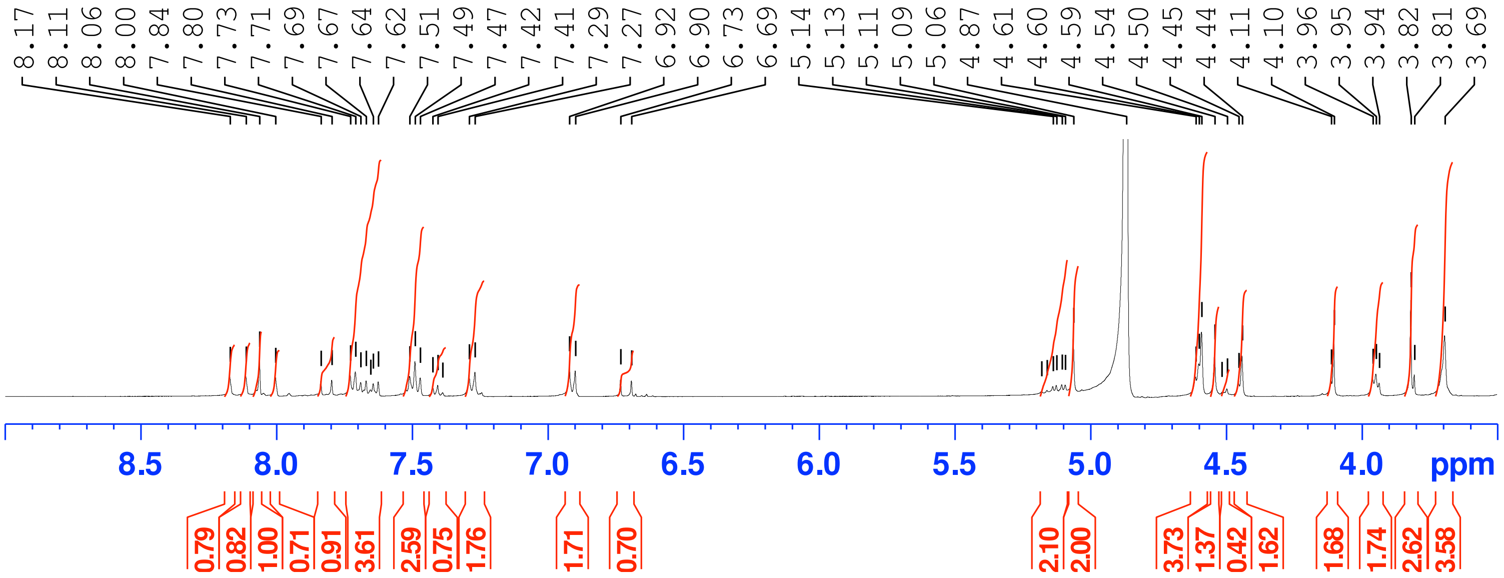

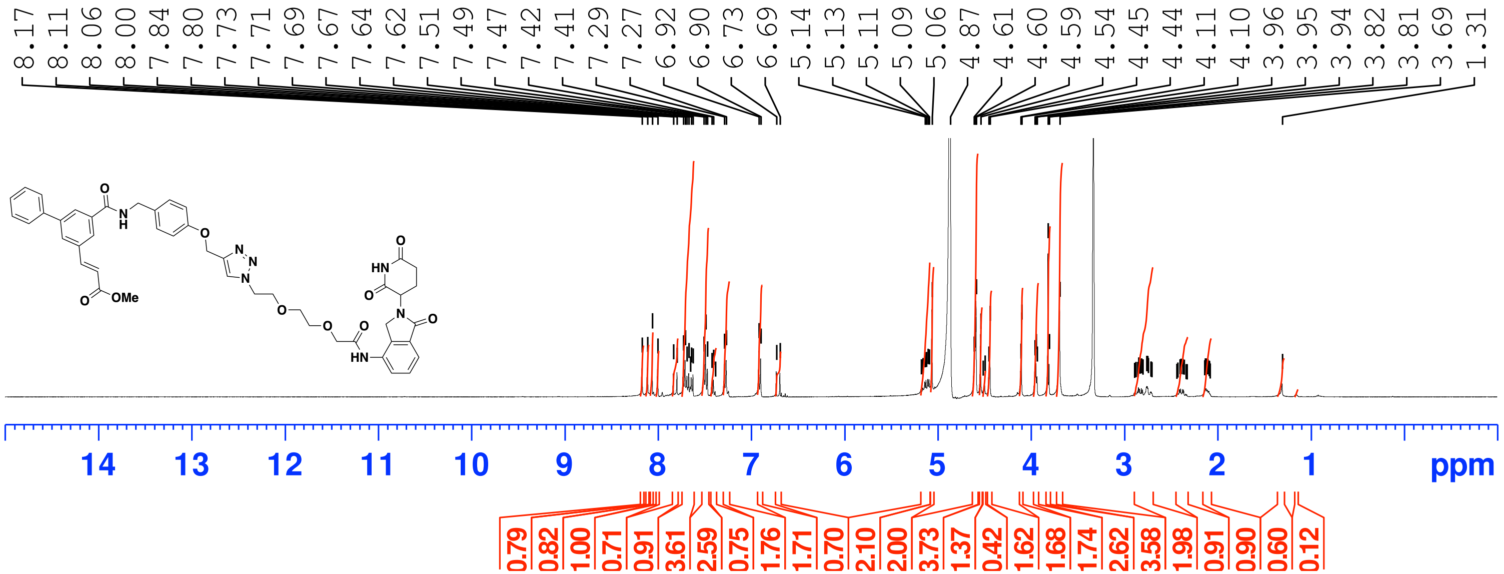

BRUKER

Current Data Parameters
NAME Jun23-2022-PTlab
EXPNO 20
PROCNO 1

F2 - Acquisition Parameters
Date_ 20220623
Time 13.03
INSTRUM spect
PROBHD 5 mm PABBO BB/
PULPROG zg30
TD 30046
SOLVENT MeOD
NS 112
DS 1
SWH 8012.820 Hz
FIDRES 0.266685 Hz
AQ 1.8748704 sec
RG 256
DW 62.400 usec
DE 6.50 usec
TE 300.0 K
D1 6.00000000 sec
TD0 1

======== CHANNEL f1 ========
SFO1 400.2821952 MHz
NUC1 1H
P1 10.86 usec
PLW1 17.00000000 W

F2 - Processing parameters
SI 131072
SF 400.2800000 MHz
WDW EM
SSB 0
LB 0.20 Hz
GB 0
PC 1.00

^{13}C NMR (100 MHz, CD_3OD), Methyl (*E*)-3-(5-((4-((1-(2-(2-(2-((2-(2,6-dioxopiperidin-3-yl)-1-oxoisoindolin-4-yl)amino)-2-oxoethoxy)ethoxy)ethyl)-1H-1,2,3-triazol-4-yl)methoxy)benzyl)carbamoyl)-[1,1'-biphenyl]-3-yl)acrylate **(19)**

AVC-2-54.2.1
MeOD

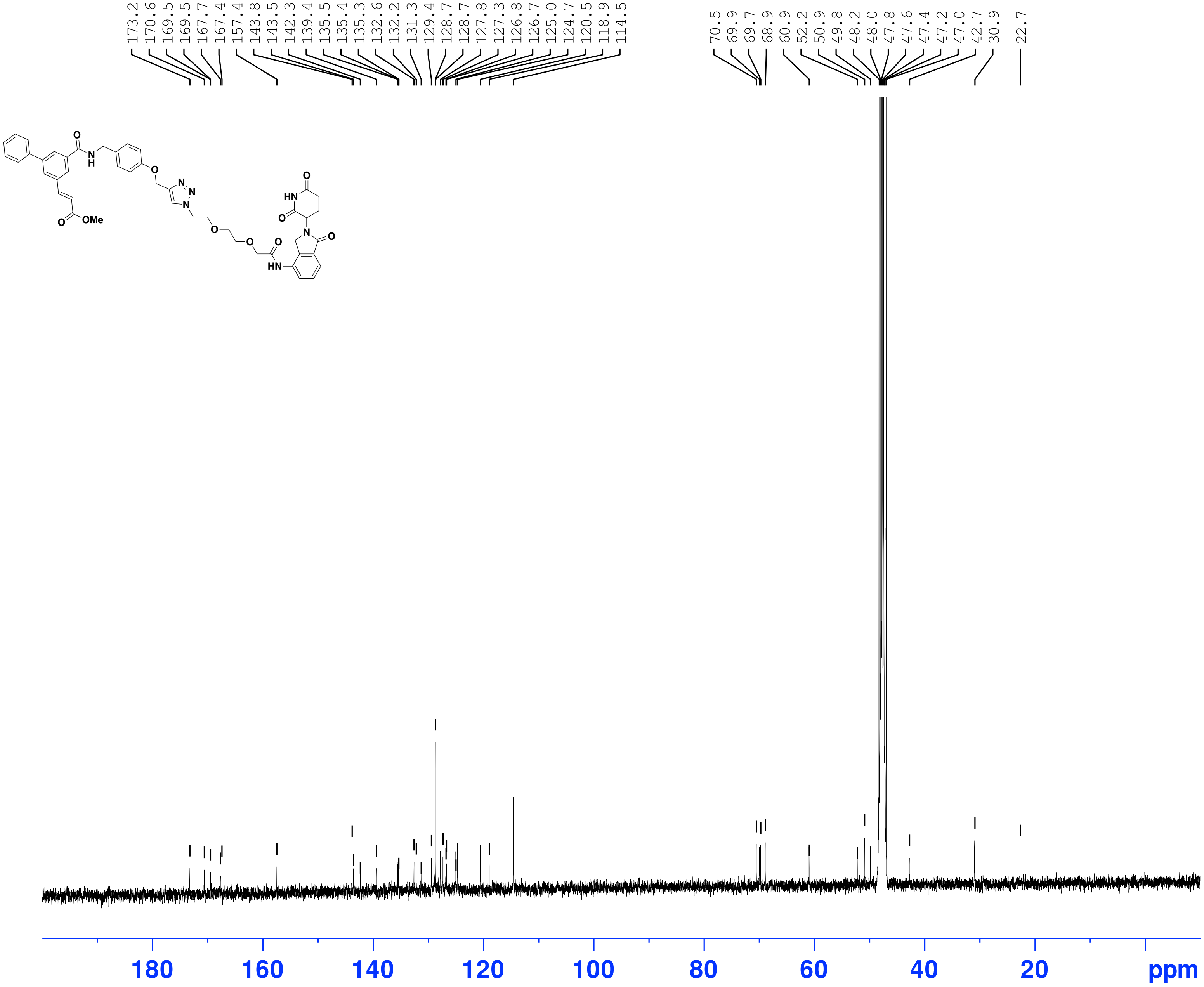

Current Data Parameters
NAME Jun24-2022-PTlab
EXPNO 10
PROCNO 1

F2 - Acquisition Parameters
Date_ 20220624
Time 10.24
INSTRUM spect
PROBHD 5 mm PABBO BB/
PULPROG zgpg30
TD 65536
SOLVENT MeOD
NS 2560
DS 4
SWH 24038.461 Hz
FIDRES 0.366798 Hz
AQ 1.3631488 sec
RG 2050
DW 20.800 usec
DE 6.50 usec
TE 300.4 K
D1 2.00000000 sec
D11 0.03000000 sec
TD0 1

======== CHANNEL f1 ========
SFO1 100.6605506 MHz
NUC1 13C
P1 11.00 usec
PLW1 48.00000000 W

======== CHANNEL f2 ========
SFO2 400.2816011 MHz
NUC2 1H
CPDPRG[2 waltz16
PCPD2 90.00 usec
PLW2 17.00000000 W
PLW12 0.24753000 W
PLW13 0.20050000 W

F2 - Processing parameters
SI 32768
SF 100.6504861 MHz
WDW EM
SSB 0
LB 1.00 Hz
GB 0
PC 1.40

^{1}H NMR (600 MHz, CD3OD), (E)-3-(5-((4-((1-(2-(2-(2-((2-(2,6-dioxopiperidin-3-yl)-1-oxoisoindolin-4-yl)amino)-2-oxoethoxy)ethoxy)ethyl)-1H-1,2,3-triazol-4-yl)methoxy)benzyl)carbamoyl)-[1,1'-biphenyl]-3-yl)acrylic acid (**5**)

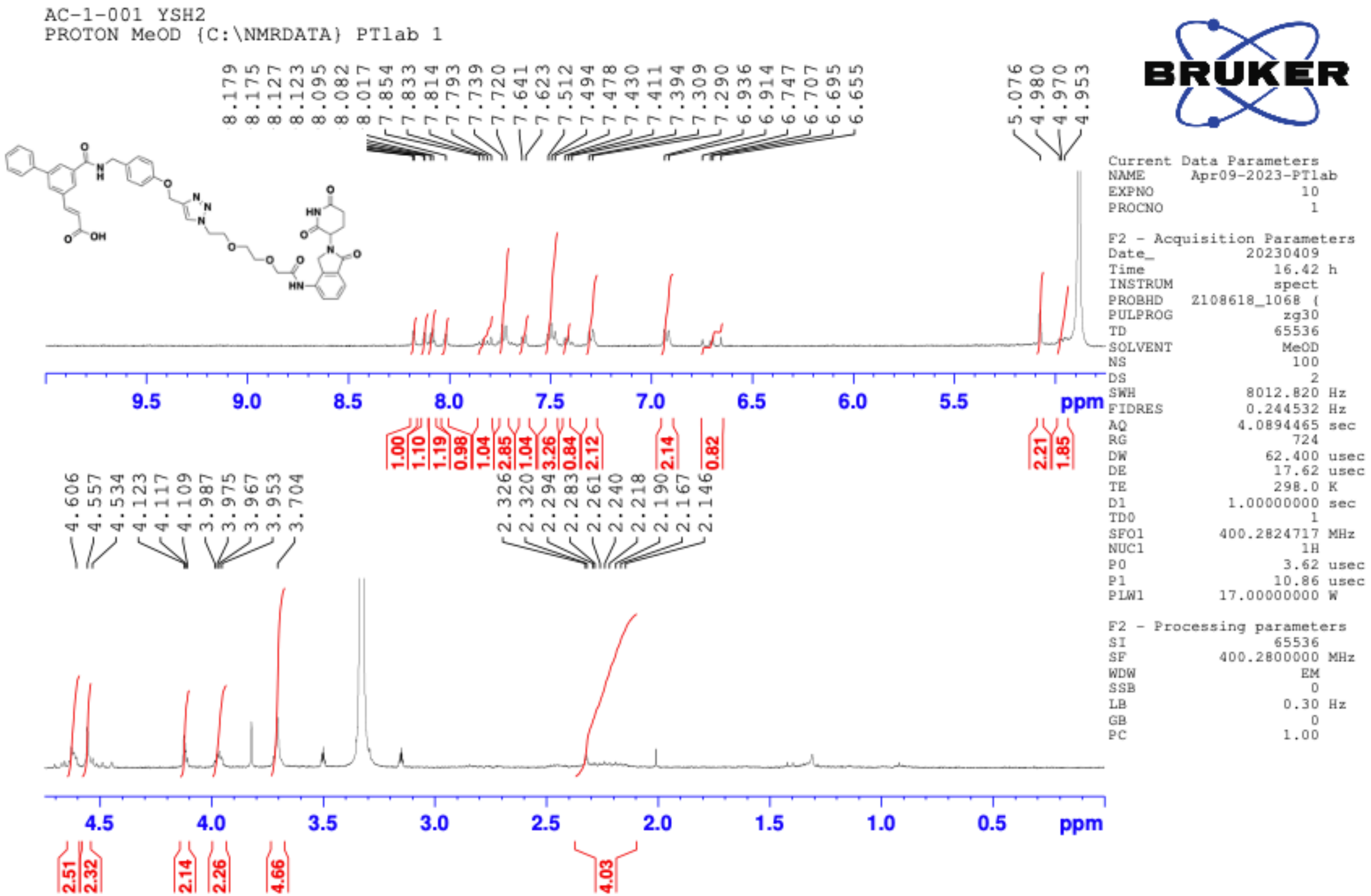

AC-1-001 YSH2
PROTON MeOD {C:\NMRDATA} PTlab 1

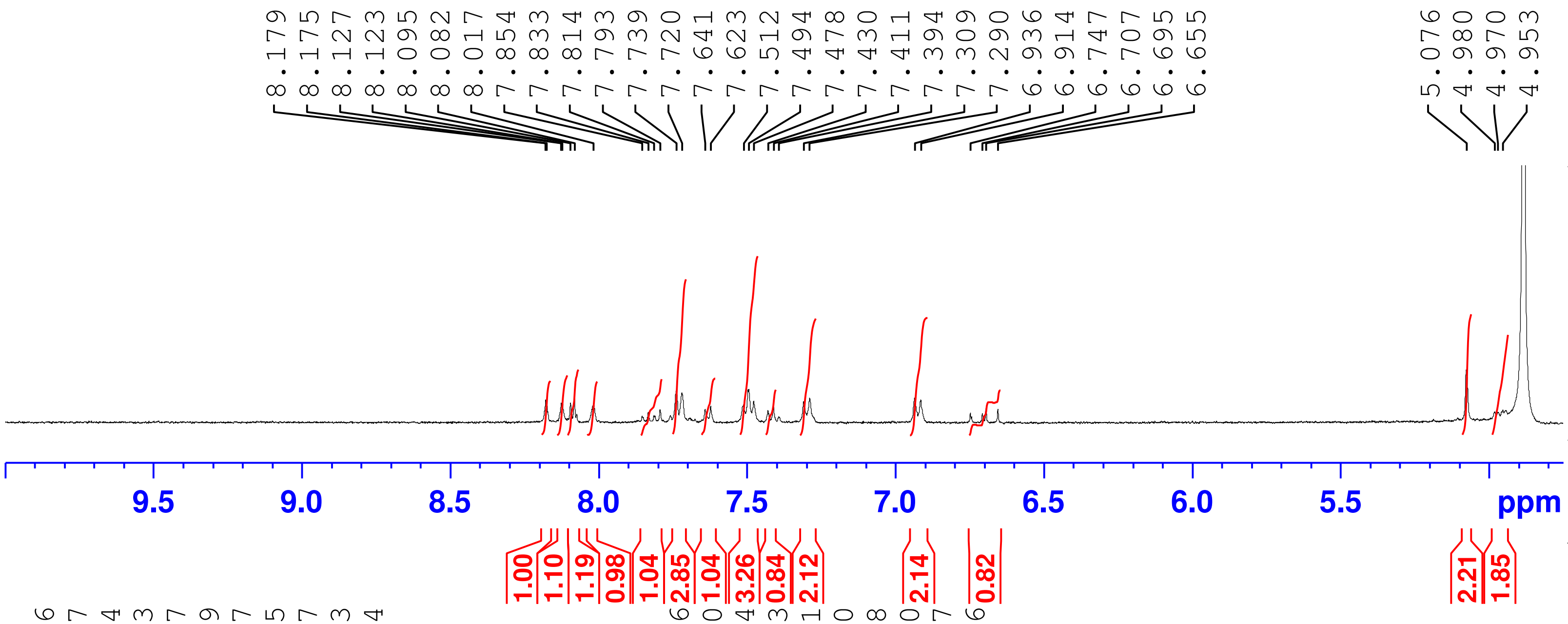

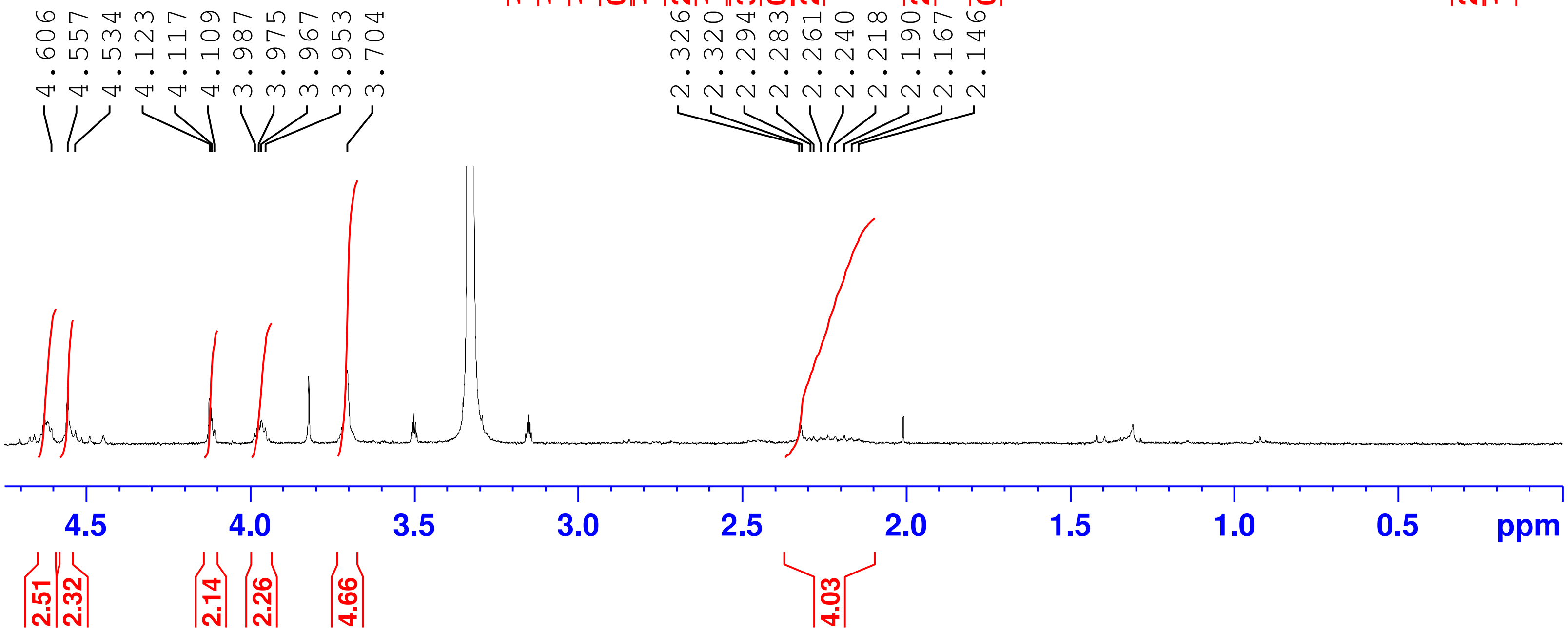

Current Data Parameters

NAME	Apr09-2023-PTlab
EXPNO	10
PROCNO	1

F2 - Acquisition Parameters

Date_	20230409	
Time	16.42	h
INSTRUM	spect	
PROBHD	Z108618_1068 (	
PULPROG	zg30	
TD	65536	
SOLVENT	MeOD	
NS	100	
DS	2	
SWH	8012.820	Hz
FIDRES	0.244532	Hz
AQ	4.0894465	sec
RG	724	
DW	62.400	usec
DE	17.62	usec
TE	298.0	K
D1	1.00000000	sec
TD0	1	
SFO1	400.2824717	MHz
NUC1	1H	
P0	3.62	usec
P1	10.86	usec
PLW1	17.00000000	W

F2 - Processing parameters

SI	65536	
SF	400.2800000	MHz
WDW	EM	
SSB	0	
LB	0.30	Hz
GB	0	
PC	1.00	

^{13}C NMR (150 MHz, CD_3OD), (*E*)-3-(5-((4-((1-(2-(2-(2-((2-(2,6-dioxopiperidin-3-yl)-1-oxoisoindolin-4-yl)amino)-2-oxoethoxy)ethoxy)ethyl)-1H-1,2,3-triazol-4-yl)methoxy)benzyl)carbamoyl)-[1,1'-biphenyl]-3-yl)acrylic acid **(5)**

AVC-2-66HH
MeOD

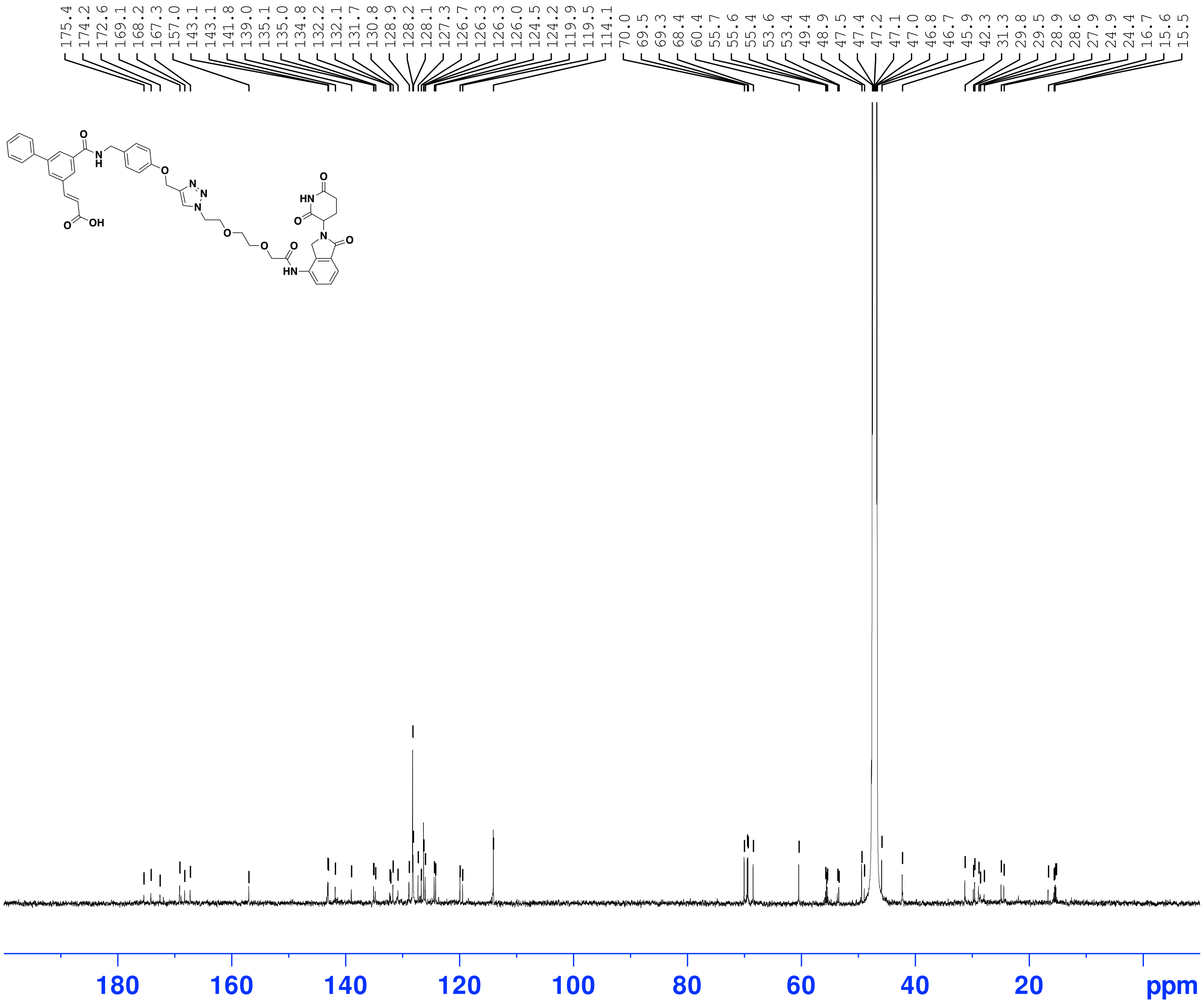

Current Data Parameters
NAME Jul18-2022-PTLab-600M
EXPNO 11
PROCNO 1

F2 - Acquisition Parameters
Date_ 20220718
Time 15.05 h
INSTRUM spect
PROBHD Z150313_0002 (
PULPROG zgpg30
TD 65536
SOLVENT MeOD
NS 3072
DS 4
SWH 36057.691 Hz
FIDRES 1.100393 Hz
AQ 0.9087659 sec
RG 194.35
DW 13.867 usec
DE 25.00 usec
TE 298.0 K
D1 2.00000000 sec
D11 0.03000000 sec
TD0 1
SFO1 150.8349112 MHz
NUC1 13C
P0 4.00 usec
P1 12.00 usec
PLW1 95.00000000 W
SFO2 599.8023992 MHz
NUC2 1H
CPDPRG[2 waltz16
PCPD2 70.00 usec
PLW2 7.09999990 W
PLW12 0.09273500 W
PLW13 0.04664500 W

F2 - Processing parameters
SI 65536
SF 150.8199049 MHz
WDW EM
SSB 0
LB 3.00 Hz
GB 0
PC 1.40

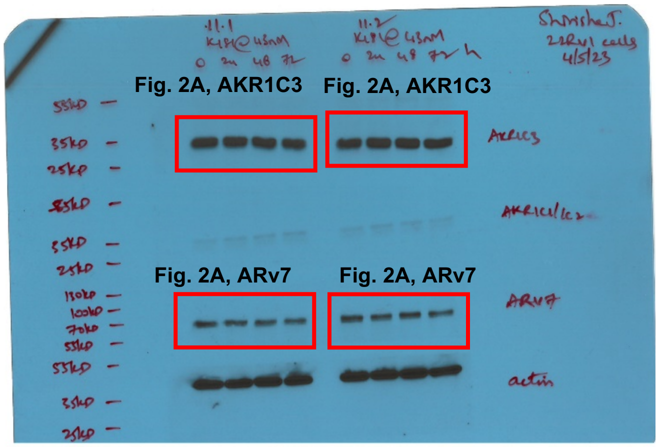
Fig. 2A, AKR1C3
Fig. 2A, AKR1C3
Fig. 2A, ARv7
Fig. 2A, ARv7

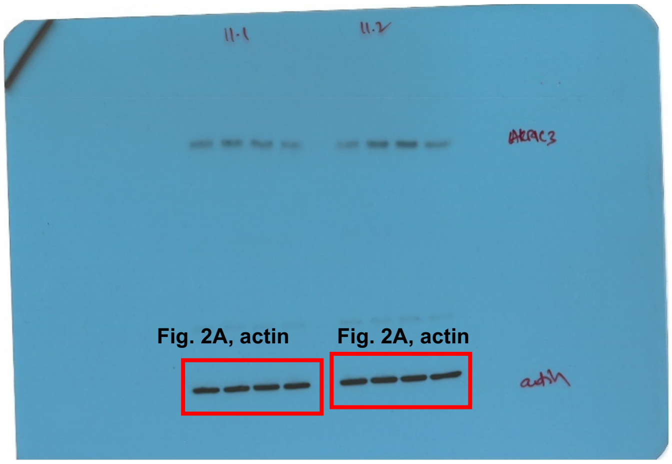
Fig. 2A, actin
Fig. 2A, actin

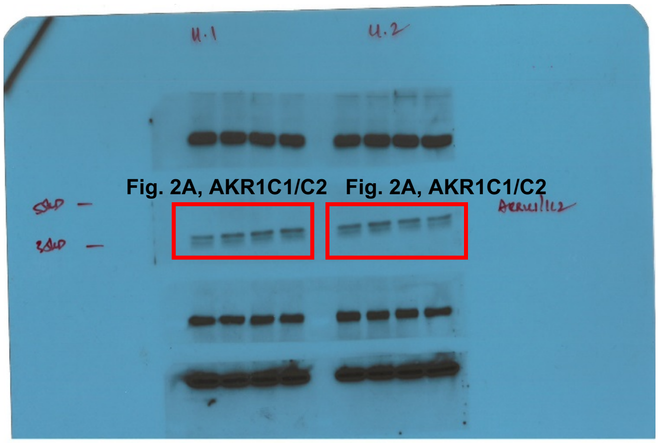
Fig. 2A, AKR1C1/C2
Fig. 2A, AKR1C1/C2

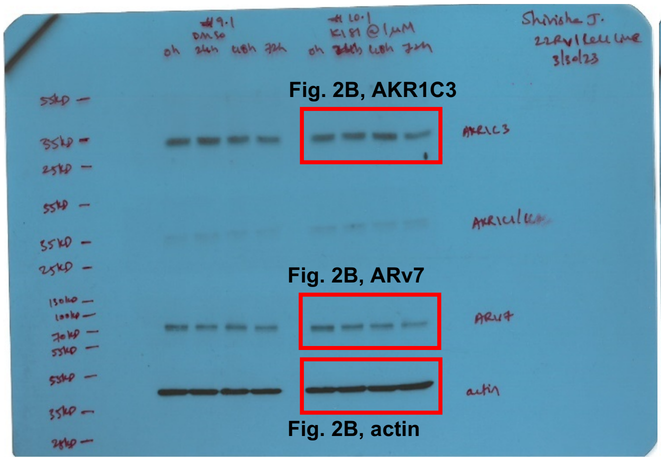
Fig. 2B, AKR1C3
Fig. 2B, ARv7
Fig. 2B, actin

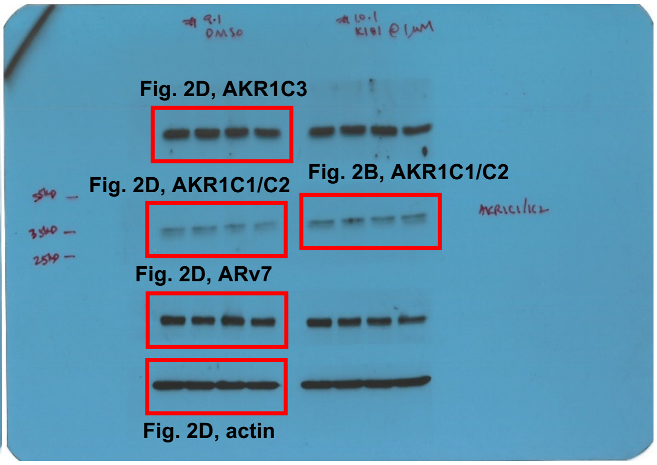
Fig. 2D, AKR1C3
Fig. 2D, AKR1C1/C2
Fig. 2B, AKR1C1/C2
Fig. 2D, ARv7
Fig. 2D, actin

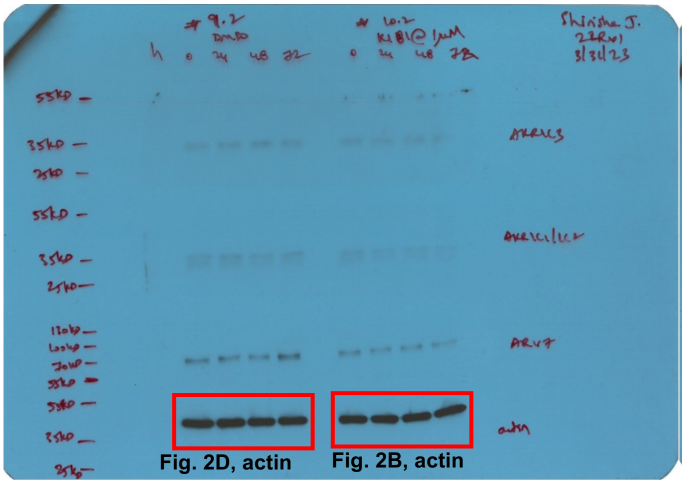
Fig. 2D, actin
Fig. 2B, actin

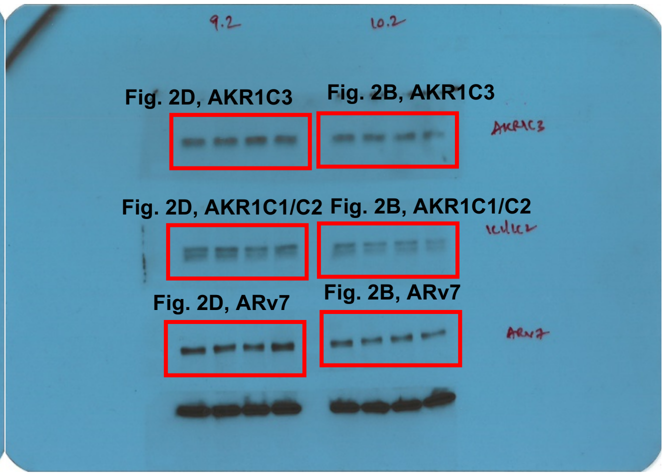
Fig. 2D, AKR1C3
Fig. 2B, AKR1C3
Fig. 2D, AKR1C1/C2
Fig. 2B, AKR1C1/C2
Fig. 2D, ARv7
Fig. 2B, ARv7

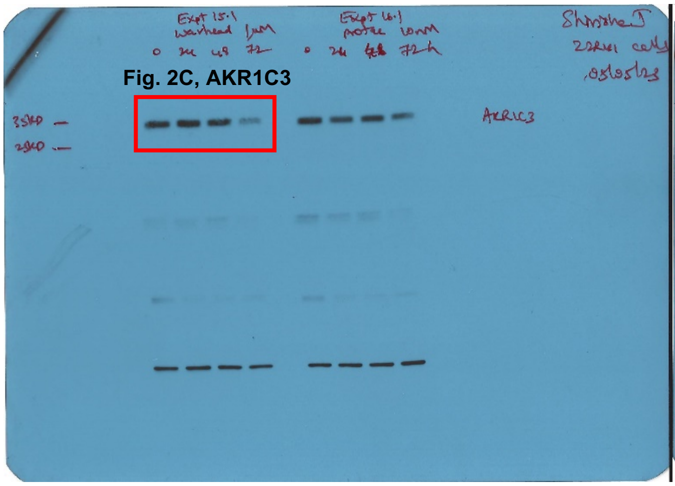
Fig. 2C, AKR1C3

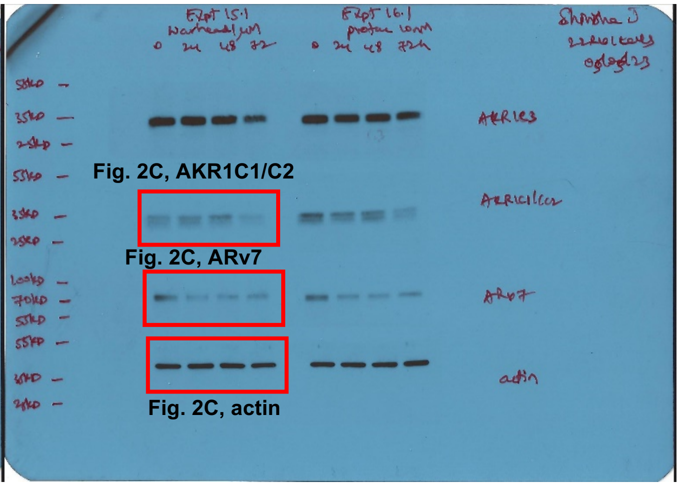
Fig. 2C, AKR1C1/C2
Fig. 2C, ARv7
Fig. 2C, actin

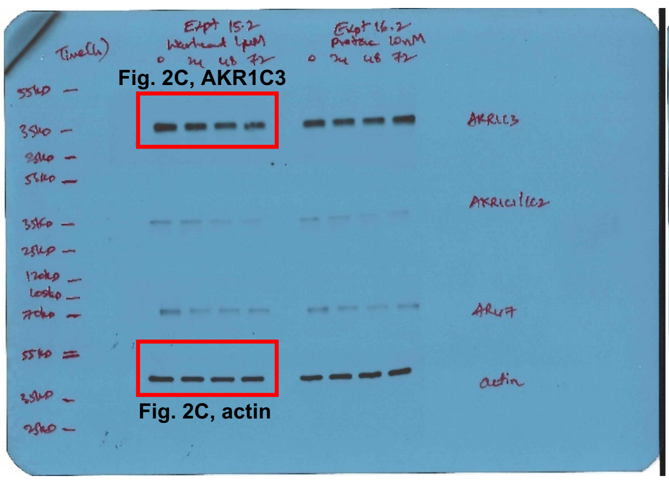
Fig. 2C, AKR1C3
Fig. 2C, actin

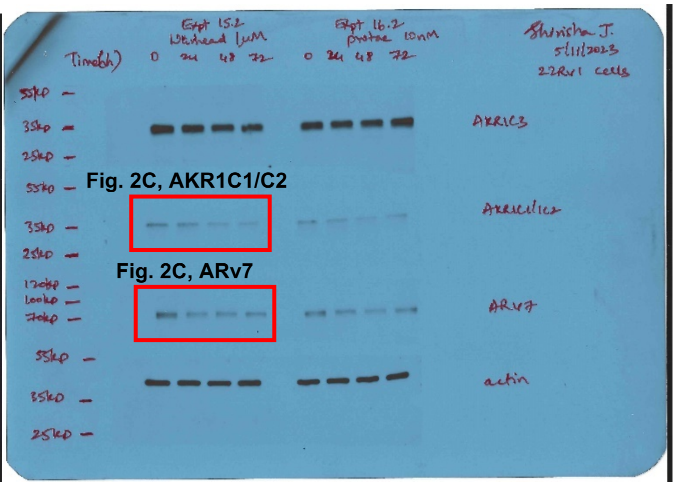
Fig. 2C, AKR1C1/C2
Fig. 2C, ARv7

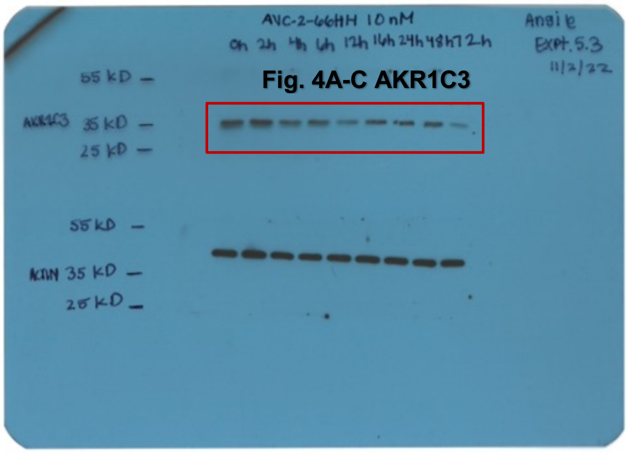
Fig. 4A-C AKR1C3
AVC-2-66HH 10 nM
0h 2h 4h 6h 12h 16h 24h 48h 72h
Angie
Expt. 5.3
11/2/22
55 kD
AKR1C3 35 kD
25 kD
55 kD
Actin 35 kD
25 kD

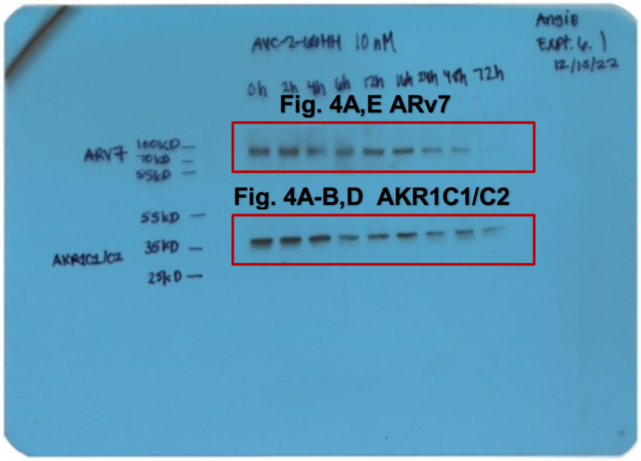
Fig. 4A,E ARv7
Fig. 4A-B,D AKR1C1/C2
AVC-2-66HH 10 nM
0h 2h 4h 6h 12h 16h 24h 48h 72h
Angie
Expt. 6.1
12/15/22
ARV7
100kD
70kD
55kD
55kD
AKR1C1/C2 35kD
25kD

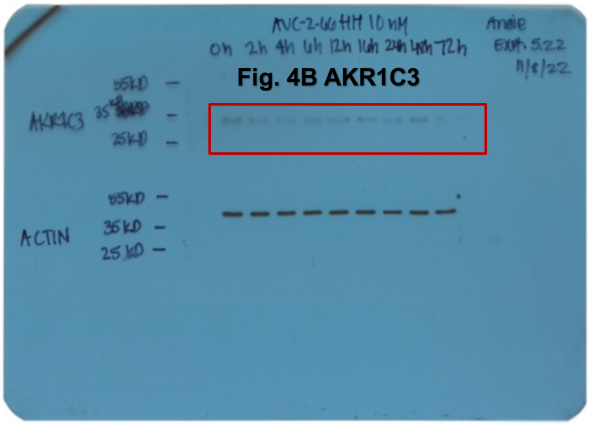
Fig. 4B AKR1C3
AVC-2-66HH 10 nM
0h 2h 4h 6h 12h 16h 24h 48h 72h
Angie
Expt. 5.22
11/8/22
55kD
AKR1C3 35
25kD
55kD
ACTIN 35kD
25kD

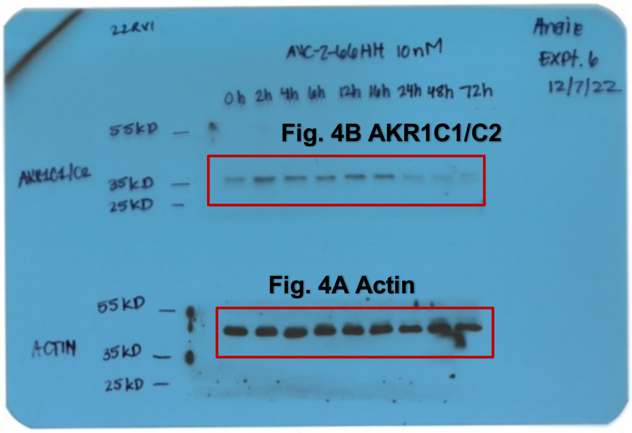
Fig. 4B AKR1C1/C2
Fig. 4A Actin
22RV1
AVC-2-66HH 10nM
0h 2h 4h 6h 12h 16h 24h 48h 72h
Angie
Expt. 6
12/7/22
55kD
AKR1C1/C2 35kD
25kD
55kD
ACTIN 35kD
25kD

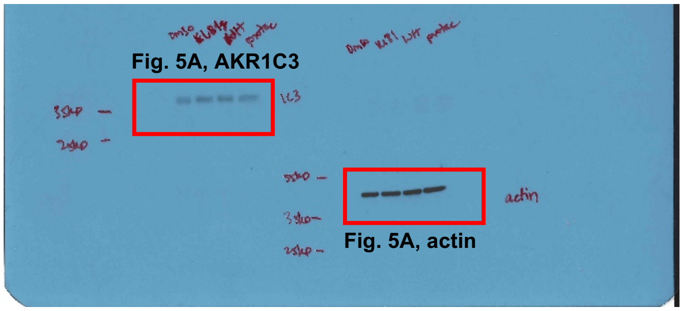
Fig. 5A, AKR1C3
Fig. 5A, actin
actin
DMSO
protac

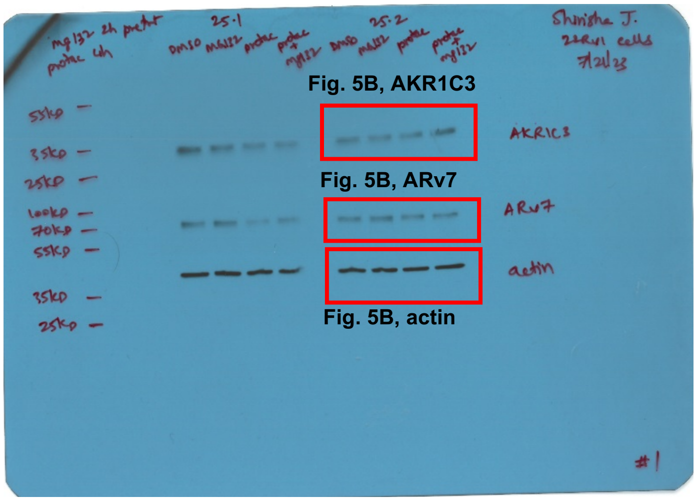
Fig. 5B, AKR1C3
Fig. 5B, ARv7
Fig. 5B, actin
AKR1C3
ARv7
actin
Shrisha J.
22Rv1 cells
7/21/23
#1
