# Supplementary material for: Discovery of an Aldo-Keto reductase 1C3 (AKR1C3) degrader
Source: Commun Chem. 2024 Apr 29;7:95. doi: 10.1038/s42004-024-01177-4 (PMC11059152; doi:10.1038/s42004-024-01177-4)
Supplement: Supplementary file 2 — Description of Additional Supplementary Files [file 42004_2024_1177_MOESM2_ESM.pdf]

Description of Additional Supplementary Files

File name: Supplementary Data 1

Description: Source data for all graphs
