# Supplementary material for: Discovery of an Aldo-Keto reductase 1C3 (AKR1C3) degrader
Source: Commun Chem. 2024 Apr 29;7:95. doi: 10.1038/s42004-024-01177-4 (PMC11059152; doi:10.1038/s42004-024-01177-4)
Supplement: Supplementary file 4 — Reporting Summary [file 42004_2024_1177_MOESM4_ESM.pdf]

nature portfolio

Corresponding author(s): Paul C. Trippier

Last updated by author(s): Mar 29, 2024

Reporting Summary

Nature Portfolio wishes to improve the reproducibility of the work that we publish. This form provides structure for consistency and transparency in reporting. For further information on Nature Portfolio policies, see our Editorial Policies and the Editorial Policy Checklist.

Statistics

For all statistical analyses, confirm that the following items are present in the figure legend, table legend, main text, or Methods section.

n/a	Confirmed	
☐	☒	The exact sample size (*n*) for each experimental group/condition, given as a discrete number and unit of measurement
☐	☒	A statement on whether measurements were taken from distinct samples or whether the same sample was measured repeatedly
☐	☒	The statistical test(s) used AND whether they are one- or two-sided<br>*Only common tests should be described solely by name; describe more complex techniques in the Methods section.*
☒	☐	A description of all covariates tested
☒	☐	A description of any assumptions or corrections, such as tests of normality and adjustment for multiple comparisons
☐	☒	A full description of the statistical parameters including central tendency (e.g. means) or other basic estimates (e.g. regression coefficient) AND variation (e.g. standard deviation) or associated estimates of uncertainty (e.g. confidence intervals)
☒	☐	For null hypothesis testing, the test statistic (e.g. *F*, *t*, *r*) with confidence intervals, effect sizes, degrees of freedom and *P* value noted<br>*Give P values as exact values whenever suitable.*
☒	☐	For Bayesian analysis, information on the choice of priors and Markov chain Monte Carlo settings
☒	☐	For hierarchical and complex designs, identification of the appropriate level for tests and full reporting of outcomes
☒	☐	Estimates of effect sizes (e.g. Cohen's *d*, Pearson's *r*), indicating how they were calculated

Our web collection on statistics for biologists contains articles on many of the points above.

Software and code

Policy information about availability of computer code

Data collection	Topspin 4.4, Agilent MassHunter, SeeSAR 12.1
Data analysis	Prism 10, Image J, MestReNova-LITECDE, SeeSAR 12.1

For manuscripts utilizing custom algorithms or software that are central to the research but not yet described in published literature, software must be made available to editors and reviewers. We strongly encourage code deposition in a community repository (e.g. GitHub). See the Nature Portfolio guidelines for submitting code & software for further information.

Data

Policy information about availability of data

All manuscripts must include a data availability statement. This statement should provide the following information, where applicable:

- Accession codes, unique identifiers, or web links for publicly available datasets
- A description of any restrictions on data availability
- For clinical datasets or third party data, please ensure that the statement adheres to our policy

Supporting Information includes experimental details, materials, methods, source data, characterization, supplementary figures, and 1H, 13C NMR spectra. Raw fid files are available on request to the corresponding author.

Human research participants

Policy information about studies involving human research participants and Sex and Gender in Research.

Reporting on sex and gender	Not applicable
Population characteristics	Not applicable
Recruitment	Not applicable
Ethics oversight	Not applicable

Note that full information on the approval of the study protocol must also be provided in the manuscript.

Field-specific reporting

Please select the one below that is the best fit for your research. If you are not sure, read the appropriate sections before making your selection.

☒ Life sciences ☐ Behavioural & social sciences ☐ Ecological, evolutionary & environmental sciences

For a reference copy of the document with all sections, see nature.com/documents/nr-reporting-summary-flat.pdf

Life sciences study design

All studies must disclose on these points even when the disclosure is negative.

Sample size	No studies requiring sample size calculations. All experiments in at least duplicate or higher. See replication below.
Data exclusions	Two experiments were excluded from the MG132 experiments as no effect was observed for PROTAC, MG132, or combination.
Replication	Western blots represent at least two replicates. Cell viability experiments represent mean of at least three experiments run in triplicate.
Randomization	Not applicable.
Blinding	No blinding.

Reporting for specific materials, systems and methods

We require information from authors about some types of materials, experimental systems and methods used in many studies. Here, indicate whether each material, system or method listed is relevant to your study. If you are not sure if a list item applies to your research, read the appropriate section before selecting a response.

Materials & experimental systems

n/a	Involved in the study	
☐	☒	Antibodies
☐	☒	Eukaryotic cell lines
☒	☐	Palaeontology and archaeology
☒	☐	Animals and other organisms
☒	☐	Clinical data
☒	☐	Dual use research of concern

Methods

n/a	Involved in the study	
☒	☐	ChIP-seq
☒	☐	Flow cytometry
☒	☐	MRI-based neuroimaging

Antibodies

Antibodies used	AKR1C3 (mouse mAb, 1:200, Sigma, A6229), AKR1C1/C2 (rabbit mAb, 1:500, Abcam, ab179448), ARv7 (rabbit mAb, 1:500, Abcam, ab198394) and actin (mouse mAb, 1:1,000, ThermoFisher, MA5-11869).
Validation	See manufacturers website. And Mol. Cancer. Ther. 2018, 17, 1833-1845.

Eukaryotic cell lines

Policy information about cell lines and Sex and Gender in Research

Cell line source(s)	ATCC or Dr. Trevor Penning (LNCaP1C3, J Steroid Biochem Mol Biol 2012, 130, 7-15).
Authentication	Authenticated by ATCC at time of purchase. Cell line passage use was limited to no greater than passage 9.
Mycoplasma contamination	Cell lines tested negative for mycoplasma contamination using the MycoAlert mycoplasma detection kit as per the manufacturer's instructions (Lonza, LT07-318)
Commonly misidentified lines (See ICLAC register)	No commonly misidentified cell lines were used herein.
